# Examining the feasibility and preliminary effects of resistance exercise training and creatine supplementation in individuals treated for colorectal cancer

**DOI:** 10.1371/journal.pone.0353630

**Published:** 2026-07-15

**Authors:** Ciaran M. Fairman, Alex M. Brooks, Darren G. Candow, Kylah E. Jackson, Genevieve Bottone, Brett Scott, Kenneth S. Anderson, Katie R. Hirsch, Thomas D. Cardaci, Brandon N. VanderVeen, Christine E. Blake, Tiejun Zhang, Jiajia Zhang, E. Angela Murphy

**Affiliations:** 1 Department of Exercise Science, University of South Carolina, Columbia, South Carolina, United States of America; 2 Faculty of Kinesiology and Health Studies, University of Regina, Regina, Saskatchewan, Canada; 3 Department of Pathology, Microbiology & Immunology, University of South Carolina, Columbia, South Carolina, United States of America; 4 Department of Health and Exercise Science, Colorado State University, Fort Collins, Colorado, United States of America; 5 Division of Medical Oncology, Department of Medicine, University of Colorado School of Medicine, Aurora, Colorado, United States of America; 6 University of Colorado Comprehensive Cancer Center, Aurora, Colorado, United States of America; 7 Department of Cancer Biology, Wake Forest University School of Medicine, United States of America; 8 Department of Health Promotion, Education and Behavior, University of South Carolina, United States of America; 9 Department of Epidemiology and Biostatistics, Arnold School of Public Health, University of South Carolina, United States of America; Dynamical Business & Science Society - DBSS International SAS, COLOMBIA

## Abstract

**Purpose:**

The purpose of this trial was to assess the feasibility, acceptability and safety of a 10-week hybrid (in-clinic and virtual) resistance exercise training RET program with or without CrM in individuals treated for colorectal cancer.

**Methods:**

Twenty-seven participants were randomized to RET plus 5 grams/day of CrM (EXSUPP; n = 13) or 5 grams/day corn-starch maltodextrin placebo (EXPLA; n = 14). RET was performed three times per week. Feasibility was assessed through recruitment, retention and fidelity (percentage of prescribed RET and supplementation completed). Acceptability was evaluated using a 5-point Likert scale, and safety was monitored through adverse event reporting. Secondary outcomes (body composition, muscular strength, physical function) were assessed pre- and post-intervention using baseline-adjusted ANCOVA models and focus group interviews.

**Results:**

Registry-based recruitment identified 1378 potentially eligible individuals and 27 of 410 assessed (6.6%) enrolled, highlighting recruitment challenges. Retention was high (24/27; 88.9%). Adherence to RET and supplementation was strong (both > 85%) with no serious adverse events reported. Participants reported high acceptability for both in person and virtual components. No significant between group differences were observed for secondary outcomes; however, both groups demonstrated modest improvements in muscular strength and short physical performance battery (SPPB) scores.

**Conclusions:**

A hybrid RET program with CrM was feasible, acceptable and well tolerated in individuals with colorectal cancer who were previously treated with chemotherapy.

**Implications for Cancer Survivors:**

Hybrid supervised RET is safe, acceptable, and associated with modest improvements in strength and physical function among individuals treated for colorectal cancer. While creatine supplementation did not demonstrate clear additive effects in this pilot trial, further adequately powered studies are warranted.

**Trial Registration:** NCT06420726.

## Introduction

Colorectal cancer is the third most common cancer worldwide [1–3]. As the number of individuals surviving the disease in the long-term increases, so does the number of individuals who are burdened by the persistent off-target effects of anti-cancer therapies [1–4]. Mounting evidence highlights the consequences of cancer-related treatments which contribute to accelerated aging, such as the progressive loss of muscle mass and functional ability [5–7]. Alterations in molecular pathways that govern muscle homeostasis as a result of cancer and its treatments can exacerbate muscle dysfunction [8,9]. In colorectal cancer, loss of muscle during treatment is associated with a two- to four-fold increase in mortality risk and reduced quality of life [10–12]. Further, more than half of individuals treated for cancer fail to recover pre-operative physical activity levels within six months post-diagnosis, thereby compounding declines in muscle health [13]. Cancer treatments therefore exacerbate skeletal muscle loss and accelerate the trajectory toward disability and mortality, highlighting the importance of targeted interventions to preserve muscle mass and function in individuals treated for cancer.

Resistance exercise training (RET) is the most widely recommended strategy to address declines in musculoskeletal health in oncology settings [14–18]. However, there is increasing recognition that the physiological impact of cancer and its treatments may blunt the response to RET [6,7,19]. The results of our recent meta-analysis indicated minimal gains in whole-body lean mass (~0.4 kg) with RET in individuals with cancer [20], which contrasts with findings from a separate meta-analysis in apparently healthy older adults reporting ~1.1 kg improvements from similar interventions [21]. It is conservatively estimated that 50% of lean mass is skeletal muscle mass [22]. While differences in study design and populations preclude direct comparisons, these data suggest that individuals with cancer may experience attenuated adaptations to RET. Several hypotheses have been proposed for this attenuated response, including treatment-induced anabolic resistance, systemic inflammation, and nutritional deficiencies, in addition to limitations in program design [15,19,23–25]. Taken collectively, these findings suggest that RET alone may be insufficient to overcome the multifactorial mechanisms driving low muscle mass and function in cancer survivors, highlighting the need to investigate adjunctive strategies to augment adaptations to RET [23].

Creatine (CrM) is a nitrogen-containing compound that plays a central role in rapid energy provision during RET [26]. Higher intramuscular phosphocreatine stores increase ATP availability, enhancing training capacity and promoting greater physiological adaptations in lean mass, muscle thickness, strength, and functional ability [26–33]. CrM is one of the most extensively studied nutritional supplements, with over 1,000 studies confirming its safety and efficacy across the lifespan [34–38]. Of particular relevance, aging is associated with progressive losses in lean mass, muscle size, strength, and functional ability (that are typically exacerbated in cancer contexts) [6,7,39,40]. In older adult populations, there is consistent, strong evidence that CrM can enhance RET adaptations, by augmenting lean mass, limb muscle accretion, strength and physical function to a greater degree relative to RET alone [36,41–43]. In individuals with cancer, a small but growing number of studies have combined CrM with RET, with early work examining functional and body composition outcomes across a limited range of cancer populations [44,45]. To date, however, this evidence base remains limited, and to our knowledge it has not extended to individuals treated for colorectal cancer, a population in which such losses are frequent and in whom the feasibility of a combined CrM and RET approach has yet to be established.

Despite a strong rationale, evidence on CrM in individuals treated for cancer remains scarce, and no trials have specifically combined CrM with RET in colorectal cancer survivors [23,46]. To address this gap, we sought to conduct a pilot randomized controlled trial (RCT), examining the effects of a 10-week RET intervention with CrM, relative to RET with a placebo in individuals treated for colorectal cancer. The primary aim was to evaluate the feasibility, safety, and acceptability of a 10-week RCT comparing RET with or without CrM. Secondary aims were to estimate mean changes and compare between-group differences in muscular strength, body composition, physical function, and health-related quality of life.

## Methods

### Trial design

This was a two-arm, double-blind, randomized controlled pilot trial comparing CrM versus placebo in combination with supervised RET for individuals previously treated for colorectal cancer. The recruitment target was 40 participants, with 20 randomized to RET with CrM supplementation (EXSUPP) and 20 randomized to RET with placebo (EXPLA). Both groups completed a 10-week supervised RET program. This manuscript was prepared and reported in accordance with the Consolidated Standards of Reporting Trials (CONSORT) 2010 Statement extension for randomized pilot and feasibility trials. (S1 File CONSORT Checklist).

The study protocol was approved by the University of South Carolina Institutional Review Board (ID: Pro00127362), and all participants provided written informed consent prior to any study activity. The trial was registered at ClinicalTrials.gov (NCT06420726) prior to participant enrollment. The primary aim of the trial was to evaluate feasibility and acceptability, assessed through recruitment, retention, adherence, and safety measured through adverse events. The secondary aim of the trial was to estimate baseline-adjusted between-group differences in secondary outcomes (muscular strength, body composition, physical function, and quality of life). Success of the intervention and criteria to progress to a larger trial were based on the following:

1. *Recruitment:* If the study achieved the recruitment goal of n = 40 within 24 months. The sample size was selected to provide estimates of feasibility outcomes and to inform sample size calculations for a larger randomized trial [47,48].
2. *Retention:* If ≥75% of participants returned for post-intervention testing.
3. *Adherence:* If ≥75% adherence to the supervised RET sessions and ≥75% adherence to CrM/placebo supplementation, verified through attendance logs and supplement tracking.
4. *Adverse events:* If no serious adverse events attributable to the intervention were observed.

### Changes to study protocol

Several changes to the study were made since its original design and pre-registration that are worthy of note in the context of the feasibility of the intervention, and the findings from this trial. Specifically, changes were made to 1) the eligibility criteria; 2) the outcome assessments and intervention delivery, and 3) the supplementation protocol. Briefly, the original study design was focused on enrolling individuals who were confirmed sarcopenic (through assessment of dual-energy X-ray absorptiometry and physical function), who were ≥12 months post treatment. After 12-months of recruitment, only one individual was confirmed for sarcopenia through SARC-F screening [49]. Consequently, the eligibility criteria were updated to remove 1) the time restriction (≥12 months post treatment) and 2) sarcopenia as an inclusion criterion to enhance accrual. Further, prior chemotherapy exposure was added as an inclusion criterion given the association with chemotherapy and reduced muscle mass and function to remain focused on a vulnerable population [8,10,50–52].

**Fig 1:**
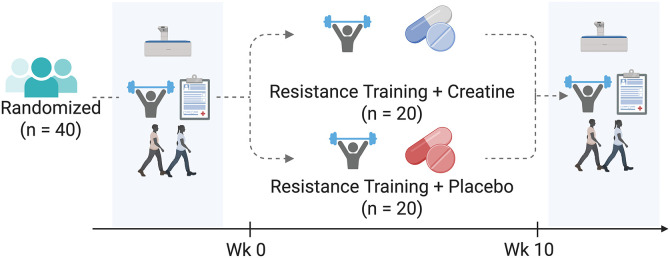
Overview of study design.

Changes were also made to the outcome assessments and intervention delivery, due to logistics and participant interest. The study site did not have a leg press, so the leg extension was used for lower body repetition maximum testing instead. Further, the intervention was modified to 1) condense two weeks of a familiarization phase into one, 2) replace a morning session offering (6.30am-7.30am) with an evening session offering (6.30–7.30 pm), and 3) switch the third weekly session to an online exercise session to reduce travel burden and enhance accessibility. Lastly, the initial protocol outlined participants receiving 0.10g/kilograms/day of CrM. However, the study team opted for a standard dose of 5 grams/day to reflect real-world applications, where individuals will likely follow consumer label directions. Expanded details of the original protocol, changes made, and rationale for these changes are outlined in S2 File. A copy of the original protocol approved by the institution’s IRB board is in S3 File. An overview of the study design is in Fig 1.

### Participants

#### Eligibility criteria.

The initial protocol aimed to enroll adults at least 12 months post-treatment for colorectal cancer who were treated with chemotherapy for their cancer. (see changes “changes to study protocol” above for amendments to the original eligibility criteria). Participants were excluded if they (1) were receiving active treatment for their cancer; (2) had a musculoskeletal, cardiovascular, neurological, or psychological condition that precluded participation in RET; (3) had been participating in structured RET two or more times per week for the past 6 months; (4) were currently taking supplements containing CrM within 4 weeks prior to the start of the RCT; or (5) were receiving medications that might have altered body composition (e.g., metformin, corticosteroids).

### Participant recruitment and screening

This study was conducted through the University of South Carolina in collaboration with Prisma Health, a clinical affiliate of the University of South Carolina. The primary source of recruitment was the cancer registry databases at Prisma Health. A waiver of Health Insurance Portability and Accountability Act authorization was approved by the Institutional Review Board, allowing invitations signed by treating medical and radiation oncologists to be mailed to potential participants. The intervention was delivered in waves of 3–8 participants at two rehabilitation clinics affiliated with the hospital. To minimize loss of interest or ineligibility due to extended wait times, invitations were mailed in batches timed to coincide with study start-up (for the first wave) and following the completion of each wave (for subsequent waves). Secondary recruitment strategies included the display of study flyers in oncology and surgical clinics within the Prisma Health system.

Invitations were sent using an “opt-out” strategy, whereby individuals were provided study staff contact information and instructed to call if they did not wish to be contacted about participation. Approximately one week after the mailings were sent, study staff contacted potential participants by phone to provide additional information about the study’s goals, risks, and benefits, and to determine initial eligibility. If phone screening was successful, participants were scheduled for an in-person visit at the University of South Carolina laboratory for informed consent review and signing, and baseline assessments.

### Randomization

Participants were randomly assigned in a 1:1 ratio to either the CrM group (EXSUPP) or the placebo group (EXPLA) using a computer-generated random allocation sequence created by the study statistician. Allocation concealment was implemented using sequentially numbered, opaque sealed envelopes prepared by a member of the research staff who was not involved in participant enrollment, outcome assessment, or data analysis. Investigators, outcome assessors, and data analysts remained blinded to the allocation sequence and group assignment throughout the study.

### Intervention

Irrespective of group assignment, all participants completed 10-weeks of supervised RET. The Consensus on Exercise Reporting Template was used to outline the exercise intervention components [53]. Sessions were delivered in a hybrid format, combining in-clinic and live, virtual exercise sessions. Two sessions per week were completed at one of two community clinics affiliated with the local hospital system, and one session per week was conducted remotely via Zoom. In-clinic sessions were delivered in a small-group format (maximum of eight participants) with a 1:1 participant-to-instructor ratio to ensure individualized supervision. Sessions were delivered by student research assistants with internal training in exercise oncology by the principal investigator, who has more than a decade of experience designing and delivering RET interventions in oncology.

The intervention began with a 1-week familiarization phase (two visits). During this period, participants were introduced to safe exercise technique, individualized exercise selection, and self-determined repetitions in reserve to establish baseline training loads. Familiarization also included structured training on how to participate in remote sessions, including instruction on using Zoom, logging into sessions, setting up the camera and exercise space, and troubleshooting common technical issues. Participants practiced connecting virtually during the familiarization phase, with staff available to ensure comfort and confidence before the intervention phase began.

Following familiarization, participants completed 28 supervised sessions over 10 weeks (three per week, separated by ≥48 hours). Each session lasted approximately 60 minutes and included eight multi-joint, compound exercises targeting all major muscle groups (e.g., squat, chest press, seated row, overhead press). Exercises were tailored based on baseline strength, comorbidities, and individual movement patterns. Training loads were progressed by ~5–10% for upper-body exercises and ~10–15% for lower-body exercises, provided safe technique was demonstrated [54]. Decisions regarding grouping and progression were made by study staff delivering the intervention. Exercise progression was achieved by increasing load or modifying body position and range of motion, and intensity was monitored using self-determined repetitions in reserve (SD RIR) [55–60]. Participants were introduced to the SD RIR scale during the familiarization phase, with detailed instructions and anchoring procedures provided to standardize interpretation. We and others have successfully used this approach in trials of RET in different tumor types and have yielded meaningful improvements in physical function, muscular strength and body composition [44,61–64]. An overview of the planned sets/reps for the duration of the intervention is outlined in S2 File.

Remote sessions were delivered in one-on-one sessions with a member of the study team. Prior to the first remote exercise session, participants were provided with a pair of adjustable kettlebells that could be manipulated to provide resistance ranging from ~5–40 kg, as well as a set of four resistance bands with varying resistance levels for use during exercise sessions. For safety monitoring during remote sessions, participants were required to keep cameras on throughout the session, and instructors provided continuous live supervision. Clear communication protocols were established, including verbal check-ins during each exercise set and immediate reporting of any adverse symptoms or difficulties. Any adverse events that occurred during remote or in-clinic sessions were documented in real time, including a description of the event, its severity, and its potential relation to the intervention. Events were reported to the study PI and adjudicated according to the same standardized protocol across both delivery formats.

Adherence was tracked through session attendance logs, and fidelity to the protocol was maintained via standardized supervisor training and ongoing monitoring. We also quantified adherence using the exercise-relative dose intensity (RDI) framework [65]. This approach captures the ratio of completed to prescribed training volume (sets x repetitions x load) across the intervention and accounts for dose modifications, interruptions, or missed sessions [65]. Lastly, de-identified training logs for each participant can be found in S4 File.

*Supplementation*. Participants in the EXSUPP group received CrM (CrM; Creapure^®^, AlzChem, Trostberg GmbH, Germany). During the first week following baseline assessment, participants completed a loading phase consisting of 20 grams/day for 7 consecutive days, divided into four 5 gram doses consumed throughout the day. Following the loading phase, participants consumed a maintenance dose of 5 grams/day for the remainder of the intervention. This dosing strategy has been previously used in RCTs involving older adults and individuals with cancer [66,67]. Participants in the EXPLA group received a CrM placebo supplement (corn-starch maltodextrin; *Bulk Supplements*, Nevada, USA) in the same dose and schedule as EXSUPP. Both supplements had similar flavoring to mask taste and solubility. Participants were provided single use supplement bags weekly and instructed to mix the contents with water and consume once daily at a consistent time, preferably following their exercise session on training days. Supplement adherence was tracked by the number of empty bags returned weekly relative to the number distributed.

### Outcomes

Outcome measures were assessed within 1–2 weeks of initiation and completion of the program. Data collection took place at the University’s Exercise Science Department. Participants were instructed to fast for a minimum of 4 hours prior to each assessment, and every effort was made to schedule pre- and post-intervention testing at the same time of day to minimize diurnal variation.

#### Primary outcome.

The primary outcome of feasibility was evaluated by response rates, defined as the proportion of individuals who responded, relative to all invitations sent, after excluding undeliverable addresses and disconnected phone numbers. Reasons for ineligibility or disinterest and proportional accrual (cases enrolled/cases ascertained) were also tracked to inform trial feasibility. Retention was calculated as the proportion of individuals who returned for post-intervention testing. Adherence was calculated as the proportion of exercise sessions attended (RDI, outlined above) and the proportion of daily supplementation taken. Adverse events were recorded, including a description of the event, its relation to the intervention (not related, unlikely, possibly, probably, definitely), level of seriousness (i.e., non-serious, required hospitalization, resulted in persistent disability, life-threatening, or resulted in death), and intensity (mild, moderate, severe, life-threatening).

Satisfaction with the intervention was assessed at post-testing with an eight-item Likert-type survey with a five-point response scale ranging from strongly disagree to strongly agree. Items assessed the perceived benefit and worth of time, appropriateness to fitness level, helpfulness of in person supervision, satisfaction with virtual session delivery, audio/video quality, cancer specific tailoring by instructors, program simplicity, and willingness to enroll again. Outcomes were summarized as a composite mean score, with higher values indicating greater satisfaction. Four focus groups of 3–7 participants were held at completion of the program to assess participant experiences with the program. Interviews were inductive in nature and allowed participants to elaborate on their experiences and perceptions of the program, as well as how the feasibility and acceptability of the program could be improved. Interviews were recorded, transcribed, and coded for thematic analysis (NVivo Software, QSR International, Burlington, MA, USA).

#### *Secondary outcomes*.

*Body Composition:* Body composition, including whole-body lean soft tissue, appendicular lean soft tissue (ALM), fat mass, and bone mineral content/density, was assessed using DXA (Hologic Horizon A). Standard positioning protocols and quality control procedures were followed for all scans. All scans were performed by a single trained technician with manufacturer quality-control scans performed daily. Instrument precision, determined from the lumbar spine phantom quality-control record across the data-collection period (60 scans) was high, with a coefficient of variation (CV) of 0.26% for BMD. A formal test-retest precision assessment was not conducted within this feasibility trial. We therefore report previously published in vivo precision for the same Hologic Horizon A scanner for the body composition outcomes. Reported CVs for this system are approximately 0.5% for total LST, 1.2% for the ALM index, 0.9% for total fat mass, and 1.2% for whole-body fat percentage.[68,69]

*Muscular Strength***:** Leg extension and chest press strength were assessed using a 1-repetition maximum (1RM) testing protocol. Following a standardized warm-up, participants completed two exercise-specific warm-up attempts of 5–6 repetitions at ~50% of their estimated 1RM, followed by one warm-up attempt of 3 repetitions at ~80% 1RM. Participants then attempted single repetitions at progressively higher loads until the maximal weight that could be lifted with proper technique was achieved. The 1RM test has demonstrated excellent test-retest reliability in older adults across upper- and lower-body exercises (ICC ≥ 0.90) [70,71]. The 1RM value was recorded as the highest successful lift performed with safe technique. Handgrip strength was assessed using a Jamar Plus+ dynamometer (Sammons Preston, Rolyon, Bolingbrook, IL) the reference (gold-standard) device for handgrip strength assessment in clinical populations, which demonstrates excellent test-retest reliability (intraclass correlation coefficients (ICC): 0.93 to 0.98) [72,73].

*Physical Function:* The Short Physical Performance Battery (SPPB) was used to assess lower body function. The SPPB comprised assessments of balance (tandem and semi-tandem stands), gait speed (4-meter walk test in m/s), and chair stand performance (five repeated chair stands). Scores from each component were summed to yield a total score ranging from 0 to 12, with lower scores indicating poorer function and higher scores indicating better function [74]. The SPPB has demonstrated good to excellent test-retest reliability in older adults, (ICC: 0.83 to 0.92) [75,76]. Self-reported physical activity was obtained via the International Physical Activity Questionnaire at baseline, post-intervention, and bi-weekly throughout the intervention to capture physical activity performed outside the intervention [77].

*Health-Related Quality of Life (HRQOL):* Cancer-specific HRQOL was assessed using the European Organization for Research and Treatment of Cancer Quality of Life Questionnaire Core 30 (EORTC-QLQ-C30) [78]. This 30-item validated instrument evaluates five functional domains (physical, role, cognitive, emotional, and social), three symptom domains (fatigue, nausea/vomiting, pain), a global health/quality of life scale, and several single items assessing additional symptoms and financial impact. Items are scored on 4-point Likert scales (1 = “not at all” to 4 = “very much”), with global health status scored on a 7-point scale. The EORTC QLQ-C30 is a validated cancer-specific quality-of-life questionnaire with established internal consistency (Cronbach’s alpha 0.78 to 0.92) [78–81] All scores were linearly transformed to a 0–100 scale, where higher scores on functional and global health scales indicate better functioning/quality of life, and higher scores on symptom scales indicate greater symptom burden. Sarcopenia-related HRQOL was assessed using a sarcopenia-specific questionnaire (SarQoL) [82]. This 55-item, self-administered instrument includes 22 questions grouped into seven domains: physical and mental health, locomotion, body composition, functionality, activities of daily living, leisure activities, and fears. Each item is scored using Likert-type responses, and scores are transformed into domain-specific and overall scores ranging from 0 to 100, with higher scores reflecting better sarcopenia-related quality of life. The SarQoL is a validated sarcopenia-specific quality-of-life questionnaire with high internal consistency (Cronbach’s alpha 0.87 to 0.88) and excellent test-retest reliability (ICC 0.91) [82,83].

### Sample size

As a feasibility trial, the study was not powered to detect efficacy, and a formal power calculation was not performed. The target sample size of n = 40 (20 per group) was instead predetermined on the basis of prior work and established recommendations for pilot and feasibility trials, which prioritize estimating feasibility parameters such as recruitment, retention, and adherence with reasonable precision rather than testing between-group hypotheses [84]. This target was further informed by practical constraints, including the available funding, the associated budget, and the predefined recruitment window, which together bounded the number of participants who could be feasibly recruited, supervised, and assessed within the study period.

### Statistical analysis

To guide the design of future large-scale RCTs, we calculated the mean and standard deviation or proportions and counts of demographics, main outcomes (attendance/adherence and acceptability) and secondary outcomes (muscular strength, body composition, physical function, and quality of life) between the EXSUPP and EXPLA groups as appropriate. In addition, for secondary outcomes we reported mean changes of pre- post- intervention measures and their standard deviations and applied the analyses of covariance (ANCOVA) to examine the association of the post- intervention measures between the EXSUPP and EXPLA groups adjusting for its pre- intervention measures [85,86]. Cohen’s d was calculated as the adjusted mean difference divided by the pooled standard deviation. Missing data were handled using an outcome-specific complete-case approach, whereby participants were included in an analysis only if both baseline and post-intervention values were available for the outcome of interest. No imputation was performed because this was a small pilot feasibility trial, and imputation would have introduced additional modeling assumptions that were unlikely to be reliable given the sample size. Analyses were conducted in R (version 4.4.3; Vienna, Austria). ANCOVA assumptions were evaluated using tests of homogeneity of regression slopes, residual normality, heteroscedasticity, and influential observations. As several of these outcomes exhibited departures from one or more assumptions, sensitivity analyses were conducted using HC3 heteroscedasticity-consistent standard errors and confidence intervals. These analyses were used to assess the stability of the estimated treatment effects. Focus groups were audio recorded, transcribed and thematically coded using NVivo 15.3.2 Qualitative Software (Lumivero, version 15). Analysis proceeded through familiarization with the data, generation of initial codes, construction and review of candidate themes, and definition of final themes. Coding and theme development were conducted by a single researcher; consistent with a reflexive thematic analysis approach, inter-rater reliability was not calculated, as coding was treated as an interpretative rather than a consensus-based process. To support trustworthiness, the coder maintained reflexive notes and an audit trail, and candidate themes were reviewed with the wider research team before being finalized. Analyses were managed in NVivo (version 15.3.2; Lumivero). All deidentified participant-level data, metadata (data dictionary), and analysis code underlying the findings reported in this study are publicly available in the GitHub repository (https://github.com/zhangtiejun310/R21_Resistance-Exercise-Training-and-Creatine-Supplementation)

## Results

**Fig 2:**
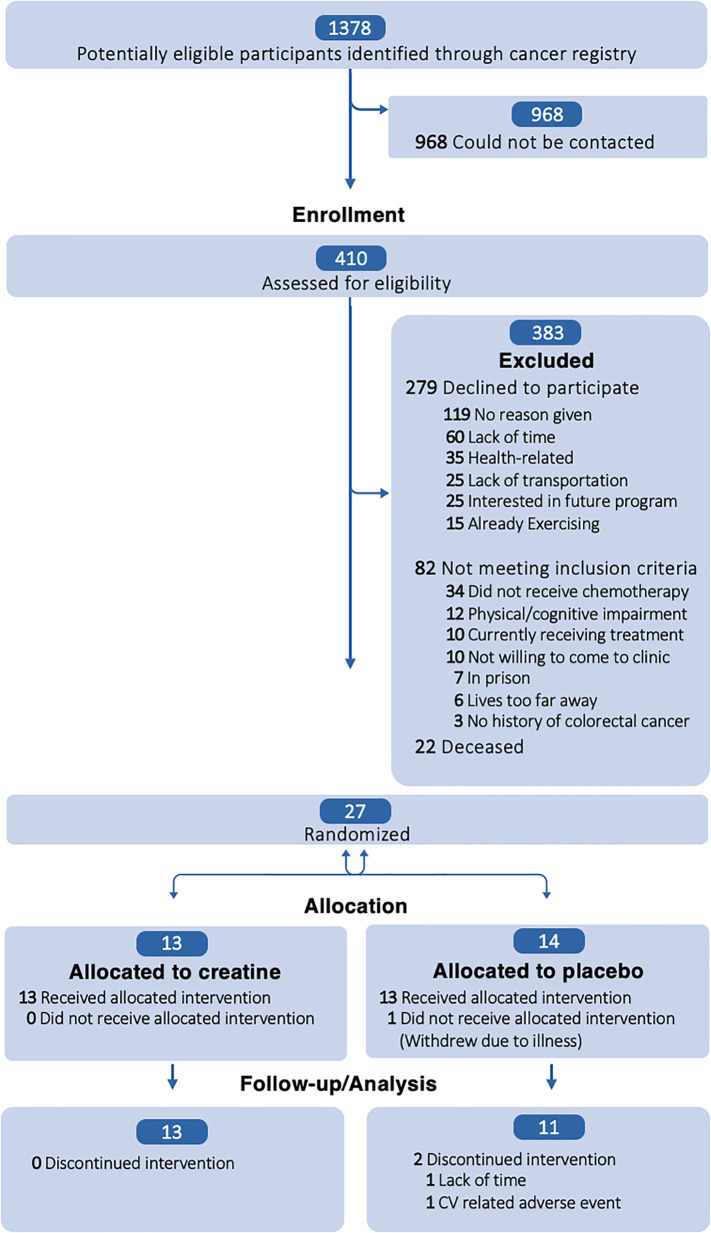
CONSORT diagram.

Participant recruitment occurred between June 2023 and June 2025, with the study completing at the end of the grant funding period. A total of 1,378 individuals were identified as potentially eligible using Prisma Health cancer registries, and invitations were mailed in batches of ~300 over 2 years. We were unable to contact 968 people (i.e., returned mail, or disconnected numbers), and 410 (30%) were assessed for eligibility. Of those, 279 individuals declined to participate, most commonly citing lack of time (n = 60), health-related concerns (n = 35), or lack of transportation (n = 25), while 119 gave no reason. A further 82 individuals did not meet inclusion criteria (common reasons include: no chemotherapy (n = 34); receiving active treatment (n = 10); physical/cognitive impairment (n = 10); distance or unwilling to come to clinic (n = 16), and n = 22 were deceased. Resultantly, 27 individuals (6.6% of those assessed for eligibility) met the inclusion criteria and were enrolled and randomized. Of 27 individuals enrolled, 24 (88.9%) individuals completed the intervention (1 withdrew due to time constraints, 1 removed for an unrelated adverse event and 1 withdrew due to illness) and returned for post-intervention testing. Fig 2 outlines participant recruitment and flow through the study.

### Participant characteristics

The sample included 27 individuals treated for colorectal cancer (EXSUPP; n = 13; EXPLA; n = 14). Participants had a mean age of 64.2 ± 12.7 years, BMI of 29.6 ± 4.8 kg/m^2^, and time since diagnosis of 7.1 ± 6.7 years. Just under half of the sample was male (n = 13, 48%). Most participants identified as White (74%), followed by Black/African American (19%), and Other (7%). Most participants had Stage III colorectal cancer (56%), followed by Stage IV (22%), Stage II (15%), and Stage I (7%). All participants had received prior chemotherapy treatment, and the majority had undergone surgery (93%), with approximately half receiving prior radiation therapy (48%). Select participant demographic and medical characteristics are presented in Table 1.

**Table 1:** Baseline demographic and clinical characteristics of the overall sample and by study group (CrM vs placebo).

Characteristic	Total (n = 27)	CrM (n = 13)	Placebo (n = 14)
**Age**, y (mean ± SD)	64 ± 13	61 ± 14	67 ± 10
**Sex**, n (%)			
Male	13 (48%)	8 (61%)	5 (36%)
Female	14 (52%)	5 (39%)	9 (64%)
**BMI**, kg/m^2^ (mean ± SD)	29.6 ± 4.8	30.5 ± 5.2	28.7 ± 4.5
**Race**, n (%)			
White	20 (74%)	10 (77%)	10 (71%)
Black/African American	5 (19%)	2 (15%)	3 (21%)
Other	2 (7%)	1 (8%)	1 (7%)
**Ethnicity**, n (%)			
Not Hispanic or Latino	26 (96%)	13 (100%)	13 (93%)
**Education**, n (%)			
Some college or less	12 (44%)	5 (38%)	7 (50%)
Bachelor’s or higher	15 (56%)	8 (62%)	7 (50%)
**Comorbidities**, n (%)			
Cardiovascular Disease	6 (22%)	3 (23%)	3 (21%)
Diabetes	4 (15%)	2 (15%)	2 (14%)
Hypertension	8 (30%)	4 (31%)	4 (29%)
Kidney disease	2 (7%)	1 (8%)	1 (7%)
**Colorectal cancer stage**, n (%)			
Stage I	2 (7%)	0 (0%)	2 (14%)
Stage II	4 (15%)	1 (8%)	3 (21%)
Stage III	15 (56%)	9 (69%)	6 (43%)
Stage IV	6 (22%)	3 (23%)	3 (21%)
**Time since diagnosis,** y (mean ± SD)	7.1 ± 6.7	8.5 ± 6.8	5.7 ± 6.4
**Time since chemotherapy,** y (mean ± SD)	5.0 ± 5.5	6.6 ± 7.2	3.3 ± 2.1
**Additional treatments**, n (%)			
Surgery	25 (93%)	12 (92%)	13 (93%)
Radiation	13 (48%)	6 (46%)	7 (50%)

### Attendance/Adherence

Participants completed an average of 25.4 ± 8 of the 30 planned sessions, corresponding to an overall RDI of 91.5% ± 12.2%. Attendance was slightly lower for in-person sessions (16.7 ± 3.1 of 20 planned; 83.4% ± 15.4% compared to virtual sessions (10.8 ± 2.7 of 10 planned; 108.0% ± 27.1%). The mean completed training load was 48,558 ± 22,110 kg, representing 90.7% ± 12.3% of the planned training load (52,518 ± 21,993 kg). The RDI for both supplement and Placebo were 95 and 94% respectively. Adherence, RDI, and reasons for missed and modified sessions are presented in S2 File.

### Acceptability

Participants reported high acceptability of the overall intervention (4.6 ± 1.1 out of 5) and willingness to enroll in a similar program in the future was equally high (4.5 ± 1.2). Participants highly rated the in-person supervision (4.6 ± 1.3) to tailor exercises to cancer-related concerns (4.4 ± 1.2) and level of fitness (4.5 ± 1.1). The satisfaction with the virtual component was also high, for both the teleconferencing aspect (4.1 ± 1.2), and the quality of audio and video for virtual sessions (4.2 ± 1.1). Individual components of the acceptability questionnaire are presented in S2 File.

Focus group discussions revealed that participants perceived the program as highly supportive and confidence-building. Across all groups, individualized one-on-one supervision was described as central to success, particularly in mitigating fear of injury and facilitating progressive challenge. Participants emphasized that structured accountability and trainer guidance enabled engagement beyond what they believed they would have achieved independently. Beyond physical improvements, participants described broader psychosocial benefits, including increased confidence, restoration of normalcy, and motivation to engage in other health behaviors. Negative feedback was minimal and largely limited to transient supplement-related discomfort. While most participants reported few barriers to participation, those that emerged included initial fear of injury and logistical constraints, suggesting that supervised delivery may have mitigated commonly anticipated obstacles. Recommendations for future programs centered on earlier referral, integration with oncology teams, additional staffing, and incorporation of nutrition education.

### Adverse events

A total of five adverse events were reported in the EXSUPP group (n = 13) and four in the EXPLA group (n = 14). Most adverse events were classified as mild in severity. Notably, three individuals in the EXSUPP group noted mild GI symptoms during the 7-day loading phase of CrM. In these cases, participants were instructed to reduce their CrM intake to 5 grams/day instead of the prescribed 20 grams/day, after which symptoms resolved. One serious adverse event occurred with a participant in the EXPLA group. In the 6^th^ week of the intervention, the participant experienced a cardiac event outside of the exercise session and was hospitalized. The participant was withdrawn from the study, an adverse event form was completed and filed with the University IRB, and the event was deemed to be unrelated to the intervention.

### Secondary outcomes

#### Muscular strength, body composition, and physical function.

Both EXPLA and EXSUPP groups experienced changes in muscular strength, body composition (LST, ALM and BF%) and physical function. Detailed outcome measures for all variables are reported in Table 2. After adjustment for baseline values, the estimated between-group differences (CrM minus placebo) generally favored CrM for most outcomes, with the exception of visceral fat, although confidence intervals were wide. For example, for muscular strength, the adjusted mean differences were 0.06 kg for grip strength (p = 0.97; 95% CI −3.28 to 3.40), 0.44 kg for chest press (p = 0.83; 95% CI −3.71 to 4.59), and 2.04 kg for leg extension (p = 0.57; 95% CI −5.45 to 9.58). Cohen’s *d* values indicated trivial to small effects (d = 0.02–0.26).

**Table 2:** Muscular strength, body composition and physical function.

	Baseline				Post-Intervention				Change from Baseline		Adjusted Group Difference^a^		
Outcome	EXPLA		EXSUPP		EXPLA		EXSUPP		EXPLA	EXSUPP			
	n	mean ± SD	n	mean ± SD	n	mean ± SD	n	mean ± SD	mean ± SD	mean ± SD	Mean (95% CI)	P value^b^	Cohen’s D
**Muscular Strength**													
Grip Strength (kg)	14	30.13 ± 8.08	13	38.73 ± 9.99	11	32.37 ± 7.39	13	38.90 ± 9.79	0.62 ± 2.70	0.17 ± 4.19	0.06 (−3.28, 3.40)	0.97	0.02
Chest Press (kg)	14	32.40 ± 14.90	13	49.37 ± 22.15	11	43.71 ± 15.28	13	57.92 ± 24.63	6.80 ± 3.80	8.55 ± 5.64	0.44 (−3.71, 4.59)	0.83	0.09
Leg Extension (kg)	14	34.18 ± 16.41	11	48.45 ± 16.39	11	51.34 ± 13.04	11	61.44 ± 17.58	11.75 ± 7.23	12.99 ± 8.61	2.07 (−5.45, 9.59)	0.57	0.26
**Body Composition**													
LST (kg)	14	49.91 ± 6.66	13	59.04 ± 11.84	11	48.91 ± 4.47	13	60.91 ± 12.68	0.58 ± 2.08	1.87 ± 2.91	0.97 (−1.63, 3.57)	0.45	0.37
ALM (kg)	14	20.90 ± 3.19	13	25.17 ± 6.50	11	20.56 ± 2.22	13	26.77 ± 7.39	0.56 ± 1.02	1.59 ± 1.36	0.48 (−0.60, 1.55)	0.37	0.43
ALM/height^2^ (kg/m2)	14	7.43 ± 1.11	13	8.30 ± 1.82	11	7.37 ± 0.63	13	8.76 ± 2.02	0.18 ± 0.36	0.46 ± 0.42	0.23 (−0.14, 0.59)	0.21	0.57
Fat Mass (kg)	14	29.06 ± 8.66	13	31.37 ± 9.10	11	25.60 ± 7.62	13	30.33 ± 8.32	−0.67 ± 1.94	−1.05 ± 2.45	0.02 (−1.93, 1.97)	0.98	0.01
Visceral Fat (kg)	14	0.63 ± 0.35	13	0.67 ± 0.28	11	0.61 ± 0.33	13	0.60 ± 0.20	−0.02 ± 0.10	−0.06 ± 0.16	−0.04 (−0.14, 0.06)	0.46	−0.31
Body Fat %	14	35.21 ± 7.14	13	33.58 ± 5.56	11	32.89 ± 7.88	13	32.28 ± 6.74	−0.84 ± 2.44	−1.29 ± 2.35	−0.46 (−2.54, 1.63)	0.65	−0.19
BMD (g/cm^2^)	14	1.09 ± 0.12	13	1.11 ± 0.11	11	1.11 ± 0.14	13	1.10 ± 0.11	0.01 ± 0.01	−0.01 ± 0.03	−0.02 (−0.04, 0.00)	0.11	−0.68
**Physical Function**													
SPPB (AU)	14	10.57 ± 2.17	13	11.08 ± 1.66	11	11.55 ± 0.93	13	11.92 ± 0.28	0.27 ± 0.47	0.85 ± 1.52	0.43 (−0.05, 0.91)	0.08	0.77

^a^Adjusted Group Difference represents the estimated difference between the CrM and placebo groups (CrM – Placebo) after adjustment for baseline values using ANCOVA; ^b^p values correspond to tests of the adjusted between-group difference. ALM: Appendicular lean soft tissue; LST: Lean soft tissue; BMD: Bone Mineral Density; AU: Arbitrary Units; SPPB: Short Physical Performance Battery.

For body composition outcomes, the adjusted mean differences (CrM minus placebo) were small and non-significant, including 0.97 kg for lean soft tissue (p = 0.45; 95% CI −1.63 to 3.57), 0.48 kg for ALM (p = 0.37; 95% CI −0.60 to 1.55), and 0.23 kg/m^2^ for height-adjusted ALM (p = 0.21; 95% CI −0.14 to 0.59). Adjusted differences were 0.02 kg for total fat mass (p = 0.98; 95% CI −1.93 to 1.97), −0.04 kg for visceral fat (p = 0.46; 95% CI −0.14, 0.06), −0.46% for body fat percentage (p = 0.65; 95% CI −2.54 to 1.63), and −0.02 g/cm² for bone mineral density (p = 0.11; 95% CI −0.04 to 0.00). Effect sizes ranged from trivial to small (d = −0.68 to 0.01). For physical function, EXSUPP group averaged a 0.43-point higher SPPB score than the EXPLA group, corresponding to a moderate effect size (d = 0.77), though the difference was not statistically significant (p = 0.08).

### Quality of life

Quality of life outcomes did not differ significantly between the EXPLA and EXSUPP groups. Most symptom scales including fatigue, nausea/vomiting, pain, dyspnea, sleep disturbance, appetite loss, constipation, and diarrhea also showed no significant-group differences (all p > 0.10). The only nominally significant finding among the quality-of-life outcomes was observed for the Financial Difficulties subscale, where the EXSUPP group reported lower scores compared with EXPLA (−13.52; p = 0.04; 95% CI −26.24 to −0.80; d = −0.91). Adjusted mean differences were −6.86 points for overall functioning (p = 0.14; 95% CI −16.18 to 2.45), −0.16 points for physical functioning (p = 0.95; 95% CI −5.54 to 5.22), 2.13 points for role functioning (p = 0.49; 95% CI −4.20 to 8.47), −5.73 points for emotional functioning (p = 0.10; 95% CI −12.67 to 1.20), 3.89 points for cognitive functioning (p = 0.42; 95% CI −5.91 to 13.68), and −10.70 points for social functioning (p = 0.15; 95% CI −25.60 to 4.20). Effect sizes ranged from small to moderate (d = 0.77 to 0.66), none of which were statistically significant (S2 File). For the SarQoL measure, there was no significant difference between groups (−5.06 points p = 0.12; 95% CI −11.53 to 1.41). Across all QoL models, baseline scores were consistent and strong predictors of follow-up outcomes (p < 0.01).

Model diagnostics indicated that the assumptions of the ANCOVA models were satisfied for the strength and body composition outcomes. Several secondary outcomes, particularly quality-of-life measures, exhibited evidence of non-normal residuals, heteroscedasticity, or influential observations. Such departures are not unexpected in a pilot trial with a small sample size and bounded patient-reported outcome measures. To evaluate the robustness of the findings, we conducted sensitivity analyses using HC3 heteroscedasticity-consistent standard errors, confidence intervals, and p values. These analyses yielded effect estimates of similar magnitude and direction to those obtained from the primary ANCOVA models and did not materially alter the interpretation of the results. Confidence intervals remained wide, and no between-group comparison was statistically significant at the 0.05 level (S2 File). These findings suggest that the overall conclusions were not substantially influenced by violations of model assumptions and reinforce the exploratory nature of the secondary outcome analyses. To facilitate interpretation of individual responses, Cumming-style estimation plots were added to display baseline-to-post-intervention changes by randomized group (S5 File).

## Discussion

The primary aim of this trial was to examine the feasibility, safety and acceptability of a 10-week RCT comparing RET with or without CrM in individuals previously treated for colorectal cancer. A total of 6.6% (n = 27) of individuals screened for eligibility were recruited to participate. Retention rate was 88.9% and the RDI for the RET and supplementation was 91.5% ± 12.2% (EXSUPP 94.1 ± 7.8, EXPLA 88.6 ± 15.5) and 94% (SUPP 95%, PLA 94%), respectively. Participants highly rated the overall intervention and perceived it to be beneficial (4.6 ± 1.1 out of 5) and focus group discussions were overwhelmingly positive. Further, no serious exercise/supplement related adverse events were recorded. The high retention rates and acceptability ratings, the strong RDI for both exercise and supplementation, and the lack of adverse events attributed to the intervention, all met/exceeded a priori indicators of feasibility and support the feasibility of the intervention delivery, once individuals are enrolled. However, we failed to reach the intended target of n = 40 individuals recruited across 24-months, which warrants discussion around the feasibility of recruiting this specific population [87]. Further, several changes were made to the protocol to enhance accrual, which are also worthy of conversation in the context of feasibility.

The recruitment of n = 27 for this study fell below the target of n = 40 across 24 months, and the recruitment rate of 6.6% raises concerns regarding the ability to successfully recruit individuals with colorectal cancer treated with chemotherapy using the strategies employed in this trial. Our recruitment rate of ~6.6% also falls well below the median of ~31.28% that has been previously reported in exercise oncology trials of exercise and colorectal cancer [88]. The primary recruitment strategy for this particular trial was through the use of cancer registries. The registry covered a 10-year retrospective period, and we were unable to contact 70.2% (n = 968) of potentially eligible individuals. Inherently, using registry data for successful recruitment is contingent on the maintenance/quality of the registry itself. Further, whilst registries are valuable for identifying large pools of potentially eligible individuals, other recruitment strategies such as in-clinic recruitment, or direct referrals from treating medical professionals could be explored in future work as a means to enhance recruitment strategies [87–90].

Despite recruitment challenges, the high retention rate (88.9%), the high RDI for the RET (91.5%), strong satisfaction and lack of serious adverse events all support the safety, feasibility and acceptability of the intervention, once individuals are recruited. Our RDI compares favorably and exceeds those of other trials in exercise oncology [44,63]. Notably, every effort was made to maintain a 1:1 participant-to-instructor ratio for intervention delivery. Though this 1:1 format and individualized care may likely have influenced our high RDI, the scalability of this approach from a cost and logistics perspective, warrants consideration in the design of future large RCTs. The high RDI for CrM supplementation (94%) was comparable to that reported in previous randomized controlled trials of CrM supplementation and surpassed the a priori feasibility criterion of ≥75% [34,91–95]. Further, no participants withdrew due to supplement related burden, and no serious supplement related adverse events were observed. Mild gastrointestinal symptoms were reported by three participants during the initial 7-day loading phase (20 grams/day); however, these symptoms resolved following transition to the 5 grams/day maintenance dose, with no further supplementation related issues reported. These findings suggest that CrM supplementation is feasible in this population, though future trials in oncology may consider omitting a loading phase or employing a more gradual titration strategy to further enhance tolerability, without compromising adherence. Contemporary perspectives on feasibility research emphasize “tiered” progression criteria, that informs whether to proceed to a full trial as is (“Go”), with changes (“Amend”), or to not proceed (“STOP”) rather than traditional binary thresholds [96–98]. The tiering structure provides an opportunity to evaluate which components of the trial design are strong, which are worth pursuing with modifications, and which elements should be reconsidered. In this regard, despite recruitment challenges, the high retention rate (88.9%), the high RDI for the RET (91.5%) and supplementation (94%), respectively, strong satisfaction and lack of serious adverse events all support the safety, feasibility and acceptability of the intervention, once individuals are recruited. Though not a part of our a priori criteria for success, applying this framework to our findings supports the feasibility of delivering a 10-week RET intervention with CrM supplementation to individuals treated for cancer.

A secondary aim of the study was to assess changes in body composition, muscular strength, physical function, and quality of life. These secondary outcomes were exploratory and hypothesis-generating as the trial was not designed or powered to evaluate efficacy. Further, in the absence of a non-exercise control group, the observed changes cannot be attributed to RET specifically. We were able to obtain these measures in nearly all participants, demonstrating that the assessment battery was feasible to administer. Adjusted analyses showed no meaningful differences between groups for LST, strength, or physical function, and wide variability across participants limits interpretation. Both groups showed modest within-group changes in muscular strength and SPPB scores over the intervention period. These within-group changes are directionally consistent with the broader literature on RET in individuals with cancer [99–104], and the lack of additive effects of CrM are similar to our previous research in prostate cancer [44]. The changes observed across both groups are encouraging given the well-documented declines in these outcomes following chemotherapy [5–9], and support the rationale for a future adequately powered, controlled trial to test whether RET counteracts treatment-related muscle decline and its associated mortality risk [10–12]. As this feasibility study was not powered for between-group comparisons, null effects should be interpreted cautiously rather than as evidence of no benefit of CrM supplementation.

Our study has several limitations worthy of note. The small sample size and narrow sample characteristics limit the generalizability of our findings. The low recruitment rate (6.6%) and the relatively high baseline physical function of those enrolled (SPPB approximately 10–11 of 12) raise the possibility of selection or volunteer bias, whereby those who enrolled may have been more motivated and physically capable than the broader colorectal cancer population. Our findings may therefore not generalize to more vulnerable individuals, including those who are sarcopenic, frail, or more recently treated. CrM supplementation may increase total body water, which may influence lean soft tissue estimates from DXA. Increases in intracellular fluid would be indicative of creatine uptake into the muscle, which would be supportive of muscle tissue hypertrophy changes with exercise. However, without a measure of body fluid (via multifrequency bioelectrical impedance), we cannot be confident what changes in body composition are from changes in total body water, versus true tissue changes [34,105]. In addition, the 10-week duration may be insufficient to detect hypertrophic adaptations, which typically emerge over longer training periods; future trials should be of longer duration, powered for between-group comparisons, and should incorporate a multi-compartment (four-compartment) body composition model that includes total body water measured by isotope dilution (e.g., deuterium dilution or doubly labeled water), to distinguish CrM-associated fluid shifts from true tissue accretion. Finally, although the hybrid delivery model was feasible and well adhered to, the home-based equipment used for remote sessions may constrain progressive loading relative to in-clinic machines, which warrants consideration when designing the training stimulus for a future efficacy trial.

### Considerations for progressing to a future definitive trial

The high retention, strong RDI, positive acceptability, and absence of serious adverse events support advancing the RET and CrM intervention protocol towards a future definitive trial. However, registry-based recruitment produced an accrual rate well below comparable trials and a sample less representative of those most likely to benefit. A definitive trial should therefore consider alternative methods of recruitment, such as in-clinic identification and direct referral from treating clinicians, ideally engaging patients during or shortly after treatment. Further, more dedicated strategies are needed to reach individuals with low muscle mass or physical function, who are most vulnerable to treatment-related muscle loss. Such a trial should also be adequately powered for between-group comparisons with an appropriate non-exercise comparator to isolate the contribution of RET and be of longer duration to allow structural adaptations to emerge. Refinement of the CrM dosing and progressive-loading strategy across in-person and remote sessions should also be considered.

## Conclusions

The hybrid delivery of a RET intervention with CrM appears to be feasible and acceptable. The lack of adverse events supports the safety of the intervention. However, the low recruitment rate using cancer registries is notable, and future work could look to include complementary strategies such as in-clinic recruitment or direct referral from medical providers to enhance recruitment efforts. Ultimately, planning for a larger trial should weigh these feasibility and acceptability findings against implementation burden, including staff time, space and equipment needs, and participant time/travel, that will influence accessibility and scalability.

## Supporting information

S1 File Consort checklist. (DOCX)

S2 File Supplementary details and outcomes. (DOCX)

S3 File Approved protocol. (DOCX)

S4 File De-identified training logs. (XLSX)

S5 File Estimation plots. (TIFF)
