# Supplementary material for: Examining the feasibility and preliminary effects of resistance exercise training and creatine supplementation in individuals treated for colorectal cancer
Source: PLoS One. 2026 Jul 15;21(7):e0353630. doi: 10.1371/journal.pone.0353630 (PMC13372120; doi:10.1371/journal.pone.0353630)
Supplement: S2 File — (DOCX) [file pone.0353630.s002.docx]

| S2 Table 1. Changes to the study protocol. | | | |
| --- | --- | --- | --- |
| **Original Protocol** | **Change Made** | **When** | **Rationale** |
| *“Individuals interested in participating will complete a pre-screening questionnaire (SARC-F) to determine suitability. If phone screening is successful, individuals will complete an objective diagnosis of sarcopenia (via DXA and physical function assessment) to determine eligibility.”* | Removed sarcopenia as an eligibility criterion. | After first person recruited | After 12 months of recruitment, only one interested individual screened positive for sarcopenia using SARC-F, and this individual did not meet sarcopenia criteria on follow-up objective assessment. Given the extremely limited accrual, requiring sarcopenia substantially hindered feasibility. Removal of this criterion allowed broader eligibility. |
| *“Individuals ≥12 months post-treatment for colorectal cancer will be included.”* | Added prior exposure to chemotherapy as an inclusion criterion. | After first person recruited | With sarcopenia removed, exposure to chemotherapy was added as a biologically relevant inclusion criterion. Chemotherapy is associated with adverse effects on skeletal muscle mass, quality, and function. This criterion maintained a focus on a vulnerable population, while improving recruitment feasibility. |
| *“Participants will be randomized in a ratio of 1:1 to either EXSUPP or EXPLA using a computer-generated program. Participants will be stratified according to age, sex and race.”* | Participants were randomized without stratification. | After first person recruited | Recruitment challenges (27 enrolled of 40 planned) limited the feasibility of stratified randomization. Instead, an a priori randomization sequence developed by an independent statistician was followed to preserve allocation concealment, albeit without stratification by demographic variables. |

| Table 1. Continued… | | | |
| --- | --- | --- | --- |
| **Original Protocol** | **Change Made** | **When** | **Rationale** |
| *“The intervention will be preceded by a 2-week familiarization phase, including 4 visits to the clinic across two weeks.”* | Familiarization phase was removed, and participants began the 10-week intervention with 3 sessions in week 1. | Before recruitment | The familiarization period was integrated into the first week of training. This streamlined design reduced participant burden, improved feasibility, and still ensured safe, individualized progression, consistent with clinical exercise oncology practice. |
| *“Participants in the supplement group will receive creatine monohydrate at a dose of 0.10g/kg/day.”* | Participants in the supplement group received 5 g/day, irrespective of body weight. | Before recruitment | The fixed dose reflected real-world application, as most consumers follow label directions (5 g/day). This approach enhances external validity and translational relevance while remaining within the range of doses shown to be effective and safe in prior clinical and athletic research. |
| *“Leg press and chest press strength will be assessed using a 1-repetition maximum testing protocol.”* | Leg press 1RM testing was replaced with 1RM leg extension. | Before recruitment | A leg press machine was not available at the study site. Leg extension 1RM was selected as a widely used alternative measure of lower body strength that could be administered consistently across participants. |

| Table 1. Continued… | | | |
| --- | --- | --- | --- |
| **Original Protocol** | **Change Made** | **When** | **Rationale** |
| *“To accommodate different schedules, we will provide 3 options for exercise sessions: early morning (~6:30-7:30am), mid-day (~12-1pm), and early evening (~5:30-6:30pm) at the lab each weekday.”* | The 6:30am session was removed and a 6:30–7:30pm session was added. | During recruitment period, but not before participants were recruited | No participants expressed interest in early morning sessions, while evening availability was requested. Adjusting session times improved feasibility, adherence, and participant-centeredness without altering intervention fidelity. |
| *“All exercise sessions will occur in-person supervised by members of the study team specifically trained in exercise oncology. Participants assigned to each arm will attend a total of 30 supervised exercise sessions for 10 weeks (thrice weekly, with a minimum 48 hours rest between sessions).”* | The third weekly session was delivered virtually (hybrid model). | Before recruitment | To reduce travel burden and enhance accessibility, the Friday session was conducted virtually under supervision (Monday/Wednesday remained in-person). This hybrid approach reflects emerging models in exercise oncology, potentially improving reach, adherence, and translational scalability while maintaining safety oversight. |

S2 Table 2. Planned RET progression across the 10-week intervention.

| **Week(s)** | **Sets per exercise** | **Repetitions** | **Target effort (RIR*)** |
| --- | --- | --- | --- |
| Week 1 | 2 | 12 | 3-4 |
| Week 2-5 | 3 | 12 | 2-3 |
| Week 6-8 | 3 | 10 | 2-3 |
| Week 9-10 | 3 | 8 | 2-3 |

*RIR = repetitions in reserve.

S2 Table 3. RDI and reasons for missed/modified sessions.

|  | **Planned** | **Total Completed**  **(mean** ± **SD)** | | | **CrM Completed**  **(mean** ± **SD)** | | **ExPla Completed**  **(mean** ± **SD)** | | **Total**  **RDI%**  **(mean** ± **SD)** | | | **CrM**  **RDI%**  **(mean** ± **SD)** | | **ExPla**  **RDI%**  **(mean** ± **SD)** |
| --- | --- | --- | --- | --- | --- | --- | --- | --- | --- | --- | --- | --- | --- | --- |
| Total sessions (n) | 30 | 27.4 ± 3.7 | | | 28.2 ± 2.4 | | 26.6 ± 4.7 | | 91.5 ± 12.2 | | | 94.1 ± 7.8 | | 88.6 ± 15.5 |
| In-person (n) | 20 | 16.7 ± 3.1 | | | 18.0 ± 1.9 | | 15.3 ± 3.5 | | 83.4 ± 15.4 | | | 90.0 ± 9.6 | | 76.3 ± 17.6 |
| Virtual (n) | 10 | 10.8 ± 2.7 | | | 10.2 ± 2.6 | | 11.3 ± 2.8 | | 108.0 ± 27.1 | | | 102.3 ± 26.2 | | 113.3 ± 28.1 |
| Training volume (kg) | 52,518 ± 21,993 | 48,558 ± 22,110 | | | 57,331 ± 20,036 | | 39,053 ± 20,956 | | 90.7 ± 12.3 | | | 92.1 ± 8.4 | | 89.2 ± 15.7 |
| Supplementation (doses) | 91 | 85.9 ± 9.1 | | | 86.4 ± 9.6 | | 85.3 ± 8.9 | | 94.3 ± 10.0 | | | 95.0 ± 10.5 | | 94.0 ± 9.8 |
|  | **No. of participants (n)** | | | **Percentage (%)** | | | | **No. of sessions (n)** | | | **Percentage (%)** | | | |
| **Missed sessions** |  | |  | | |  | |  | |  | | |  | |
| Vacation | 5 | | | 18.5 | | | | 8 | | | 1.2 | | | |
| Fatigue | 4 | | | 14.8 | | | | 6 | | | 0.8 | | | |
| Injury | 4 | | | 14.8 | | | | 3 | | | 0.4 | | | |
| Nausea | 1 | | | 3.7 | | | | 3 | | | 0.4 | | | |
| Illness | 6 | | | 22.0 | | | | 9 | | | 1.3 | | | |
| Conflicting appointments | 4 | | | 14.8 | | | | 8 | | | 1.2 | | | |
| Personal | 8 | | | 29.6 | | | | 13 | | | 1.9 | | | |
| Other | 16 | | | 59.3 | | | | 24 | | | 3.5 | | | |
| **Modification Type** |  | |  | | |  | |  | |  | | |  | |
| Sets | 6 | | | 22.2 | | | | 7 | | | 1.0 | | | |
| Reps | 7 | | | 25.9 | | | | 18 | | | 2.6 | | | |
| Weight | 13 | | | 48.1 | | | | 28 | | | 4.1 | | | |
| Exercise Selection | 6 | | | 22.2 | | | | 9 | | | 3.3 | | | |
| Exercise removal | 4 | | | 14.8 | | | | 3 | | | 0.4 | | | |
| **Modification Reason** |  | |  | | |  | |  | |  | | |  | |
| Fatigue | 7 | | | 25.9 | | | | 8 | | | 1.2 | | | |
| Nausea | 1 | | | 3.7 | | | | 1 | | | 0.1 | | | |
| Pain | 5 | | | 18.5 | | | | 7 | | | 1.0 | | | |
| Dizziness | 2 | | | 7.4 | | | | 1 | | | 0.1 | | | |
| Gastrointestinal related | 1 | | | 3.7 | | | | 1 | | | 0.1 | | | |
| Other | 10 | | | 37.0 | | | | 17 | | | 2.5 | | | |

**S2 Table 4. Post-intervention participant satisfaction (n = 24)**

| **Survey Item** | **Mean ± SD**  *(1 = strongly disagree, 5 = strongly agree)* |
| --- | --- |
| I felt that the overall intervention was beneficial and worth my time. | 4.6 ± 1.1 |
| I felt like the exercises were tailored to my level of fitness. | 4.5 ± 1.1 |
| I found having in-person supervision at the exercise clinic was helpful. | 4.6 ± 1.3 |
| I am fully satisfied with the teleconferencing for virtual exercise sessions. | 4.1 ± 1.2 |
| I found the quality of audio and video for teleconferencing for virtual exercise sessions to be high. | 4.2 ± 1.1 |
| I felt like the exercise instructors were able to tailor the exercises to any cancer-related concerns I had. | 4.4 ± 1.2 |
| I felt the exercise program was simple and easy to follow (i.e. not overcomplicated). | 4.5 ± 1.1 |
| I would sign up for a similar program in the future. | 4.5 ± 1.2 |

| Table 5. Quality of Life ANCOVA Results | | | | | | | | | | | | | |
| --- | --- | --- | --- | --- | --- | --- | --- | --- | --- | --- | --- | --- | --- |
|  | Baseline | | | | Post-Intervention | | | | Change from Baseline | | Adjusted Group Difference^a^ |  |  |
| Outcome | Placebo | | Creatine | | Placebo | | Creatine | | Placebo | Creatine |  |  |  |
|  | n | mean ± SD | n | mean ± SD | n | mean ± SD | n | mean ± SD | mean ± SD | mean ± SD | Mean 95% CI | P value^b^ | Cohen’s D |
| **Quality of Life** |  |  |  |  |  |  |  |  |  |  |  |  |  |
| Global Health Status | 14 | 72.02 ± 22.79 | 13 | 74.36 ± 12.01 | 11 | 84.85 ± 17.00 | 13 | 78.85 ± 10.55 | 12.12 ± 13.62 | 4.49 ± 13.01 | -6.86 (-16.18, 2.45) | 0.14 | -0.63 |
| **Functional Scales** |  |  |  |  |  |  |  |  |  |  |  |  |  |
| Physical Functioning | 14 | 87.62 ± 17.51 | 13 | 91.28 ± 9.96 | 11 | 92.73 ± 13.81 | 13 | 93.85 ± 6.92 | 3.64 ± 10.48 | 2.56 ± 6.96 | -0.16 (-5.54, 5.22) | 0.95 | -0.02 |
| Role Functioning | 14 | 84.52 ± 28.09 | 13 | 94.87 ± 10.51 | 11 | 92.42 ± 17.26 | 13 | 100.00 ± 0.00 | 10.61 ± 18.67 | 5.13 ± 10.51 | 2.13 (-4.20, 8.47) | 0.49 | 0.30 |
| Emotional Functioning | 14 | 73.81 ± 26.12 | 13 | 91.67 ± 10.21 | 11 | 90.15 ± 13.34 | 13 | 91.67 ± 9.62 | 14.39 ± 18.29 | 0.00 ± 4.81 | -5.73 (-12.67, 1.20) | 0.10 | -0.77 |
| Cognitive Functioning | 14 | 82.14 ± 22.13 | 13 | 91.03 ± 11.00 | 11 | 84.85 ± 13.85 | 13 | 92.31 ± 11.00 | 4.55 ± 18.40 | 1.28 ± 14.37 | 3.89 (-5.91, 13.68 | 0.42 | 0.35 |
| Social Functioning | 14 | 72.62 ± 27.43 | 13 | 93.59 ± 10.84 | 11 | 87.88 ± 22.47 | 13 | 88.46 ± 20.84 | 10.61 ± 13.48 | -5.13 ± 19.70 | -10.70 (-25.60, 4.20) | 0.15 | -0.66 |
| **Symptom Scales** |  |  |  |  |  |  |  |  |  |  |  |  |  |
| Fatigue | 14 | 32.54 ± 25.21 | 13 | 16.24 ± 10.75 | 11 | 16.16 ± 19.48 | 13 | 12.82 ± 12.71 | -18.18 ± 18.77 | -3.42 ± 15.96 | 4.89 (-7.68, 17.46) | 0.43 | 0.36 |
| Nausea/Vomiting | 14 | 10.71 ± 27.43 | 13 | 0.00 ± 0.00 | 11 | 9.09 ± 30.15 | 13 | 1.28 ± 4.62 | -3.03 ± 10.05 | 1.28 ± 4.62 | 3.41 (-3.36, 10.18) | 0.31 | 0.45 |
| Pain | 14 | 25.00 ± 31.86 | 13 | 10.26 ± 12.80 | 11 | 12.12 ± 15.08 | 13 | 11.54 ± 14.25 | -9.09 ± 30.15 | 1.28 ± 15.90 | 1.17 (-11.6, 13.94) | 0.85 | 0.08 |
| Dyspnea | 14 | 14.29 ± 25.20 | 13 | 2.56 ± 9.25 | 11 | 12.12 ± 22.47 | 13 | 10.26 ± 16.01 | -3.03 ± 17.98 | 7.69 ± 14.62 | 6.15 (-7.25, 19.56) | 0.35 | 0.41 |
| Insomnia | 14 | 40.48 ± 37.39 | 13 | 17.95 ± 32.25 | 11 | 21.21 ± 26.97 | 13 | 10.26 ± 16.01 | -12.12 ± 26.97 | -7.69 ± 30.89 | -5.76 (-22.19, 10.67) | 0.47 | -0.31 |
| Appetite Loss | 14 | 9.52 ± 27.51 | 13 | 5.13 ± 12.52 | 11 | 9.09 ± 30.15 | 13 | 5.13 ± 12.52 | -3.03 ± 10.05 | 0.00 ± 13.61 | 1.91 (-8.30, 12.12) | 0.70 | 0.16 |
| Constipation | 14 | 14.29 ± 28.39 | 13 | 12.82 ± 16.88 | 11 | 9.09 ± 15.57 | 13 | 2.56 ± 9.25 | 0.00 ± 14.91 | -10.26 ± 21.01 | -7.09 (-17.89, 3.69) | 0.19 | -0.56 |
| Diarrhea | 14 | 28.57 ± 34.24 | 13 | 15.38 ± 22.01 | 11 | 18.18 ± 34.52 | 13 | 20.51 ± 32.03 | -15.15 ± 22.92 | 5.13 ± 26.69 | 16.19 (-5.88, 38.26) | 0.14 | 0.66 |
| Financial Difficulties | 14 | 11.90 ± 30.96 | 13 | 15.38 ± 22.01 | 11 | 12.12 ± 30.81 | 13 | 2.56 ± 9.25 | 3.03 ± 10.05 | -12.82 ± 21.68 | -13.52 (-26.24, -0.80) | 0.04 | -0.91 |
| SarQoL | 14 | 70.34 ± 15.32 | 13 | 76.04 ± 7.13 | 11 | 85.32 ± 14.56 | 13 | 82.92 ± 9.07 | 12.57 ± 8.88 | 6.89 ± 6.49 | -5.06 (-11.53, 1.41) | 0.12 | -0.67 |
| ^a^Adjusted Group Difference represents the estimated difference between the creatine and placebo groups (creatine - placebo) after adjustment for baseline values using ANCOVA; ^b^p value correspond to tests of the adjusted between-group difference; EORTC_QLQ: The EORTC QLQ Core Questionnaire (EORTC QLQ-C30); SarQol: The Sarcopenia Quality of Life Questionnaire. | | | | | | | | | | | | | |

| Table 6. Baseline-adjusted ANCOVA with HC3 standard errors | | | | | | | | |
| --- | --- | --- | --- | --- | --- | --- | --- | --- |
|  |  | Change from Baseline | | | Adjusted Group Difference^a^ | | |  |
| Outcome | Placebo | | Creatine | |  |  |  |  |
|  | n | mean ± SD | n | mean ± SD | Mean | SE | 95% CI | p value^b^ |
| **Muscular Strength** |  |  |  |  |  |  |  |  |
| Grip Strength (lbs) | 11 | 0.62 ± 2.70 | 13 | 0.17 ± 4.19 | 0.06 | 1.60 | [-3.08, 3.20] | 0.97 |
| Chest Press (lbs) | 11 | 6.80 ± 3.80 | 13 | 8.55 ± 5.64 | 0.44 | 1.75 | [-3.00, 3.87] | 0.81 |
| Leg Extension (lbs) | 11 | 11.75 ± 7.23 | 13 | 12.99 ± 8.61 | 2.07 | 4.02 | [-5.81, 9.94] | 0.61 |
| **Body Composition** |  |  |  |  |  |  |  |  |
| LST (kg) | 11 | 0.58 ± 2.08 | 13 | 1.87 ± 2.91 | 0.97 | 1.30 | [-1.58, 3.52] | 0.46 |
| ALM (kg) | 11 | 0.56 ± 1.02 | 13 | 1.59 ± 1.36 | 0.48 | 0.55 | [-0.60, 1.55] | 0.40 |
| ALM/height^2^ (kg/m2) | 11 | 0.18 ± 0.36 | 13 | 0.46 ± 0.42 | 0.23 | 0.20 | [-0.16, 0.62] | 0.26 |
| Fat Mass (kg) | 11 | -0.67 ± 1.94 | 13 | -1.05 ± 2.45 | 0.02 | 0.96 | [-1.86, 1.91] | 0.98 |
| Visceral Fat (kg) | 11 | -0.02 ± 0.10 | 13 | -0.06 ± 0.16 | −0.04 | 0.05 | [-0.14, 0.07] | 0.50 |
| Body Fat % | 11 | -0.84 ± 2.44 | 13 | -1.29 ± 2.35 | −0.46 | 1.07 | [-2.55, 1.64] | 0.67 |
| BMD (g/cm^3^) | 11 | 0.01 ± 0.01 | 13 | -0.01 ± 0.03 | −0.02 | 0.01 | [-0.04, 0.00] | 0.11 |
| **Physical Function** | 11 |  | 13 |  |  |  |  |  |
| SPPB (AU) | 11 | 0.27 ± 0.47 | 13 | 0.85 ± 1.52 | 0.43 | 0.30 | [-0.17, 1.03] | 0.17 |
| **Quality of Life** |  |  |  |  |  |  |  |  |
| Global Health Status | 11 | 12.12 ± 13.62 | 13 | 4.49 ± 13.01 | −6.86 | 4.60 | [-15.87, 2.15] | 0.15 |
| **Functional Scales** |  |  |  |  |  |  |  |  |
| Physical Functioning | 11 | 3.64 ± 10.48 | 13 | 2.56 ± 6.96 | −0.16 | 3.05 | [-6.13, 5.82] | 0.96 |
| Role Functioning | 11 | 10.61 ± 18.67 | 13 | 5.13 ± 10.51 | 2.13 | 2.87 | [-3.49, 7.76] | 0.47 |
| Emotional Functioning | 11 | 14.39 ± 18.29 | 13 | 0.00 ± 4.81 | −5.73 | 2.95 | [-11.51, 0.04] | 0.07 |
| Cognitive Functioning | 11 | 4.55 ± 18.40 | 13 | 1.28 ± 14.37 | 3.89 | 5.30 | [-6.49, 14.27] | 0.47 |
| Social Functioning | 11 | 10.61 ± 13.48 | 13 | -5.13 ± 19.70 | −10.70 | 5.95 | [-22.37, 0.97] | 0.09 |
| **Symptom Scales** |  |  |  |  |  |  |  |  |
| Fatigue | 11 | -18.18 ± 18.77 | 13 | -3.42 ± 15.96 | 4.89 | 4.90 | [-4.71, 14.49] | 0.33 |
| Nausea/Vomiting | 11 | -3.03 ± 10.05 | 13 | 1.28 ± 4.62 | 3.41 | 3.38 | [-3.21, 10.03] | 0.32 |
| Pain | 11 | -9.09 ± 30.15 | 13 | 1.28 ± 15.90 | 1.17 | 6.58 | [-11.72, 14.06] | 0.86 |
| Dyspnea | 11 | -3.03 ± 17.98 | 13 | 7.69 ± 14.62 | 6.15 | 5.99 | [-5.58, 17.89] | 0.32 |
| Insomnia | 11 | -12.12 ± 26.97 | 13 | -7.69 ± 30.89 | −5.76 | 8.23 | [-21.88, 10.36] | 0.49 |
| Appetite Loss | 11 | -3.03 ± 10.05 | 13 | 0.00 ± 13.61 | 1.91 | 5.54 | [-8.94, 12.76] | 0.73 |
| Constipation | 11 | 0.00 ± 14.91 | 13 | -10.26 ± 21.01 | −7.09 | 6.15 | [-19.14, 4.95] | 0.26 |
| Diarrhea | 11 | -15.15 ± 22.92 | 13 | 5.13 ± 26.69 | 16.19 | 10.35 | [-4.08, 36.47] | 0.13 |
| Financial Difficulties | 11 | 3.03 ± 10.05 | 13 | -12.82 ± 21.68 | −13.52 | 12.27 | [-37.58, 10.53] | 0.28 |
| SarQoL | 11 | 12.57 ± 8.88 | 13 | 6.89 ± 6.49 | −5.06 | 3.46 | [-11.84, 1.72] | 0.16 |
| ^a^Adjusted Group Difference represents the estimated difference between the creatine and placebo groups (CrM - placebo) after adjustment for baseline values using ANCOVA; ^b^p value correspond to tests of the adjusted between-group difference; ALM: Appendicular lean soft tissue; LST: Lean soft tissue; BMD: Bone Mineral Density; AU: Arbitrary Units; SPPB: Short Physical Performance Battery; EORTC_QLQ: The EORTC QLQ Core Questionnaire (EORTC QLQ-C30); SarQol: The Sarcopenia Quality of Life Questionnaire. | | | | | | | | |
