# Supplementary material for: Examining the feasibility and preliminary effects of resistance exercise training and creatine supplementation in individuals treated for colorectal cancer
Source: PLoS One. 2026 Jul 15;21(7):e0353630. doi: 10.1371/journal.pone.0353630 (PMC13372120; doi:10.1371/journal.pone.0353630)
Supplement: S3 File — (DOCX) [file pone.0353630.s003.docx]

**Study Title**: The Feasibility and Acceptability of Resistance Training and Creatine Supplementation to Promote Physical Function in Sarcopenic Colorectal Cancer Survivors

**Principal Investigator Name: Ciaran Fairman, PhD**

**A. SPECIFIC AIMS**

We seek to conduct a pilot RCT, examining the effects of a 10-week multimodal resistance exercise and creatine intervention (EXSUPP) (n=20) relative to resistance exercise and creatine placebo (EXPLA) (n=20) in sarcopenic individuals who have previously been treated for colorectal cancer. **Our overarching objectives are** to **1)** determine the feasibility and acceptability of the EXSUPP intervention with **creatine monohydrate supplementation** (0.10 g/kg/day) and **2)** understand the intervention characteristics that can be used to inform the design of a future large RCT. **Our RCT is *significant* because** it will establish the feasibility and acceptability of combining creatine monohydrate with resistance exercise training in the management of sarcopenia in individuals with colorectal cancer, providing preliminary support for the integration of effective lifestyle interventions in the management of colorectal cancer care. This pilot RCT will provide important information for the design of a future large-scale RCT R01 submission. To test our hypothesis and achieve the application objectives, we propose the following ***Specific Aims*:**

**Primary Aims: 1(a) To explore the feasibility and acceptability of a 10-week RCT comparing EXSUPP to EXPLA in individuals who are sarcopenic after colorectal cancer treatment.** This aim will be accomplished by assessing accrual, retention, adherence, and adverse events, **and 1(b) to quantify (mean change scores and precision estimates) and compare between-arm differences in muscular strength,** the primary variable used to diagnose sarcopenia.^2^ We hypothesize that we will be able to recruit (within a 12-month window) 40 colorectal cancer survivors, retain ≥75% of the sample, and achieve ≥75% adherence to the resistance exercise intervention and ≥75% adherence to creatine/creatine placebo supplementation, with no adverse events experienced throughout the intervention. For aim 1(b), we hypothesize that the EXSUPP arm will experience a more favorable muscle strength response than the EXPLA arm following the intervention. The trial will be considered a success if feasibility outcomes are achieved, and the hypothesized directionality of the EXSUPP intervention effects on muscular strength is observed.

**Aim 2. To obtain mean change scores and precision estimates and compare between-arm differences in secondary outcomes:** (a) body composition (whole-body and appendicular lean mass, whole-body fat mass and bone mineral content and density) (b) physical function, and (c) health-related quality of life measures.

**Exploratory Aim:** **Explore muscle molecular-level adaptations, i.e., mitochondrial health and protein turnover, in response to the interventions.** Cancer treatments have negative consequences on muscle cell signaling, mitochondrial health, and inflammation, and we will examine how these respond to exercise.

**B. BACKGROUND AND SIGNIFICANCE**

**
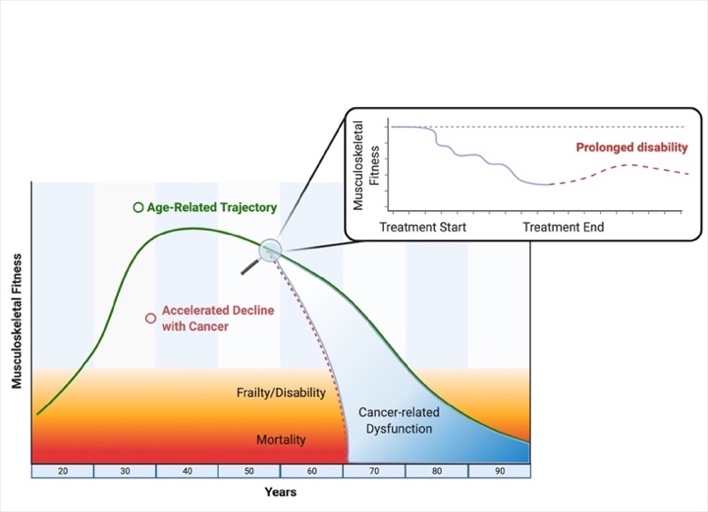
1.Significance: 1a. Sarcopenia is associated with reduced quality of life and reduced survival in colorectal cancer.** Colorectal cancer is the third most common cancer in the world.^3-5^ Sarcopenia, defined as the age-loss of skeletal muscle strength, lean mass and physical function,, affects up to 60% of individuals with colon cancer.^3-6^ Sarcopenia is associated with post-treatment complications and cancer-related and all-cause mortality.^7^ Individuals with colorectal cancer who are sarcopenic experience a 23% reduction in recurrence-free survival and a 9% reduction in cancer-specific survival.^8^ Left unaddressed, sarcopenia results in profound reductions in physical function and quality of life, and heightens mortality risk (Figure 1).

**Figure 1.** Accelerated musculoskeletal decline with treatment

**1b. Cancer treatments accelerate musculoskeletal decline and worsen outcomes.** Cancer treatments induce an accelerated aging phenotype that results in progressive loss in muscle strength, lean mass and physical function.^9-11^ Alterations in molecular signaling in **
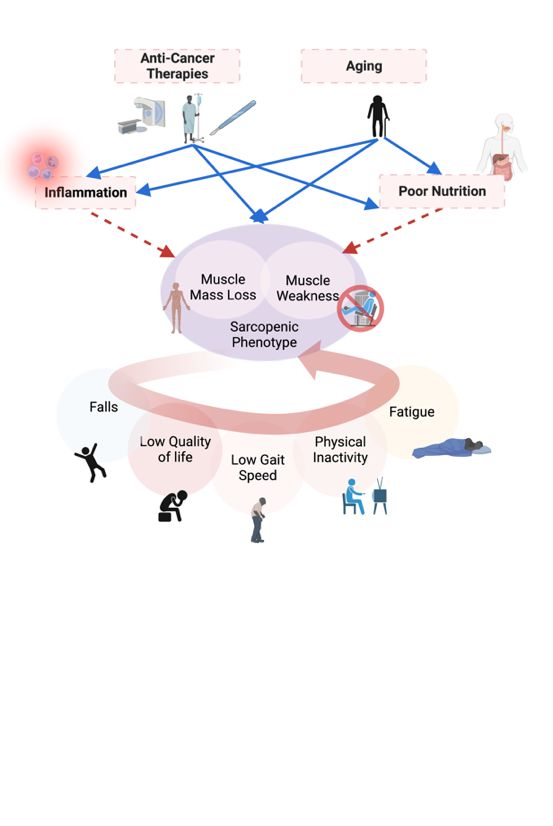
**muscle as a result of cancer treatments exacerbate muscle dysfunction.^12,13^ In colorectal cancer, loss of lean mass during treatment is associated with a two- to four-fold increase in mortality risk.^14-16^ This is compounded by recent findings that at 6 months post-diagnosis, 54% of individuals diagnosed with colorectal cancer did not recover their pre-operative levels of physical activity..^17^ Consequently, colorectal cancer treatments exacerbate lean mass loss, accelerating the trajectory of a sarcopenic condition, poor functional status, and reduced quality of life(Figure 2).^14-16^

**Figure 2.** Contributions to and consequences of sarcopenia


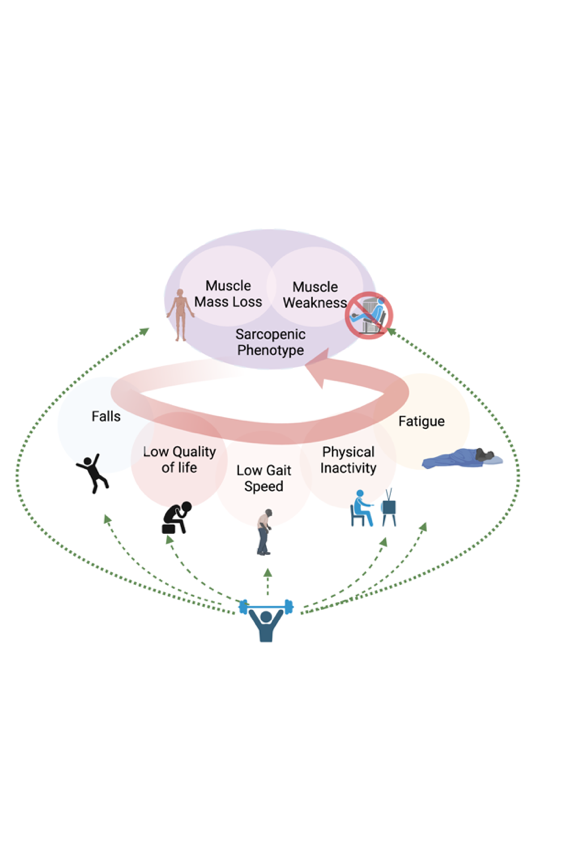
**1c. Resistance exercise is a potent countermeasure to Sarcopenia.** Resistance exercise remains one of the most potent therapeutic strategies to target and potentially reverse the core components and consequences of sarcopenia (reduced muscle strength, lean mass and physical function) (Figure 3).^18-22^ Specifically, resistance exercise has been regularly demonstrated to have beneficial effects on muscle strength, lean mass and measures of physical function in a variety of cancer types.^21,23-28^ We have previously demonstrated that lifestyle interventions incorporating resistance exercise improve muscle strength, physical function (i.e. mobility) and lean mass in men with prostate cancer receiving androgen deprivation therapy.^29^ Importantly, in line with consensus statements from various international working groups, the goal of interventions in this space is not necessarily to *reverse sarcopenia*.^30,31^ Rather, successful interventions are those that improve clinically relevant outcomes such as muscle strength, physical functional status and health-related quality of life.^32,33^ Consequently, there is compelling evidence to suggest that resistance exercise training could target most, if not all components of sarcopenia, ultimately progressing an individual to a more robust status.

**Figure 3.** Resistance exercise as a countermeasure to sarcopenia


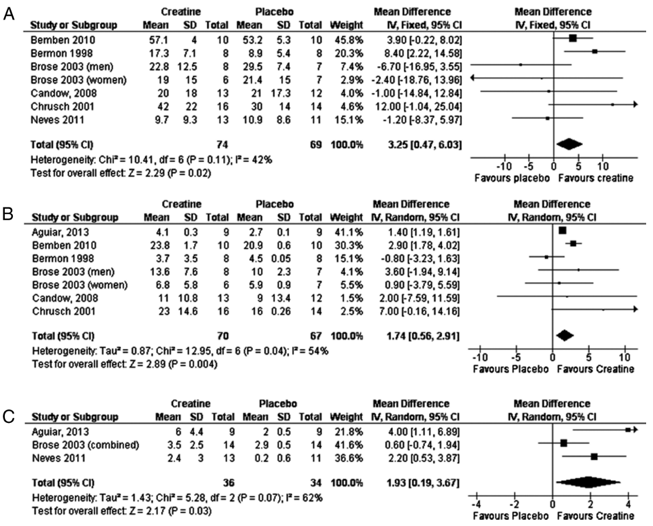


**1d. Creatine Supplementation augments adaptations to resistance training.** Resistance exercise alone is likely to be insufficient to completely overcome the multifactorial mechanisms influencing low muscle strength, lean mass and physical function in individuals with sarcopenia.^34^ Creatine is a naturally occurring compound synthesized in the body that plays a critical role in energy provision during resistance exercise. ^35-41^ Importantly, the rate of endogenous synthesis in the kidneys and liver (i.e. 1-3 g/day) and exogenous consumption of creatine through habitual dietary intake (primarily from red-meat and seafood), is roughly equivalent to the rate of excretion in the form of urinary creatinine (metabolic by-product of creatine).^36,42^ As such, the most potent and efficient way to increase intramuscular creatine stores is through supplementation. Creatine monohydrate is the most widely studied nutritional supplement to date, with well over 1,000 studies establishing its safety and effectiveness across the lifespan.^1,38,39,43-54^ Most relevant, creatine supplementation has been demonstrated to consistently yield superior improvements in lean mass when combined with resistance exercise training, relative to resistance training alone in older adults.^1,55^ Our group and others have also demonstrated superior improvements in muscle strength, lean mass and physical function with creatine supplementation and resistance training, relative to resistance training alone, particularly in older adults(Figure 3).^1,44,55^ Mechanistically, creatine supplementation may improve muscle protein kinetics, growth factors and satellite cells, calcium and glycogen flux, inflammation and oxidative stress, which could in turn improve sarcopenic status.^55-57^ The consistent and reliable improvements in muscle strength, lean mass and physical function indicate overwhelming potential for creatine supplementation to augment adaptations to resistance exercise and improve clinically relevant outcomes in individuals with colorectal cancer.

**Figure 3.** Creatine vs placebo effects on A) upper body strength, B) lower body strength and C) sit-to-stand^1^.

**1e. The need to understand molecular and cellular underpinnings that contribute to accelerated aging in cancer and how they impact the response to training.** An “accelerated aging” phenotype has been conceptualized to describe pathogenesis of anti-cancer therapies on key hallmarks of aging including DNA damage, inflammation and oxidative stress.^10,11,58^ Numerous mechanisms from preclinical models have been proposed as primary contributors to muscle dysregulation including skeletal muscle signaling, mitochondrial dysfunction, and heightened inflammation during treatment.^59-62^ For example, the ubiquitin proteasome system and autophagy-lysosome pathways have both been proposed as contributors to skeletal muscle atrophy with doxorubicin.^63-65^ Additionally, oxidative stress as a result of mitochondrial dysfunction has also been proposed as a contributor to doxorubicin and 5 fluorouracil induced atrophy.^66-70^ Interestingly, systemic inflammation (increases in Interleukin-6, TNF- α, Interleukin-1, etc.) has been associated with loss of muscle mass and function in other cancers and with other treatments.^12,71-75^ However, despite preclinical evidence supporting some of the proposed mechanisms, to date few clinical studies have investigated cellular alterations that contribute to changes in muscle biology because of treatment, and how this impacts exercise tolerance/adaptations. Investigating mechanisms of cancer-related musculoskeletal decline could help identify unforeseen barriers and targets for therapeutic interventions.

**C. PRELIMINARY STUDIES**

This is a feasibility trial based on conversations with colleagues in the field of exercise oncology. There is a major gap in the literature of well-designed RCT’s combining resistance exercise and nutrition as an intervention for sarcopenic individuals after treatment for colorectal cancer. Importantly, one of the biggest gaps in the field of exercise oncology is the lack of interventions targeting treatment-related impairments, that specifically enroll individuals with an impairment.^25,76,77^ Our trial will be one of the first to address this gap by objectively characterizing individuals as sarcopenic, prior to enrollment. This will advance the field by furthering our understanding of the feasibility and challenges of implementing lifestyle interventions to individuals with sarcopenia after colorectal cancer treatment.

Despite strong evidence from other populations supporting the potential of combining resistance exercise and creatine supplementation to improve physiologic and QOL outcomes that cancer treatments adversely affect, evidence specific to cancer remains scarce. ^44,55,78-81^ Critical first questions in this line of inquiry are: 1) if individuals who are sarcopenic after treatment for colon cancer can be recruited to lifestyle interventions; and 2) is our proposed intervention combining resistance exercise and creatine supplementation feasible for this population. We contend the proposed study is innovative in that it would be the first to implement and test this evidence-informed intervention in individuals who are sarcopenic after colon cancer. The proposed RCT builds on several aspects of our team’s prior research investigating the effectiveness of resistance exercise, diet and creatine supplementation in older adults and individuals with cancer.^29,82-84^ Additionally, we have an ongoing RCT with creatine supplementation in prostate cancer that has been delayed due to COVID-19.^82^ However, we have recruited 30 individuals so far, with no adverse events, and we have demonstrated the safety, feasibility and tolerability of supplementation in other cancer populations. Members of our team have investigated the molecular regulators of skeletal muscle mass and how they impact the response to exercise and nutrition interventions.^85-87^ Moreover, we have adhered to NIH guidelines on pilot studies and framework for clinical research, with a focus on obtaining data to optimize the intervention procedures and inform the design of a future randomized controlled trial.^88,89^

**D. RESEARCH DESIGN AND METHODS AND DATA ANALYSIS**

**Location:** Pre- and post-intervention data collection and exercise sessions take place at the University’s Department of Exercise Science.

**Intervention: *Resistance Exercise Training****.* Participants assigned to each arm will attend a total of 30 supervised exercise sessions for 10 weeks (thrice weekly, with a minimum 48 hours rest between sessions). This intervention is commonly employed in exercise oncology and has yielded high rates (~80%) of attendance and has not resulted in any adverse events.^18,24,27,28,90-94^ All exercise sessions will occur in the Sports Science Lab at the University of South Carolina, supervised by members of the research team specifically trained in exercise oncology. For the intervention, participants will be asked to perform 8 total movements targeting total body musculature. The exercise selected to target each of these movements will be multi-joint, compound movements, tailored to each participant in accordance with the clinical judgement of trained study staff in the trial. Sessions will be progressed by adding sets to the exercises and increasing load as appropriate and tolerated (i.e. in exercises where no load can be added, a focus will be on adding sets). Specifically, trained exercise physiologists in the trial will select the appropriate exercises, providing coaching cues for safe movement and tailored exercise sessions. **Intervention Fidelity:** Exercise intervention fidelity will be reported using metrics previously outlined by Fairman et al (2019).^24^ Specifically, volume load will be calculated as a function of sets x reps x weight for each exercise and summed to give total volume for each session. We will report the proportion of volume achieved relevant to what was prescribed to give a “relative-dose intensity” (RDI) for each person. RDI will then be averaged and used to determine fidelity to the RT intervention.

**Supplementation.** Participants in the creatine group will receive creatine monohydrate supplementation (Optimum Nutrition, USA) at a dose of 0.10g/kg/day. This dose has been used in RCT’s involving older adults and individuals with cancer.^82,83^ Participants in the placebo group will receive a creatine placebo supplement (corn-starch maltodextrin; *Bulk Supplements, Nevada, USA*) in the same dose and schedule as EXSUPP. Participants will be instructed to consume 0.05g/kg/day of their supplement with food in the morning and 0.05g/kg/day with food in the afternoon or evening. Both groups will have similar flavor supplements to mask taste and solubility. **Supplement Adherence:** Supplements will be provided using smart-pill bottles (Pillsy, Inc; Seattle, WA), that will be used to automatically track number of times bottles are opened and return estimates of compliance to supplementation. The smart-pill bottles are accompanied by an app, which tracks when the bottle is opened, tracks remaining doses and has reminders/alarms for missed doses. For adherence, it is assumed that opening the bottle cap signifies consumption of a daily dose. As such, adherence to supplementation will be quantified as number of daily doses consumed across the intervention, relative to what could have been consumed. The reliability of smart pill bottles to track medication adherence has been published recently and has been demonstrated to improve adherence in cancer.^95-97^

**Study Outcomes:** Study outcomes will be assessed within 1 week of initiation and completion of the intervention.

**Primary Outcome:** The primary outcome of feasibility will be evaluated by **Response rates** (cases responding/cases ascertained (minus letters returned/not delivered & disconnected phone lines), and **reasons for ineligibility/disinterest** **and proportional accrual** (cases enrolled/cases ascertained) will all be tracked to inform feasibility of the trial. **Retention** will be calculated as the proportion of individuals who return for follow-up testing. **Adherence** will be calculated as proportion of total exercise achieved (outlined in “Intervention Fidelity”), and proportion of daily supplementation taken. **Adverse events** will be recorded, including a description of the event, its relation to the intervention, level of seriousness and intensity. To better understand program feasibility and acceptability, individuals will be asked to participate in qualitative in-depth exit interviews at the end of the study. A semi-structured interview guide will include questions that prompt participants to elaborate on their experiences and perceptions of the program and suggestions for how the program can be improved. Interviews will be audio-recorded, transcribed verbatim, and coded using inductive thematic analysis with Nvivo software(QSR International, Burlington, MA; USA).

**Secondary Outcomes:**

**Muscular Strength:** Leg press and chest press strength will be assessed using a 1-repetition maximum testing protocol. Following a standardized warm-up, participants will be asked to complete two exercise-specific warm-up attempts at 5-6 repetitions with ~50% estimated 1RM. Participants will complete another warm-up attempt with 3 repetitions at ~80% 1RM. Participants will then be asked to complete a maximal attempt of 1 repetition at the maximal load they can lift. A 1RM value will be recorded when participants can no longer lift the weight with safe technique. Handgrip strength will be assessed via handgrip dynamometry (Jamar Plus+; Sammons Preston, Rolyon, Bolingbrook, IL) due to the association of low grip strength with functional limitations, reduced quality of life, and mortality in aging.^2,77^

**Anthropometric Battery.** *Body weight* will also be measured to the nearest 0.1 kilogram using a calibrated and certified balance beam scale. *Height* without shoes will be measured to the nearest 0.1 centimeter using a stadiometer. *BMI* will be calculated using the body mass and height values.

***Physical Function: Short Physical Performance Battery Protocol (sPPB)*** The sPPB will be used to assess lower body physical function, comprising of assessments of balance (tandem and semi tandem), gait speed (4-meter walk test in m/s), and chair stand (5 times repeated chair stand). The sPPB is scored by summing the scores of each test, with lower scores indicating worse functioning.^98^ Self-reported physical activity will be obtained via the International Physical Activity Questionnaire, at baseline, follow-up and bi-weekly throughout the intervention to understand physical activity achieved outside the intervention.^99^

**Health-Related Quality of Life (HRQOL).** Cancer-specific HRQOL indices will be measured using the European Organization for Research and Treatment of Cancer QoL Questionnaire Core 30 (EORTC-QLQ-C30). Sarcopenia-related HRQOL will be assessed using a sarcopenia-specific questionnaire (SarQoL).^100^

**Blood Biomarkers:** Venous blood samples will be obtained into 10 mL vacutainer tubes using a 21-gauge needle inserted into the median cubital vein as described in our laboratory’s standard operating procedure [See Blood Draw SOP]. Blood samples will be stored at room temperature for 10 min and then centrifuged. Serum will be removed and frozen at -80C for later analysis. Total plasma concentrations of inflammatory mediators will be determined using quantitative sandwich ELISA kits provided by R&D Systems, Inc. (Minneapolis, MN). TNF-α (Cat. HSTA00D; sensitivity = 0.19ρg/mL), IL-6 (Cat. HS600B; sensitivity = 0.11ρg/mL) and CRP (Cat. DCRP00; sensitivity 0.022ng/mL) will be measured in all plasma samples. These markers have been linked to disease progression and muscle loss in cancer [3, 46-49].

*Banking for future analysis—*blood will be banked for later assessment of the correlation between genetics/genomics (DNA) or transcription (RNA) and muscle function as well as the levels of biomarkers related to inflammation and cancer progression.

**Skeletal Muscle Mass Regulation, Inflammation, and Mitochondrial Health:** Percutaneous muscle biopsies will be obtained from the middle portion of the vastus lateralis muscle of the dominant leg (midpoint between the patella and the greater trochanter of the femur) at a depth between 1 and 2 cm after subcutaneous administration of the local anesthetic, lidocaine (1 mL of 1% lidocaine) as previously performed by our group [61-66] and as described in our laboratory’s standard operating procedure [See Micro-Biopsy SOP]. After removal, adipose tissue will be trimmed from the muscle specimens and will be immediately snap frozen in liquid nitrogen (LN_2_) and stored at −80̊C for later analysis or place in 2.5% glutaraldehyde for electron microscopy. Protein will be extracted from frozen skeletal muscle biopsies and total protein content will be measured by the standard Bradford protein assay. Intramuscular markers of mitochondrial dynamics (PGC-1a, TFAM, Drp-1, Fis-1, MFN-1, MFN-2, Opa-1, BNIP3, parkin, LC3, P62, cytochrome C), content (complex I-V content, citrate synthase activity, VDAC) will be assessed. Alterations in these markers of mitochondrial quality control have been associated with muscle atrophy and sarcopenia [50-53]. Additionally, we will use electron microscopy to assess skeletal muscle mitochondrial density, morphology, and ultrastructure to further characterize skeletal muscle mitochondrial quality. Intramuscular markers of skeletal muscle anabolism (Akt, mTORC1, p70s6k) and catabolism (ubiquitin, myostatin, MuRF1/Atrogin1) will be assessed [26, 47, 54]. Skeletal muscle mass is tightly regulated by the balance between these anabolic and catabolic pathways [55-60] While not an exhaustive list, these markers are well established regulators of muscle mass, inflammation, and mitochondrial health.

*Banking for future analysis*—muscle will be banked for later assessment of protein signaling, gene expression, and other morphometric outcomes. Total protein, DNA, and RNA will be isolated according to standard procedures and then stored at -80°C.

**Statistical Analyses:** For this feasibility study, we hypothesize that we will be able to recruit (within a 12-month window) 40 cancer survivors, retain ≥75% of the sample, and have ≥75% adherence to the intervention.^101^ In line with NIH guidelines on pilot studies, data obtained from these tests will be critical to optimize the intervention procedures and inform the design of a future RCT.^88^ **Further, the NIH reinforces that the “goal of pilot studies is to assess feasibility/acceptability of an approach to be used in a larger scale study”**^89^ Rather, our **sample size** is based on “practical considerations regarding participant flow, budgetary constraints, and the number of participants needed to reasonably evaluate feasibility goals.”^89,102^ To evaluate estimates of effectiveness for a larger RCT, we will test for differences in change in mean concentrations of muscle strength, body composition, physical function, quality of life, biomarkers (IL-6, TNFα, CRP) and mitochondrial health/muscle cell signaling among the groups using analyses of variance. We will calculate Cohen’s d by dividing the difference in group means (control versus supplement groups) by the pooled standard deviation. We will test for relations between 1) baseline levels of fatigue and inflammatory biomarkers and 2) changes in biomarkers and changes in fatigue using Pearson or Spearman’s correlations

**Timeline:** Dr. Fairman has almost a decade of experience in executing exercise trials with cancer patients. We have an established infrastructure for recruiting individuals with cancer, testing our outcomes, and delivering interventions. Developing our database, training personnel, trial registration and submission of protocol paper for publication, will occur in the first 3 months of the grant. Participant recruitment will begin in month 4 of year one. Specifically, we will aim to conduct the intervention in 5 waves of 8-10 individuals in each wave. We anticipate identifying 4-5 eligible and interested individuals per month, allowing us to achieve our target or 8-10 individuals per wave across the study timeline.

Recruitment for subsequent waves will be ongoing during intervention delivery of preceding waves (i.e., recruitment for wave 2 will be ongoing while wave 1 is completing the intervention). Data Collection will begin in Months 4/5 of year one and continue until month 10 of year two. The remaining of year two will be dedicated to analyses, manuscript preparation and presentation/publications.

|  | **Y1** | | | | **Y2** | | | |
| --- | --- | --- | --- | --- | --- | --- | --- | --- |
|  | **Q1** | **Q2** | **Q3** | **Q4** | **Q1** | **Q2** | **Q3** | **Q4** |
| **Development** |  |  |  |  |  |  |  |  |
| Preregister trial on clinicaltrials.gov | x |  |  |  |  |  |  |  |
| Submit Protocol Paper for Publication | x |  |  |  |  |  |  |  |
| Finalize Protocols | x |  |  |  |  |  |  |  |
| IRB Approval | x |  |  |  |  |  |  |  |
| Hire/Train research assistants | x |  |  |  |  |  |  |  |
| **Recruitment/Intervention** |  |  |  |  |  |  |  |  |
| Recruitment |  | x | x | x | x | x |  |  |
| Intervention Delivery |  | W1 | W2 | W3 | W4 | W5 |  |  |
| Assessments |  | x | x | x | x | x |  |  |
| Post Testing |  |  | x | x | x | x | x |  |
| **Data Analysis/Publication** |  |  |  |  |  |  |  |  |
| Data cleaning and analysis |  |  |  |  |  |  | x | x |
| Report Clinical Trial Results on clinicaltrials.gov |  |  |  |  |  |  |  | x |
| Present/Publish Results |  |  |  |  |  |  |  |  |

Table 1. Project Timeline

Participants recruited and enrolled in intervention in 5 waves (W1, W2 etc.) of 8-10 participants. Quarterly timepoints are estimates.

**E. PROTECTION OF HUMAN SUBJECTS**

1.  TARGET POPULATION:

Individuals ≥12 months post treatment for colorectal cancer (n=40), who are over the age of 18 and meet the criteria for sarcopenia will be eligible to participate. About 90% of those who get colorectal cancer are older than 40, and in the rare instances of a colorectal cancer diagnosis under the age of 18, cancer risk and survival is not as strongly linked to lifestyle factors such as physical activity. In addition, treatment strategies and subsequent side effects also differ between child and adult survivors of cancer. Furthermore, the proposed resistance exercise protocols are designed for adults, and may not be appropriate for children. There will be no upper limit placed on age. About a third of the individuals who are diagnosed with colorectal cancer are 65 years or older. Therefore, we anticipate the majority of our subject population will be mid-life and older adults. The investigative team has expertise working with adults and older adults. Dr’s Fairman and Candow have experience conducting interventions with older adults living with and without cancer, ranging from 18-85. In addition, as medical oncologist Dr.’s Kim treats patients ranging from 18 years old to end of life. Depending on the age distribution of participants who enroll, age may be considered as a covariate in analyses as needed.

Twelve months post treatment or greater was chosen to allow full recovery from the acute side effects of treatment that might influence exercise tolerance. Specifically, the SARC-F questionnaire will be used to screen individuals. The SARC-F is a self-administered questionnaire used to determine the level of difficulty experienced in 5 components comprising the strength, assistance in walking, rising from a chair, climbing stairs, and history of falls; a score of 0–2 points is assigned for each item. The total score ranges from 0 to 10, with scores of ≥4 points indicating the risk of sarcopenia. Participants will be excluded if they 1) are receiving active treatment for their cancer; 2) have a any contraindication to exercise participation; 3) have been participating in structured resistance exercise 2 or more times per week for the past 6 months; 4) are currently taking supplements containing creatine for 4 weeks prior to the start of the RCT, or 5) are receiving medications that might alter body composition (metformin, corticosteroids etc.).

A convenience sample (n=40) will be recruited to determine the feasibility of the intervention. The rationale for this is in line with recommendations from Julious et al (2005) that justify an n=12 as a minimum based on feasibility, gains in precision surrounding the mean and variance, and ability to estimate parameters for future studies.^103^ Consequently, given that our study has two arms, and in line with other exercise oncology trials in anticipation of an ~75% retention rate, we aim to recruit 20 individuals per arm to participate in full study activities.

2. RECRUITMENT PLANS:

Recruitment will occur in collaboration with two healthcare partners (PRISMA Health and South Carolina Oncology Associates, [SCOA]). SCOA is a medical and radiation oncology practice affiliated with an ACOS-approved Integrated Network Cancer Program in central South Carolina. PRISMA Health-Midlands provides multidisciplinary cancer care in Columbia that is a part of the larger PRISMA Health system. Participants will be recruited by 1) use of cancer registry data bases at local health care system’s cancer programs to prepare personalized invitations, and 2) outreach with various cancer support groups and organizations. We will also request a partial HIPAA waiver from the cancer clinics to recruit from patient lists of contact information. Specifically, the covered entity (Prisma), will provide us with a secure list of individuals previously treated for colorectal cancer, and their PHI. This is one of our strongest methods of recruitment and are currently using it in several ongoing studies. From these lists, we will send an initial introductory letter to eligible patients from their oncology provider with information about the study. There will be a number to call if they do not wish to be contacted by study personnel. After a wait time ~ 2 weeks, study staff will begin contacting the list of potential participants. At initial contact, study recruiters will provide a detailed account of the study and its procedures, answer any questions, ascertain interest, and, if interested, schedule the baseline visit.

Based on our recruitment goals and the proportion of individuals we anticipate being interested (~50%) and eligible (~50% of interested individuals), we will mail flyers and invitations in batches of 200. We will schedule mailings to occur approximately every 4-6 weeks until recruitment goals are met. If recruitment numbers are insufficient, we will increase both the size and frequency of mailings. In the event that recruitment numbers are insufficient, we will work more closely with oncologic and medical providers to promote participation in the study. Moreover, we will closely monitor the response to mailings and adjust our efforts accordingly. Specifically, mail flyers and invitations will be mailed in batches of 200. We will schedule mailings to occur approximately every 4-6 weeks until recruitment goals are met. If recruitment numbers are insufficient, we will increase both the size and frequency of mailings. Further, we will pursue more direct approaches of visiting colorectal clinics, cancer support groups, churches etc. Based on the prior work of our team in the area of cancer survivorship, we are confident these strategies will result in the recruitment of our desired number.

Individuals will be contacted by study staff and will be provided detailed information on the study purpose/aims, protocol, potential benefits and risks and answer further eligibility questions. If a participant meets initial eligibility criteria, they will be directed to an online Physical Activity Readiness Questionnaire (PARQ+; www.eparmedx.com) to ensure safety for exercise and determine whether physician clearance is required. Individuals who are eligible and remain interested will be asked to provide informed consent to participate prior to any study activities. This method of recruitment has proved successful in our prior exercise oncology trials.

3. EXISTING DATA/SAMPLES:

*N/A*

4. CONSENT/ASSENT

The following processes for consent will be conducted as follows:

- Mailings will be sent to potential participants that include a description of the study, it’s purpose, anticipated benefits and risks. The mailing will include a link to provide information for study staff to contact them.
- Once connected, a member of the study team will undergo an initial phone screening with individuals to provide a brief overview of the study, reinforce the anticipated risks and benefits, and that participation is completely voluntary. The phone screening will also include questions regarding the eligibility criteria to determine if the participant is suitable and eligible for the trial.
- Individuals who are interested and eligible will then be provided with further details regarding study participation and scheduled for a baseline assessment. Individuals will be emailed a copy of the informed consent form to review prior to arrival at the lab for baseline testing.
- Once participants arrive for baseline testing, a member of the study team will review the consent form in detail and answer any further questions participants might have. Every effort will be made by study staff to ensure the informed consent is understood in its entirety prior to signing. At this point, individuals will also be reminded that participation is completely voluntary, and that withdrawal is allowed at any point in the study without penalty.
- Individuals will be provided asked to provide “yes” or “no” options to consent to participate in the study.
- The signed document will be collected and catalogued by trained research staff.

5. POTENTIAL RISKS

**Potential Risks.** There are no foreseeable major risks or side-effects associated with participation. However, as is the case with anyone who exercises, participation in the exercise intervention may result in mild discomfort and muscle soreness. There is also the possibility of muscle pulls or strains associated the exercise program, common to any type of physical activity. In order to minimize these risks participants will perform an adequate warm-up and cool-down before and after any exercise bout, be comprehensively instructed on the correct lifting technique, thoroughly familiarized with the movements involved in this investigation and supervised at all times by qualified professionals. If participants experience discomfort, soreness, or any issues with their prescribed exercise, the exercise prescription will be modified accordingly. Risk of falling may exist in the performance of some tasks, however, participants will be closely supervised to minimize the risk of a fall occurring. Furthermore, during exercise it is possible to experience symptoms such as shortness of breath, abnormal blood pressure, fainting, light-headedness, muscle cramps or strain, nausea, and in very rare cases heart rhythm disturbances or heart attack. These potential risks are common to any form of physical activity. During exercise training in other clinical populations, such as cardiac rehabilitation, rates of serious adverse events are very low at 1 in 49,565 patient training hours. Participants may also experience some anxiety in answering the items in the questionnaires.

Creatine is one of the most widely researched supplements, with over 1,000 studies confirming its safety in a variety of populations. As such, the risks of any possible side effects are minimal. However, participants receiving creatine might experience some mild gastrointestinal disturbance. The most commonly reported gastrointestinal disturbance is a mild stomach ache, or excess gas/bloating/loose stool. These side effects have been previously reported but occur in less than 5% of individuals supplementing with creatine. If individuals experience gastrointestinal distress, we will monitor the situation and they will be advised to stop supplementation if necessary. Due to the rarity and mild nature of symptoms, we don’t anticipate any lasting adverse reactions.

Complications resulting from the muscle biopsy are rare, especially in this case where the micro-biopsy procedure is similar to receiving a routine intramuscular injection. To help avoid risk of infection, the participant will be instructed to leave the band-aid on for 24 hours. The participant will be further advised to refrain from vigorous physical activity with the affected leg for 24 hours after the biopsy. There is a potential risk of an allergic reaction to the Lidocaine. All participants will be asked if they have known allergies to local anesthetics (e.g. Lidocaine, Xylocaine, etc.) that they may have been previously given during dental or hospital visits. Participants with known allergies to anesthesia medications will not be allowed to participate in the study. This procedure may cause a small amount of pain when the needle is inserted to subcutaneously inject the Lidocaine and participants may also experience some dizziness, nausea, and/or faint if unaccustomed to needles. However, due to the localized effects of the anesthetic, the participant should feel no pain during this process. In addition, the micro-biopsy procedure may cause some bleeding and bruising. If needed, the subject may take non-prescription analgesic medication such as acetominophen after the completion of the study to relieve pain if needed. However, medications such as aspirin, Advil, Nuprin, Bufferin, or Ibuprophen will be discouraged as these medications may lead to ecchymosis at the micro-biopsy site. Although the muscle selected for micro-biopsy (vastus lateralis) has no major blood vessels or nerves in the areas where the biopsy needle will be inserted, there is the rare occurrence of compressing or cutting small nerve branches, which can sometimes cause temporary tingling and numbness in the skin. These responses, when they have occurred, have dissipated in a few days. Soreness of the area may occur for about 24 hours post-biopsy. Thomas D. Cardaci MS, PhD(c) (under the supervision of E. Angela Murphy, PhD) is trained in muscle biopsy techniques and will be the only study personnel performing the muscle micro-biopsies. He is also trained in phlebotomy techniques and will also be one of the study personnel performing blood draws. Additionally, Ciaran Fairman, PhD is trained in performing phlebotomy techniques and will also be performing blood draws. A telephone is in both laboratories in case of any emergencies, and there will be no less than two researchers working with each participant during the study visit. In the event of any unlikely emergency one researcher will check for vital signs and begin any necessary interventions while the other researcher contacts University of South Carolina campus police or 911.

**Protection Against Risk.** The risk of adverse events with exercise will be minimized by the supervision of exercise sessions by trained exercise physiologists on the study team. Further, the exercise sites have detailed emergency action procedures in place in the unlikely event that medical assistance is needed. These procedures include: 1) contacting emergency services (i.e., calling 911), 2) administering first aid, cardio-pulmonary resuscitation (CPR), and automated external defibrillation (AED) as appropriate, and 3) managing the situation until emergency services arrive. All staff are required to have current CPR and AED certification. Emergency action procedures are reviewed and practiced on a quarterly basis.

All survey/questionnaire responses will be kept strictly confidential. Participants will be informed that if they do experience discomfort or anxiety in filling out the questionnaire, they can choose to stop the assessment. If our study identifies that a participant is experiencing significant distress, he/she will be contacted, and their permission will be sought to inform their GP or cancer specialist so that referral to appropriate services can be made. Participants are asked to notify study staff immediately if they experience discomfort in answering the questionnaire questions. Study materials and data will be coded with numeric identifiers and will be accessed only by the PI. Staff will be trained in human subjects and confidentiality issues and procedures. Study materials and data will be kept in secure computer files and file cabinets at the Arnold School of Public Health, University of South Carolina.

**Adverse Event** The PI (Fairman) will be responsible for monitoring and reporting all adverse events and unanticipated problems. Trained exercise professionals supervising each session minimizes the risk of exercise-related adverse events. Specifically, participants will be monitored for any adverse effects, and other disease-related or exercise-related adverse events using adverse event logs across each patient’s on-trial period, throughout the testing and training sessions. However, we will record and report adverse events in accordance with the CONSORT guidelines. Further, adverse events and unanticipated problems will be documented in accordance with the University of South Carolina’s standards and reported to the Institutional Review Board. Specifically, if an unexpected death occurs, a report will be sent to the Office of Research Compliance immediately. Serious, unexpected events will be reported within 48 hours, and all others within 10 working days. Adverse events will be recorded, including a description of the event, its relation to the intervention (not related, unlikely, possibly, probably, definite), its level of seriousness (i.e. non-serious, required hospitalization, resulted in persistent disability, life-threatening, or resulted in death) and its intensity (mild, moderate, severe, life-threatening). Additionally, the participant will be referred to their general practitioner or specialist, as appropriate for a medical assessment of any adverse event.

6. POTENTIAL BENEFITS:

**Potential Benefits of the Proposed Research to the Subjects and Others.** We believe the potential benefits of participating in this research are quite large. Specifically, individuals will receive 3 months of supervised, individualized exercise training that is specifically designed to improve strength, physical function and health-related quality of life. Moreover, participants will gain a better understanding of how to participate in and progress their exercise safely and effectively. Participants randomized into the supplement group may experience additional benefits in strength, physical function and body composition.

**Importance of the Knowledge to be Gained.** The potential knowledge gained from the proposed project is significant. Specifically, we will be one of the first to specifically implement an intervention targeting sarcopenia in individuals treated for colon cancer. We anticipate that the exercise and nutritional supplementation intervention will yield meaningful improvements in muscular strength, physical function, and body composition. If successful, our findings can offer information on a potentially low-cost therapeutic strategy that can offset the burden of cancer. Further, give the high prevalence of sarcopenia in individuals with cancer, our findings have the potential to support research in other patient populations. The information gained from the exploratory analysis of muscle signaling and mitochondrial health is potentially extremely important. This will be one of the first studies in humans to investigate the long-term effects of cancer treatments on the molecular regulation of muscle mass and how this may impact the response to treatment.

7. CONFIDENTIALITY

Data collected will be de-identified and coded. Each study participant will have a unique study identification code created following informed consent which will comprise two random letters and a consecutive number generated after consent. Data will be and kept electronically, in a password locked folder, on a password locked computer, accessible only by the investigators and designated research staff and will be held in the department of Exercise Science. At the completion of every data collection session, any physical documentation will be stored in a secure filing cabinet with restricted access in a private office in the Department of Exercise Science. All of this information will be held in these secure locations on campus (with password-protection or key-lock access); and will not be stored outside the University. All paper-based records will be stored in a locked filing cabinet with restricted access in a private office for a minimum of 3 years.

Participants will be provided with an explicit description of plans to share any data they contribute, and how their privacy and confidentiality will be protected. All de-identified data and code for analyses will be made available as soon as possible with publication of the primary outcome paper. The dataset will include demographic information, and physiological and psychosocial measures outlined in the proposal. We will make the de-identified data and associated documentation available to users only under a data-sharing agreement that provides for: (1) a commitment to using the data only for research purposes; (2) a commitment to securing the data using appropriate computer technology; and (3) a commitment to destroying the data after analyses are completed. Only researchers from universities, colleges, and research organizations, as determined by the Sponsored Awards Management office at the University of South Carolina, through a formal review process, will be provided these data.

8. COMPENSATION:

A $25 gift card will be given to participants for completing the baseline assessment. A $75 gift card will be given to participants for completing the follow-up assessment. Therefore, total rumination for participants who complete baseline and follow-up assessments will be $100.

Participants will also be offered a $50 incentive to receive an optional muscle biopsy at each assessment timepoint. Therefore, the total rumination for participants who complete both timepoints and receive a muscle biopsy at each timepoint is $200.

9. WITHDRAWAL:

Participants will be informed that their participation in this research is completely voluntary and made to understand that they are allowed to withdraw from the study from any time, without justification. All participants will be asked to inform a member of the study team of their decision and reason to withdrawal but will be informed that their withdrawal will not affect their relationship or any future opportunities to participate in research with the university. No additional information will be collected after an individual withdraws, through data collected up until the point of withdrawal will be used in analyses and interpretation of results.

**F. REFERENCES/LITERATURE CITATIONS**

1. Devries MC, Phillips SM. Creatine supplementation during resistance training in older adults-a meta-analysis. *Med Sci Sports Exerc.* 2014;46(6):1194-1203.

2. Cruz-Jentoft AJ, Bahat G, Bauer J, et al. Sarcopenia: revised European consensus on definition and diagnosis. *Age and Ageing.* 2019;48(1):16-31.

3. Vergara-Fernandez O, Trejo-Avila M, Salgado-Nesme N. Sarcopenia in patients with colorectal cancer: A comprehensive review. *World Journal of Clinical Cases.* 2020;8(7):1188-1202.

4. Nakanishi R, Oki E, Sasaki S, et al. Sarcopenia is an independent predictor of complications after colorectal cancer surgery. *Surgery Today.* 2018;48(2):151-157.

5. Jochum SB, Kistner M, Wood EH, et al. Is sarcopenia a better predictor of complications than body mass index? Sarcopenia and surgical outcomes in patients with rectal cancer. *Colorectal Disease.* 2019;21(12):1372-1378.

6. Broughman JR, Williams GR, Deal AM, et al. Prevalence of sarcopenia in older patients with colorectal cancer. *J Geriatr Oncol.* 2015;6(6):442-445.

7. Brown JC, Caan BJ, Meyerhardt JA, et al. The deterioration of muscle mass and radiodensity is prognostic of poor survival in stage I-III colorectal cancer: a population-based cohort study (C-SCANS). *Journal of Cachexia, Sarcopenia and Muscle.* 2018;9(4):664-672.

8. Miyamoto Y, Baba Y, Sakamoto Y, et al. Sarcopenia is a Negative Prognostic Factor After Curative Resection of Colorectal Cancer. *Annals of Surgical Oncology.* 2015;22(8):2663-2668.

9. De Van Der Schueren MAE, Laviano A, Blanchard H, Jourdan M, Arends J, Baracos VE. Systematic review and meta-analysis of the evidence for oral nutritional intervention on nutritional and clinical outcomes during chemo(radio)therapy: current evidence and guidance for design of future trials. *Annals of Oncology.* 2018;29(5):1141-1153.

10. Guida JL, Agurs-Collins T, Ahles TA, et al. Strategies to Prevent or Remediate Cancer and Treatment-Related Aging. *JNCI: Journal of the National Cancer Institute.* 2021;113(2):112-122.

11. Guida JL, Ahles TA, Belsky D, et al. Measuring Aging and Identifying Aging Phenotypes in Cancer Survivors. *JNCI: Journal of the National Cancer Institute.* 2019.

12. Barreto R, Mandili G, Witzmann FA, Novelli F, Zimmers TA, Bonetto A. Cancer and Chemotherapy Contribute to Muscle Loss by Activating Common Signaling Pathways. *Front Physiol.* 2016;7:472.

13. Schiessel DL, Baracos VE. Barriers to cancer nutrition therapy: excess catabolism of muscle and adipose tissues induced by tumour products and chemotherapy. *Proceedings of the Nutrition Society.* 2018;77(4):394-402.

14. Blauwhoff-Buskermolen S, Versteeg KS, De Van Der Schueren MAE, et al. Loss of Muscle Mass During Chemotherapy Is Predictive for Poor Survival of Patients With Metastatic Colorectal Cancer. *Journal of Clinical Oncology.* 2016;34(12):1339-1344.

15. Miyamoto Y, Baba Y, Sakamoto Y, et al. Negative Impact of Skeletal Muscle Loss after Systemic Chemotherapy in Patients with Unresectable Colorectal Cancer. *PLoS One.* 2015;10(6):e0129742.

16. Derksen JWG, Kurk SA, Peeters PHM, et al. The association between changes in muscle mass and quality of life in patients with metastatic colorectal cancer. *Journal of Cachexia, Sarcopenia and Muscle.* 2020;11(4):919-928.

17. Van Zutphen M, Winkels RM, Van Duijnhoven FJB, et al. An increase in physical activity after colorectal cancer surgery is associated with improved recovery of physical functioning: a prospective cohort study. *BMC Cancer.* 2017;17(1).

18. Antunes JMM, Ferreira RMP, Moreira-Goncalves D. Exercise Training as Therapy for Cancer-Induced Cardiac Cachexia. *Trends Mol Med.* 2018.

19. Arends J, Bachmann P, Baracos V, et al. ESPEN guidelines on nutrition in cancer patients. *Clin Nutr.* 2017;36(1):11-48.

20. Campbell KL, Winters-Stone KM, Wiskemann J, et al. Exercise Guidelines for Cancer Survivors. *Medicine & Science in Sports & Exercise.* 2019;51(11):2375-2390.

21. Cheema BS, Kilbreath SL, Fahey PP, Delaney GP, Atlantis E. Safety and efficacy of progressive resistance training in breast cancer: a systematic review and meta-analysis. *Breast Cancer Res Treat.* 2014;148(2):249-268.

22. Christensen JF, Jones LW, Andersen JL, Daugaard G, Rorth M, Hojman P. Muscle dysfunction in cancer patients. *Ann Oncol.* 2014;25(5):947-958.

23. Nilsen TS, Johansen SH, Thorsen L, Fairman CM, Wisløff T, Raastad T. Does Androgen Deprivation for Prostate Cancer Affect Normal Adaptation to Resistance Exercise? *International Journal of Environmental Research and Public Health.* 2022;19(7):3820.

24. Fairman CM, Nilsen TS, Newton RU, et al. Reporting of Resistance Training Dose, Adherence, and Tolerance in Exercise Oncology. *Med Sci Sports Exerc.* 2020;52(2):315-322.

25. Fairman CM, Hyde PN, Focht BC. Resistance training interventions across the cancer control continuum: a systematic review of the implementation of resistance training principles. *Br J Sports Med.* 2017;51(8):677-685.

26. Madzima TA, Ormsbee MJ, Schleicher EA, Moffatt RJ, Panton LB. Effects of Resistance Training and Protein Supplementation in Breast Cancer Survivors. *Med Sci Sports Exerc.* 2017;49(7):1283-1292.

27. Nilsen TS, Raastad T, Skovlund E, et al. Effects of strength training on body composition, physical functioning, and quality of life in prostate cancer patients during androgen deprivation therapy. *Acta Oncol.* 2015;54(10):1805-1813.

28. Schmitz KH, Troxel AB, Cheville A, et al. Physical Activity and Lymphedema (the PAL trial): assessing the safety of progressive strength training in breast cancer survivors. *Contemp Clin Trials.* 2009;30(3):233-245.

29. Focht BC, Lucas AR, Grainger E, et al. Effects of a Group-Mediated Exercise and Dietary Intervention in the Treatment of Prostate Cancer Patients Undergoing Androgen Deprivation Therapy: Results From the IDEA-P Trial. *Ann Behav Med.* 2018;52(5):412-428.

30. Bhasin S, Travison TG, Manini TM, et al. Sarcopenia Definition: The Position Statements of the Sarcopenia Definition and Outcomes Consortium. *Journal of the American Geriatrics Society.* 2020;68(7):1410-1418.

31. Kiss N, Loeliger J, Findlay M, et al. Clinical Oncology Society of Australia: Position statement on <scp>cancer‐related</scp> malnutrition and sarcopenia. *Nutrition & Dietetics.* 2020;77(4):416-425.

32. Roeland EJ, Bohlke K, Baracos VE, et al. Management of Cancer Cachexia: ASCO Guideline. *Journal of Clinical Oncology.* 2020;38(21):2438-2453.

33. Bland KA, Kouw IWK, Van Loon LJC, Zopf EM, Fairman CM. Exercise-Based Interventions to Counteract Skeletal Muscle Mass Loss in People with Cancer: Can We Overcome the Odds? *Sports Medicine.* 2022.

34. Chen Z, Zhang Y, Lu C, Zeng H, Schumann M, Cheng S. Supervised Physical Training Enhances Muscle Strength but Not Muscle Mass in Prostate Cancer Patients Undergoing Androgen Deprivation Therapy: A Systematic Review and Meta-Analysis. *Frontiers in Physiology.* 2019;10.

35. Bemben MG, Witten MS, Carter JM, Eliot KA, Knehans AW, Bemben DA. The effects of supplementation with creatine and protein on muscle strength following a traditional resistance training program in middle-aged and older men. *J Nutr Health Aging.* 2010;14(2):155-159.

36. Buford TW, Kreider RB, Stout JR, et al. International Society of Sports Nutrition position stand: creatine supplementation and exercise. *J Int Soc Sports Nutr.* 2007;4:6.

37. Fairman CM, Kendall KL, Hart NH, Taaffe DR, Galvão DA, Newton RU. The potential therapeutic effects of creatine supplementation on body composition and muscle function in cancer. *Critical Reviews in Oncology/Hematology.* 2019;133:46-57.

38. Gotshalk LA, Kraemer WJ, Mendonca MA, et al. Creatine supplementation improves muscular performance in older women. *Eur J Appl Physiol.* 2008;102(2):223-231.

39. Gotshalk LA, Volek JS, Staron RS, Denegar CR, Hagerman FC, Kraemer WJ. Creatine supplementation improves muscular performance in older men. *Med Sci Sports Exerc.* 2002;34(3):537-543.

40. Greenhaff PL, Bodin K, Soderlund K, Hultman E. Effect of oral creatine supplementation on skeletal muscle phosphocreatine resynthesis. *Am J Physiol.* 1994;266(5 Pt 1):E725-730.

41. Gualano B, Artioli GG, Poortmans JR, Lancha Junior AH. Exploring the therapeutic role of creatine supplementation. *Amino Acids.* 2010;38(1):31-44.

42. Aguiar AF, Januario RS, Junior RP, et al. Long-term creatine supplementation improves muscular performance during resistance training in older women. *Eur J Appl Physiol.* 2013;113(4):987-996.

43. Chilibeck PD, Kaviani M, Candow DG, Zello GA. Effect of creatine supplementation during resistance training on lean tissue mass and muscular strength in older adults: a meta-analysis. *Open Access J Sports Med.* 2017;8:213-226.

44. Forbes SC, Candow DG, Ostojic SM, Roberts MD, Chilibeck PD. Meta-Analysis Examining the Importance of Creatine Ingestion Strategies on Lean Tissue Mass and Strength in Older Adults. *Nutrients.* 2021;13(6):1912.

45. Gualano B, de Salles Painelli V, Roschel H, et al. Creatine supplementation does not impair kidney function in type 2 diabetic patients: a randomized, double-blind, placebo-controlled, clinical trial. *Eur J Appl Physiol.* 2011;111(5):749-756.

46. Gualano B, Macedo AR, Alves CR, et al. Creatine supplementation and resistance training in vulnerable older women: a randomized double-blind placebo-controlled clinical trial. *Exp Gerontol.* 2014;53:7-15.

47. Gualano B, Ugrinowitsch C, Novaes RB, et al. Effects of creatine supplementation on renal function: a randomized, double-blind, placebo-controlled clinical trial. *Eur J Appl Physiol.* 2008;103(1):33-40.

48. Hass CJ, Collins MA, Juncos JL. Resistance training with creatine monohydrate improves upper-body strength in patients with Parkinson disease: a randomized trial. *Neurorehabil Neural Repair.* 2007;21(2):107-115.

49. Olsen S, Aagaard P, Kadi F, et al. Creatine supplementation augments the increase in satellite cell and myonuclei number in human skeletal muscle induced by strength training. *J Physiol.* 2006;573(Pt 2):525-534.

50. Pinto CL, Botelho PB, Carneiro JA, Mota JF. Impact of creatine supplementation in combination with resistance training on lean mass in the elderly. *J Cachexia Sarcopenia Muscle.* 2016;7(4):413-421.

51. Stout JR, Sue Graves B, Cramer JT, et al. Effects of creatine supplementation on the onset of neuromuscular fatigue threshold and muscle strength in elderly men and women (64 - 86 years). *J Nutr Health Aging.* 2007;11(6):459-464.

52. Tarnopolsky MA, Mahoney DJ, Vajsar J, et al. Creatine monohydrate enhances strength and body composition in Duchenne muscular dystrophy. *Neurology.* 2004;62(10):1771-1777.

53. Walter MC, Lochmuller H, Reilich P, et al. Creatine monohydrate in muscular dystrophies: A double-blind, placebo-controlled clinical study. *Neurology.* 2000;54(9):1848-1850.

54. Writing Group for the NETiPDI, Kieburtz K, Tilley BC, et al. Effect of creatine monohydrate on clinical progression in patients with Parkinson disease: a randomized clinical trial. *JAMA.* 2015;313(6):584-593.

55. Chilibeck P, Kaviani M, Candow D, Zello GA. Effect of creatine supplementation during resistance training on lean tissue mass and muscular strength in older adults: a meta-analysis. *Open Access Journal of Sports Medicine.* 2017;Volume 8:213-226.

56. Candow DG, Forbes SC, Chilibeck PD, Cornish SM, Antonio J, Kreider RB. Effectiveness of Creatine Supplementation on Aging Muscle and Bone: Focus on Falls Prevention and Inflammation. *Journal of Clinical Medicine.* 2019;8(4):488.

57. Candow DG, Forbes SC, Kirk B, Duque G. Current Evidence and Possible Future Applications of Creatine Supplementation for Older Adults. *Nutrients.* 2021;13(3):745.

58. Wang S, Prizment A, Thyagarajan B, Blaes A. Cancer Treatment-Induced Accelerated Aging in Cancer Survivors: Biology and Assessment. *Cancers.* 2021;13(3):427.

59. Mallard J, Hucteau E, Hureau TJ, Pagano AF. Skeletal Muscle Deconditioning in Breast Cancer Patients Undergoing Chemotherapy: Current Knowledge and Insights From Other Cancers. *Front Cell Dev Biol.* 2021;9:719643.

60. Mijwel S, Cardinale DA, Norrbom J, et al. Exercise training during chemotherapy preserves skeletal muscle fiber area, capillarization, and mitochondrial content in patients with breast cancer. *The FASEB Journal.* 2018;32(10):5495-5505.

61. Moreira-Pais A, Ferreira R, Gil Da Costa R. Platinum-induced muscle wasting in cancer chemotherapy: Mechanisms and potential targets for therapeutic intervention. *Life Sciences.* 2018;208:1-9.

62. Sturgeon KM, Mathis KM, Rogers CJ, Schmitz KH, Waning DL. Cancer- and Chemotherapy-Induced Musculoskeletal Degradation. *JBMR Plus.* 2019;3(3):e10187.

63. Hiensch AE, Bolam KA, Mijwel S, et al. Doxorubicin‐induced skeletal muscle atrophy: Elucidating the underlying molecular pathways. *Acta Physiologica.* 2020;229(2).

64. Gorini S, De Angelis A, Berrino L, Malara N, Rosano G, Ferraro E. Chemotherapeutic Drugs and Mitochondrial Dysfunction: Focus on Doxorubicin, Trastuzumab, and Sunitinib. *Oxidative Medicine and Cellular Longevity.* 2018;2018:1-15.

65. Kavazis AN, Smuder AJ, Powers SK. Effects of short-term endurance exercise training on acute doxorubicin-induced FoxO transcription in cardiac and skeletal muscle. *J Appl Physiol (1985).* 2014;117(3):223-230.

66. Gilliam LAA, St. Clair DK. Chemotherapy-Induced Weakness and Fatigue in Skeletal Muscle: The Role of Oxidative Stress. *Antioxidants & Redox Signaling.* 2011;15(9):2543-2563.

67. Barreto R, Waning DL, Gao H, Liu Y, Zimmers TA, Bonetto A. Chemotherapy-related cachexia is associated with mitochondrial depletion and the activation of ERK1/2 and p38 MAPKs. *Oncotarget.* 2016;7(28):43442-43460.

68. Gilliam LAA, Fisher-Wellman KH, Lin C-T, Maples JM, Cathey BL, Neufer PD. The anticancer agent doxorubicin disrupts mitochondrial energy metabolism and redox balance in skeletal muscle. *Free Radical Biology and Medicine.* 2013;65:988-996.

69. Doerr V, Montalvo RN, Kwon OS, et al. Prevention of Doxorubicin-Induced Autophagy Attenuates Oxidative Stress and Skeletal Muscle Dysfunction. *Antioxidants.* 2020;9(3):263.

70. Smuder AJ, Kavazis AN, Min K, Powers SK. Exercise protects against doxorubicin-induced oxidative stress and proteolysis in skeletal muscle. *J Appl Physiol (1985).* 2011;110(4):935-942.

71. Bonetto A, Aydogdu T, Jin X, et al. JAK/STAT3 pathway inhibition blocks skeletal muscle wasting downstream of IL-6 and in experimental cancer cachexia. *American Journal of Physiology-Endocrinology and Metabolism.* 2012;303(3):E410-E421.

72. Damrauer JS, Stadler ME, Acharyya S, Baldwin AS, Couch ME, Guttridge DC. Chemotherapy-induced muscle wasting: association with NF-κB and cancer cachexia. *European Journal of Translational Myology.* 2018;28(2).

73. Rosa-Caldwell ME, Fix DK, Washington TA, Greene NP. Muscle alterations in the development and progression of cancer-induced muscle atrophy: a review. *J Appl Physiol (1985).* 2020;128(1):25-41.

74. Vanderveen BN, Murphy EA, Carson JA. The Impact of Immune Cells on the Skeletal Muscle Microenvironment During Cancer Cachexia. *Frontiers in Physiology.* 2020;11.

75. Webster JM, Kempen LJAP, Hardy RS, Langen RCJ. Inflammation and Skeletal Muscle Wasting During Cachexia. *Frontiers in Physiology.* 2020;11.

76. Clifford B, Koizumi S, Wewege M, et al. The effect of resistance training on body composition in cancer survivors during treatment and throughout survivorship: a systematic review and meta-analysis. *Sports Med.* 2021;In Review.

77. Bauer J, Morley JE, Schols AMWJ, et al. Sarcopenia: A Time for Action. An SCWD Position Paper. *Journal of Cachexia, Sarcopenia and Muscle.* 2019;10(5):956-961.

78. Wing RR. Long-term effects of a lifestyle intervention on weight and cardiovascular risk factors in individuals with type 2 diabetes mellitus: four-year results of the Look AHEAD trial. *Arch Intern Med.*170(17):1566-1575.

79. Rejeski WJ, Focht BC, Messier SP, Morgan T, Pahor M, Penninx B. Obese, older adults with knee osteoarthritis: weight loss, exercise, and quality of life. *Health Psychol.* 2002;21(5):419-426.

80. Focht BC, Rejeski WJ, Ambrosius WT, Katula JA, Messier SP. Exercise, self-efficacy, and mobility performance in overweight and obese older adults with knee osteoarthritis. *Arthritis Rheum.* 2005;53(5):659-665.

81. Rejeski WJ, Brubaker PH, Goff DC, Jr., et al. Translating Weight Loss and Physical Activity Programs Into the Community to Preserve Mobility in Older, Obese Adults in Poor Cardiovascular Health. *Arch Intern Med.* 2011.

82. Fairman CM, Kendall KL, Newton RU, et al. Examining the effects of creatine supplementation in augmenting adaptations to resistance training in patients with prostate cancer undergoing androgen deprivation therapy: a randomised, double-blind, placebo-controlled trial. *BMJ Open.* 2019;9(9):e030080.

83. Candow DG, Little JP, Chilibeck PD, et al. Low-dose creatine combined with protein during resistance training in older men. *Med Sci Sports Exerc.* 2008;40(9):1645-1652.

84. Candow DG, Chilibeck PD, Gordon J, et al. Effect of 12 months of creatine supplementation and whole-body resistance training on measures of bone, muscle and strength in older males. *Nutrition and Health.* 2020:026010602097524.

85. Vanderveen BN, Fix DK, Counts BR, Carson JA. The Effect of Wheel Exercise on Functional Indices of Cachexia in Tumor-bearing Mice. *Med Sci Sports Exerc.* 2020;52(11):2320-2330.

86. Vanderveen BN, Fix DK, Montalvo RN, et al. The regulation of skeletal muscle fatigability and mitochondrial function by chronically elevated interleukin-6. *Experimental Physiology.* 2019;104(3):385-397.

87. Vanderveen BN, Sougiannis AT, Velazquez KT, Carson JA, Fan D, Murphy EA. The Acute Effects of 5 Fluorouracil on Skeletal Muscle Resident and Infiltrating Immune Cells in Mice. *Frontiers in Physiology.* 2020;11.

88. Aging NIo. NIH Stage Model for Behavioral Intervention Development. <https://www.nia.nih.gov/research/dbsr/nih-stage-model-behavioral-intervention-development>. Updated Feb 8th, 2022. Accessed Feb 1st, 2022.

89. Health NNCfCaI. Pilot Studies: Common Uses and Misuses. <https://www.nccih.nih.gov/grants/pilot-studies-common-uses-and-misuses>. Updated Feb 8th, 2022. Accessed Feb 6th, 2022.

90. Courneya KS, Segal RJ, Mackey JR, et al. Effects of Aerobic and Resistance Exercise in Breast Cancer Patients Receiving Adjuvant Chemotherapy: A Multicenter Randomized Controlled Trial. *Journal of Clinical Oncology.* 2007;25(28):4396-4404.

91. Galvao DA, Spry N, Denham J, et al. A multicentre year-long randomised controlled trial of exercise training targeting physical functioning in men with prostate cancer previously treated with androgen suppression and radiation from TROG 03.04 RADAR. *Eur Urol.* 2014;65(5):856-864.

92. Galvao DA, Taaffe DR, Spry N, Joseph D, Newton RU. Combined resistance and aerobic exercise program reverses muscle loss in men undergoing androgen suppression therapy for prostate cancer without bone metastases: a randomized controlled trial. *J Clin Oncol.* 2010;28(2):340-347.

93. Scott JM, Iyengar NM, Nilsen TS, et al. Feasibility, safety, and efficacy of aerobic training in pretreated patients with metastatic breast cancer: A randomized controlled trial. *Cancer.* 2018;124(12):2552-2560.

94. Travier N, Velthuis MJ, Steins Bisschop CN, et al. Effects of an 18-week exercise programme started early during breast cancer treatment: a randomised controlled trial. *BMC Med.* 2015;13:121.

95. Mauro J, Mathews KB, Sredzinski ES. Effect of a Smart Pill Bottle and Pharmacist Intervention on Medication Adherence in Patients with Multiple Myeloma New to Lenalidomide Therapy. *Journal of Managed Care & Specialty Pharmacy.* 2019;25(11):1244-1254.

96. Park HR, Kang HS, Kim SH, Singh-Carlson S. Effect of a Smart Pill Bottle Reminder Intervention on Medication Adherence, Self-efficacy, and Depression in Breast Cancer Survivors. *Cancer Nurs.* 2021.

97. Toscos T, Drouin M, Pater JA, et al. Medication adherence for atrial fibrillation patients: triangulating measures from a smart pill bottle, e-prescribing software, and patient communication through the electronic health record. *JAMIA Open.* 2020;3(2):233-242.

98. Ramirez-Velez R, Perez-Sousa MA, Venegas-Sanabria LC, et al. Normative Values for the Short Physical Performance Battery (SPPB) and Their Association With Anthropometric Variables in Older Colombian Adults. The SABE Study, 2015. *Front Med (Lausanne).* 2020;7:52.

99. Craig CL, Marshall AL, Sjostrom M, et al. International physical activity questionnaire: 12-country reliability and validity. *Med Sci Sports Exerc.* 2003;35(8):1381-1395.

100. Beaudart C, Biver E, Reginster J-Y, et al. Validation of the SarQoL®, a specific health-related quality of life questionnaire for Sarcopenia. *Journal of Cachexia, Sarcopenia and Muscle.* 2017;8(2):238-244.

101. Leon AC, Davis LL, Kraemer HC. The role and interpretation of pilot studies in clinical research. *Journal of Psychiatric Research.* 2011;45(5):626-629.

102. Whitehead AL, Julious SA, Cooper CL, Campbell MJ. Estimating the sample size for a pilot randomised trial to minimise the overall trial sample size for the external pilot and main trial for a continuous outcome variable. *Statistical Methods in Medical Research.* 2016;25(3):1057-1073.

103. Julious SA. Sample size of 12 per group rule of thumb for a pilot study. *Pharmaceutical Statistics.* 2005;4(4):287-291.

**G. APPENDIX**

*Attach any additional information pertinent to the application, such as surveys or questionnaires, etc.*
