# Supplementary figures and images for: Examining the feasibility and preliminary effects of resistance exercise training and creatine supplementation in individuals treated for colorectal cancer
Source: PLoS One. 2026 Jul 15;21(7):e0353630. doi: 10.1371/journal.pone.0353630 (PMC13372120; doi:10.1371/journal.pone.0353630)

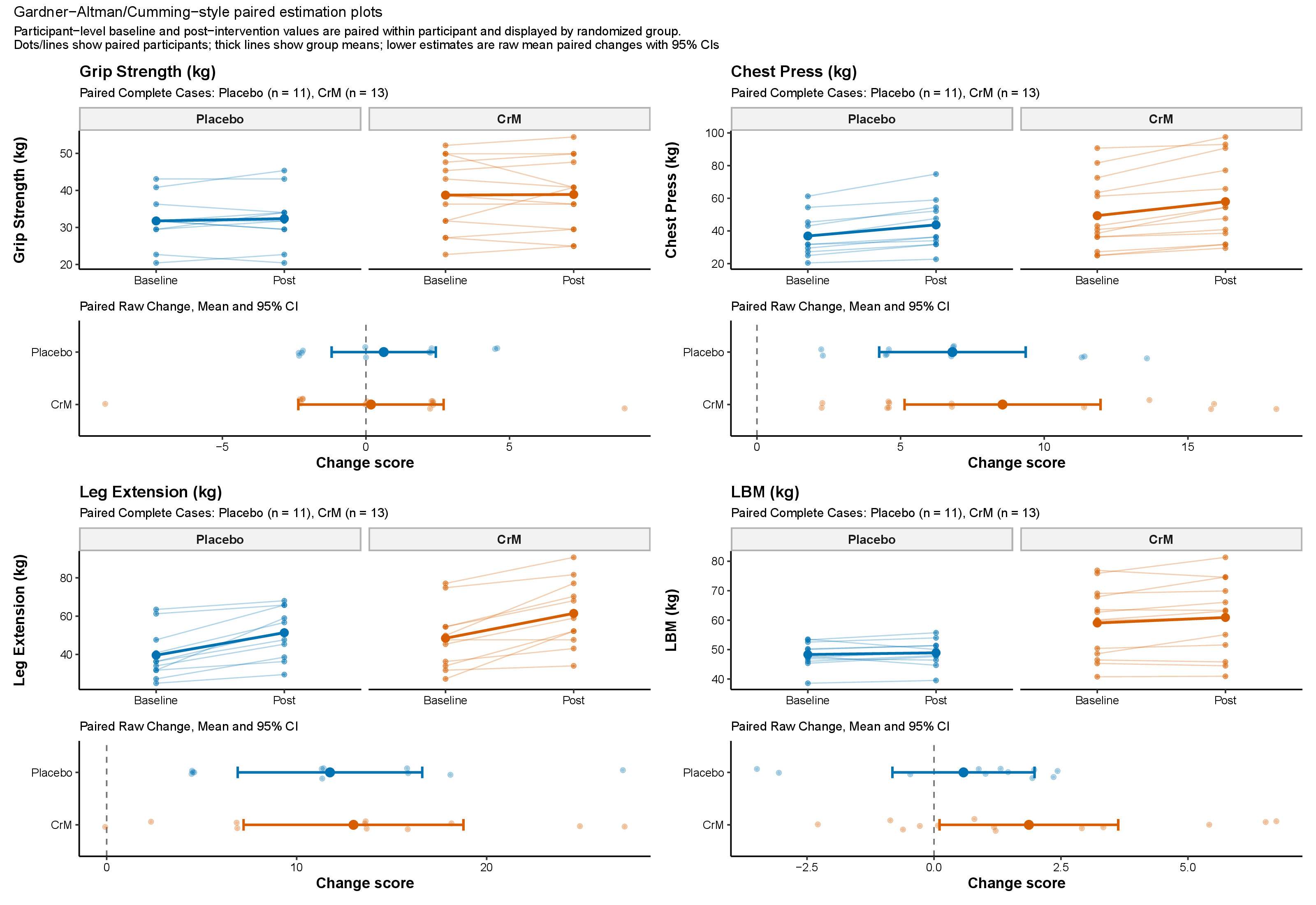

Supplement: S5 File — (TIFF) [file pone.0353630.s005.tiff]
