# Supplementary material for: Examining the feasibility and preliminary effects of resistance exercise training and creatine supplementation in individuals treated for colorectal cancer
Source: PLoS One. 2026 Jul 15;21(7):e0353630. doi: 10.1371/journal.pone.0353630 (PMC13372120; doi:10.1371/journal.pone.0353630)
Supplement: Attachment — Submitted filename: Response to Reviewers.docx [file pone.0353630.s007.docx]

**When submitting your revision, we need you to address these additional requirements.**

**1.Please ensure that your manuscript meets PLOS ONE's style requirements, including those for file naming. The PLOS ONE style templates can be found at**

**https://journals.plos.org/plosone/s/file?id=wjVg/PLOSOne_formatting_sample_main_body.pdf and**

[**https://journals.plos.org/plosone/s/file?id=ba62/PLOSOne_formatting_sample_title_authors_affiliations.pdf**](https://journals.plos.org/plosone/s/file?id=ba62/PLOSOne_formatting_sample_title_authors_affiliations.pdf)

We have verified that the revised manuscript adheres to the Style Templates.

**2. Thank you for stating the following in the Competing Interests section:**

**“DGC has conducted industry-sponsored research involving creatine supplementation and has received creatine donations for scientific studies and travel support for presentations related to creatine supplementation at scientific conferences. He serves on the Scientific Advisory Board for AlzChem (a manufacturer of creatine) and has acted as an expert witness and consultant in legal cases involving creatine supplementation. CMF reports consulting activities related to exercise oncology and resistance training for Inspire Exercise Medicine and has received speaker honoraria for AstraZeneca. The remaining authors declare that they have no relevant financial or non-financial interests to disclose. The results of this study are presented clearly, honestly, and without fabrication, falsification, or inappropriate data manipulation.” We note that one or more of the authors are employed by a commercial company: AlzChem**

**1. Please provide an amended Funding Statement declaring this commercial affiliation, as well as a statement regarding the Role of Funders in your study. If the funding organization did not play a role in the study design, data collection and analysis, decision to publish, or preparation of the manuscript and only provided financial support in the form of authors' salaries and/or research materials, please review your statements relating to the author contributions, and ensure you have specifically and accurately indicated the role(s) that these authors had in your study. You can update author roles in the Author Contributions section of the online submission form.**

**Please also include the following statement within your amended Funding Statement.**

**“The funder provided support in the form of salaries for authors [insert relevant initials], but did not have any additional role in the study design, data collection and analysis, decision to publish, or preparation of the manuscript. The specific roles of these authors are articulated in the ‘author contributions’ section.” If your commercial affiliation did play a role in your study, please state and explain this role within your updated Funding Statement.**

DGC is not employed by AlzChem. He serves on AlzChem's Scientific Advisory Board and is a paid consultant, both of which are disclosed in the Competing Interests statement. AlzChem's only contribution to this study was the in-kind provision of the creatine and matched placebo; it had no role in the study design, data collection and analysis, decision to publish, or preparation of the manuscript. We have amended the Funding Statement and Competing Interests accordingly.

**Revised Funding Statement**

This work was supported by the National Cancer Institute of the National Institutes of Health under Award Number R21CA281951-01. The content is solely the responsibility of the authors and does not necessarily represent the official views of the National Institutes of Health. Creatine monohydrate (Creapure®) and the matched placebo were provided by AlzChem Trostberg GmbH (Trostberg, Germany). This publication was also supported by the National Institute Of General Medical Sciences of the National Institutes of Health under Award Number P20GM155896 through the Center for Clinical & Translational Research at University of South Carolina (ClinTrUSC). The content is solely the responsibility of the authors and does not necessarily represent the official views of the National Institutes of Health. The funders had no role in the study design, data collection and analysis, decision to publish, or preparation of the manuscript.

**2. Please also provide an updated Competing Interests Statement declaring this commercial affiliation along with any other relevant declarations relating to employment, consultancy, patents, products in development, or marketed products, etc.  Within your Competing Interests Statement, please confirm that this commercial affiliation does not alter your adherence to all PLOS ONE policies on sharing data and materials by including the following statement: "This does not alter our adherence to  PLOS ONE policies on sharing data and materials.” (as detailed online in our guide for authors http://journals.plos.org/plosone/s/competing-interests) . If this adherence statement is not accurate and  there are restrictions on sharing of data and/or materials, please state these. Please note that we cannot proceed with consideration of your article until this information has been declared.**

We confirm that there are no restrictions on the sharing of data or materials associated with this study. We have added the requested statement verbatim to the Competing Interests section

**Revised Competing Interests Statement**

DGC has conducted industry-sponsored research involving creatine supplementation and has received creatine donations for scientific studies and travel support for presentations related to creatine supplementation at scientific conferences. He serves on the Scientific Advisory Boards of AlzChem, Create, and Lovate, and is a paid consultant to AlzChem (companies that manufacture creatine). AlzChem provided the creatine monohydrate and matched placebo used in this study but had no role in the study design, data collection and analysis, decision to publish, or preparation of the manuscript. CMF reports consulting activities related to exercise oncology and resistance training for Inspire Exercise Medicine and has received speaker honoraria from AstraZeneca. The remaining authors declare that they have no relevant financial or non-financial interests to disclose. This does not alter our adherence to PLOS ONE policies on sharing data and materials. The results of this study are presented clearly, honestly, and without fabrication, falsification, or inappropriate data manipulation.

**Please include both an updated Funding Statement and Competing Interests Statement in your cover letter. We will change the online submission form on your behalf.**

As outlined above, we have amended both the Funding Statement and Competing Interests Statement and have included them in the cover letter with the resubmission.

**3. When completing the data availability statement of the submission form, you indicated that you will make your data available on acceptance. We strongly recommend all authors decide on a data sharing plan before acceptance, as the process can be lengthy and hold up publication timelines. Please note that, though access restrictions are acceptable now, your entire data will need to be made freely accessible if your manuscript is accepted for publication. This policy applies to all data except where public deposition would breach compliance with the protocol approved by your research ethics board. If you are unable to adhere to our open data policy, please kindly revise your statement to explain your reasoning and we will seek the editor's input on an exemption. Please be assured that, once you have provided your new statement, the assessment of your exemption will not hold up the peer review process.**

Our Data availability statement does not say the data will be available upon acceptance. The data is publicly available, and the data availability statement reflects this:

**“**All data reported in this manuscript are publicly available. Deidentified participant-level data, metadata (data dictionary), and analysis code underlying the study findings are available in a public repository hosted on GitHub: https://github.com/zhangtiejun310/R21_Resistance-Exercise-Training-and-Creatine-Supplementation. The repository contains the dataset used for analysis, accompanying documentation, and scripts necessary to reproduce the results.”

**4. Please upload a copy of Figure 1 and 2, to which you refer in your text on page 7 and 8. If the figure is no longer to be included as part of the submission please remove all reference to it within the text.**

We have included these figures in the submission

**5. Please include a copy of Table 3-5 which you refer to in your text on page 16 and 18.**

These 3 Tables are tables in Supplementary File S2. For example “S2, Table 3” refers to the third table in Supplementary File S2.

**6. Please upload a copy of your study protocol that was approved by your ethics committee/IRB as a Supporting Information file. By the study protocol, we mean the complete and detailed plan for the conduct and analysis of the trial approved by the ethics committee/IRB. Please send this in the original language. If this is in a language other than English, please also provide a translation. [https://journals.plos.org/plosone/s/submission-guidelines#loc-guidelines-for-specific-study-types**

We have updated file S3 for the resubmission to be the protocol rather than the IRB approval letter.

**MAJOR COMMENTS:**

**1. The authors describe several important changes to the study protocol (eligibility criteria, outcome assessments, intervention delivery, and supplementation dose) that deviate from the original ClinicalTrials.gov registration (NCT06420726). Could you please clarify why the trial record was not updated to reflect these changes? Timely updating of the registry entry is essential for transparency and adherence to best practices in clinical trial reporting. Also, why is the entry registration in "Recruiting" state?**

We thank the reviewer for raising this point. We agree that timely updating of the trial registry is essential for transparency and adherence to best practice in clinical trial reporting, and we apologize that the ClinicalTrials.gov record (NCT06420726) was not kept current with the protocol amendments at the time of submission. As amendments were made over the course of the trial, the corresponding updates were not entered into the ClinicalTrials.gov record, and this lapse was not caught by the study team. As a result, the public record continued to display the original protocol and an active recruitment status. This was an administrative oversight on our part rather than an intentional omission. We have now updated the record in full. The registry has been revised to reflect each of the amendments described in the manuscript.

Regarding the reviewer's second question, the "Recruiting" status was a direct consequence of the record not having been updated; it did not reflect the true state of the trial, which completed data collection in June 2025. The overall status has now been changed to "Completed," and the actual enrollment (n = 27) and study completion date have been entered.

Finally, we note that, independent of the registry, every protocol amendment and its rationale were disclosed in the submitted manuscript. These are described in the "Changes to Study Protocol" section and itemized against the original protocol language in S2 (Table 1). We hope that the corrected registry entry, together with this in-manuscript documentation, provides complete transparency regarding the evolution of the protocol.

**2. The structure of the manuscript, especially the METHODS section, needs to be revised. All subsections should be organized in strict order as recommended by CONSORT guidelines for pilot and feasibility studies (Trial design, Participants, Intervention Procedures, Outcomes, Sample Size, Statistical Analysis) as informed in the submission guidelines of PLoS One. Cf, https://pubmed.ncbi.nlm.nih.gov/31608150/ or https://www.equator-network.org/wp-content/uploads/2016/10/CONSORT-extension-Pilot-and-Feasibility-Trials-Checklist.doc  Replace the checklist with the correct CONSORT extension for pilot and feasibility studies (supplementary file 1).**

We thank the reviewer. We have restructured the Methods section to follow the order recommended by the CONSORT extension for randomised pilot and feasibility trials, with the subsections now presented as Trial design, Participants, Intervention Procedures, Outcomes, Sample Size, and Statistical Analysis. We have also replaced the checklist in Supplementary File 1 with the correct CONSORT 2010 extension to randomised pilot and feasibility trials checklist, completed for this manuscript and cross-referenced to the relevant sections.

**3. Could the authors confirm whether the estimated variable of body composition reported in the manuscript is fat-free mass (lean body mass) or lean soft tissue? As the authors are aware, there are relevant differences between the terms fat-free mass (FFM), lean soft tissue (LST) and skeletal muscle mass (SMM). As recommended recently by Heymsfield et al. (2024), 'to avoid confusion and improve scientific rigor, FFM is the preferred term for this body component (lean body mass), which is often measured with techniques using 2-compartment models such as TBW, body density, BIA, DXA, and 3D optical imaging.' Cf,** [**https://pubmed.ncbi.nlm.nih.gov/39510253/**](https://pubmed.ncbi.nlm.nih.gov/39510253/) **These terms might be mistakenly interchanged considering the DXA principle and measurements. Please refer to** **https://pubmed.ncbi.nlm.nih.gov/29786955/ and** [**https://dexalytics.com/news/lean-soft-tissue-or-fat-free-mass/**](https://dexalytics.com/news/lean-soft-tissue-or-fat-free-mass/)

We confirm that the DXA-derived values reported are lean soft tissue (LST), measured with the Hologic Horizon A: whole-body lean soft tissue and appendicular lean soft tissue (ALM), both of which exclude bone mineral content.

**4. In addition, did the authors correct data for fat-free adipose tissue (FFAT) before the analysis? If not, the analyses must be performed again. When using DXA, this is very important considering that the prevalence of low muscle mass and functionality in the US population is strongly affected by FFAT. Cf, https://pubmed.ncbi.nlm.nih.gov/27507068/,** [**https://pubmed.ncbi.nlm.nih.gov/29915252/**](https://pubmed.ncbi.nlm.nih.gov/29915252/)

We thank the reviewer for raising the influence of fat-free adipose tissue (FFAT) on DXA-derived lean mass. We would respectfully note that the references cited are cross-sectional studies concerned with the prevalence and classification of low muscle mass, in which uncorrected appendicular lean mass is inflated at higher adiposity and can shift individuals across diagnostic cut-points. The present study does not estimate prevalence or classify participants against muscle-mass thresholds; it reports baseline-adjusted between-group differences in continuous lean mass within a randomized trial.

The relevance of the FFAT correction to this longitudinal, between-group context was examined directly by Moore et al. (2024)[1], who applied the same correction formula[2] across 226 participants in five exercise and nutrition trials. They reported that corrected and uncorrected lean-mass change scores diverged substantially only in cases of large body-mass loss (>10%, e.g., following bariatric surgery); the only other condition reaching significance was modest recomposition, where the difference between methods was nonetheless trivial in magnitude (on the order of 0.1 kg) and *did not alter study conclusions.* *The authors concluded that for most populations and intervention types DXA-derived values are accurate and the correction may not be necessary.* Our trial delivered resistance training with creatine, and no participant approached the degree of body-mass change at which the correction becomes consequential (the largest body-mass change observed was approximately 5%). On this basis, the FFAT correction would not be expected to meaningfully affect the between-group estimates reported here, and we do not believe re-analysis is warranted for the present design. We appreciate the reviewer's attention to this issue, and we hope the reviewer agrees that re-analysis is not warranted given the design and population of this trial.

**5. Add the CV% of the instruments/technician to know the variability of all measures.**

We have added precision and reliability information for the secondary outcome measures to the Methods (Secondary Outcomes). For DXA, we now report instrument precision from our own lumbar spine phantom quality-control record and note that all scans were acquired and analyzed by a single trained technician, minimizing operator variability. Formal in-house test-retest/precision studies were not conducted within this feasibility trial. Where relevant, in lieu of in-house values, we report previously published precision and reliability for the same devices and instruments used in our trial.

**6. I agree with the reviewers regarding the statistical analysis. Please run a robust ANCOVA. Cf,** [**https://pubmed.ncbi.nlm.nih.gov/18652737/**](https://pubmed.ncbi.nlm.nih.gov/18652737/)

We agree that, given the small pilot sample and the exploratory nature of the secondary outcome analyses, it is important to evaluate whether the ANCOVA results depend on conventional model-based standard errors or other distributional assumptions. In response, we conducted additional sensitivity analyses for all exploratory secondary outcomes. For each outcome, we retained the same baseline-adjusted ANCOVA specification used in the manuscript, with the post-intervention value as the dependent variable, randomized treatment group as the predictor of interest, and the corresponding baseline value as a covariate. We also reviewed model diagnostics, including residual patterns, influential observations, and the plausibility of the baseline-by-treatment relationship. Given the small sample size, these diagnostics were interpreted descriptively rather than as formal assumption tests.

We specifically considered the robust ANCOVA approach recommended by the reviewer and described by Wilcox, which uses a running interval smoother and compares medians or 20% trimmed means with bootstrap-based inference. This approach is attractive because it relaxes several assumptions of conventional ANCOVA, including parametric assumptions about the regression lines and the assumption of parallel slopes. However, the method estimates group differences locally at covariate evaluation points, and the available implementation returns group-specific sample sizes at those evaluation points, trimmed mean differences, standard errors, confidence intervals, and p values. In our pilot dataset, the small number of participants per arm and sparse overlap in baseline covariate values led to too few observations within several local smoothing windows after trimming. As a result, the running-interval smoother and bootstrap procedures produced non-estimable or highly unstable results for several outcomes. Thus, the limitation was not the conceptual appropriateness of robust ANCOVA, but the feasibility and stability of this particular nonparametric/ local-smoother implementation in our small pilot sample.

As a feasible alternative, we therefore refit the original baseline-adjusted ANCOVA models and recalculated inference for the treatment-group coefficient using heteroscedasticity-consistent HC3 covariance estimates. The HC3 estimator is specifically designed to improve finite-sample performance of heteroscedasticity-consistent inference and has been shown to perform better than earlier heteroscedasticity-consistent estimators in small samples. This approach preserves the same estimand as the original ANCOVA while relaxing the homoscedasticity assumption for the standard errors, confidence intervals, and p values. The HC3 analyses did not materially alter the interpretation of the findings. The direction and magnitude of the estimated treatment effects were largely unchanged, and no between-group comparison reached statistical significance using HC3 inference.

We have revised the Statistical Analysis section to describe the additional ANCOVA diagnostic checks and the HC3 robust-variance sensitivity analyses. We have also added the HC3 standard errors, 95% confidence intervals, and p values to S2 Table 6 and revised the Results and Discussion to emphasize that all secondary outcome analyses are exploratory.

**7. Generate figures with estimation plots to display the repeated measures data across time points (show individual data/responses at baseline and after creatine supplementation). Cumming or Gardner-Altman estimation plots are recommended.**

We agree that estimation plots provide a clearer and more transparent display of individual repeated-measures responses than summary tables alone, particularly in a small pilot trial. We have added Cumming-style paired estimation plots. Each plot displays participant-level baseline and post-intervention values, with each participant paired across time and displayed separately by randomized group. The plots also show group mean trajectories and raw paired-change estimates with bootstrap 95% confidence intervals to aid visual interpretation of individual response patterns. As the study included 28 analyzed secondary outcomes, the full set of paired estimation plots for all 28 outcomes is provided in the Supplementary materials (S5).

**Minor details:**

1. **RE, "DEXA" with "DXA". As recommended by the International Society for Clinical Densitometry (ISCD), DXA is the preferred abbreviation. Revise through the manuscript. Cf, https://iscd.org/official-positions-2023/ (nomenclature) and** [**https://pubmed.ncbi.nlm.nih.gov/27020004/**](https://pubmed.ncbi.nlm.nih.gov/27020004/)

This has been Revised.

2. **Revise the manuscript for typos. For example, all references are incorrectly placed after periods (e.g., .(1-3)).**

We have corrected the placement of all in-text citations so that they now precede the terminal punctuation rather than follow it, and we have proofread the full manuscript to correct typographical errors throughout.

**Reviewer #1: General assessment
This manuscript presents a pilot randomized controlled trial evaluating the feasibility, safety, and acceptability of a 10-week hybrid resistance exercise training (RET) program with or without creatine monohydrate supplementation in individuals previously treated for colorectal cancer. The topic is relevant and important in the context of exercise oncology and cancer survivorship, particularly given the prevalence of sarcopenia, functional decline, and reduced quality of life following chemotherapy. The manuscript is generally well written, the intervention is clearly described, and the feasibility outcomes are appropriate for a pilot trial. The inclusion of adherence, retention, acceptability, and adverse events is a strength, as is the hybrid delivery model, which has important clinical applicability. However, there are several methodological and statistical issues that need clarification, and the interpretation of secondary outcomes should be more cautious given that the study is not powered for efficacy outcomes.**

We thank the reviewer for their careful reading of the manuscript and for the positive assessment of the trial's relevance, design, and feasibility framework. We appreciate the recognition of the hybrid delivery model and the feasibility outcomes as strengths. We have addressed each of the reviewer's specific comments in detail below and believe the manuscript is improved as a result.

**Pilot study vs efficacy interpretation
The stated primary aim is feasibility, acceptability, and safety, which is appropriate for a pilot trial. However, in several sections (Results and Discussion), the manuscript interprets changes in strength, body composition, and physical function as meaningful intervention effects. Since both groups received RET and there was no non-exercise control group, the study cannot determine the efficacy of RET itself, nor definitively evaluate the additive effect of creatine given the small sample size. The authors should emphasize that secondary outcomes are exploratory and avoid causal language suggesting effectiveness.**

We fully agree that, as a feasibility pilot with no non-exercise control group and a small sample, the study cannot determine the efficacy of RET or the additive effect of creatine. We appreciate the opportunity to clarify this, and we have revised the Discussion to frame the secondary outcomes as exploratory and to remove the causal language that implied effectiveness. The updated text is below.

*“These secondary outcomes were exploratory and hypothesis-generating as the trial was not designed or powered to evaluate efficacy. Further, in the absence of a non-exercise control group, the observed changes cannot be attributed to RET specifically. We were able to obtain these measures in nearly all participants, demonstrating that the assessment battery was feasible to administer. Adjusted analyses showed no meaningful differences between groups for LST, strength, or physical function, and wide variability across participants limits interpretation. Both groups showed modest within-group changes in muscular strength and SPPB scores over the intervention period. These within-group changes are directionally consistent with the broader literature on RET in individuals with cancer [96-101], and the lack of additive effects of CrM are similar to our previous research in prostate cancer [41]. The changes observed across both groups are encouraging given the well-documented declines in these outcomes following chemotherapy [5-9], and support the rationale for a future adequately powered, controlled trial to test whether RET counteracts treatment-related muscle decline and its associated mortality risk [10-12]. As this feasibility study was not powered for between-group comparisons, null effects should be interpreted cautiously rather than as evidence of no benefit of CrM supplementation.”*

**Protocol modifications
The manuscript reports several modifications to the original protocol. These are substantial changes and may affect internal validity and comparability with the original protocol. The authors should clarify:
When these changes were made (before vs after recruitment began)
Whether these changes were updated in the trial registration
Whether these changes were approved by the IRB
How these changes may affect interpretation of feasibility and secondary outcomes**

We have clarified each of these points. Supplementary File S2 has been updated to specify when each modification was made relative to the start of recruitment. We have addressed each point below:

**Timing.** Most modifications, including the familiarization schedule, the substitution of leg extension for leg press, the session-time offerings, the hybrid delivery format, and the creatine dosing protocol, were finalized before recruitment commenced and therefore applied uniformly to all enrolled participants. Two modifications were made after recruitment began, in response to slow accrual: the broadening of eligibility (removal of confirmed sarcopenia and the ≥12-month post-treatment requirement, and addition of prior chemotherapy) and the removal of stratified randomization. The timing of each is now documented in S2.

**Trial registration.** These modifications were not updated in the ClinicalTrials.gov record during the trial, which was an administrative oversight on our part (see response to editor above). The registration (NCT06420726) has now been fully updated to reflect all modifications and the completed status.

**IRB approval.** All modifications were reviewed and approved by the University of South Carolina IRB (Pro00127362).

**Effect on interpretation.** As the design, delivery, and dosing changes were finalized before enrollment, they define the protocol that was actually tested and were applied uniformly across participants, so they do not introduce within-trial inconsistency, although they do mean the tested protocol differs from the originally registered design. The two post-recruitment changes carry the principal interpretive implications. The eligibility broadening is itself an informative feasibility finding, indicating that the original criteria were too restrictive for timely accrual, but it also produced a more heterogeneous and higher-functioning sample, which we discuss as a source of selection bias and a limit on generalizability. The removal of stratification increased the potential for baseline imbalance, which we addressed using baseline-adjusted ANCOVA. For these reasons, and because the trial was not powered for between-group comparisons, the secondary outcomes are interpreted as exploratory and hypothesis-generating.

**Recruitment and selection bias
The recruitment rate was very low (6.6%), and participants who enrolled appear relatively high functioning at baseline (SPPB ~10–11 out of 12). This suggests potential selection bias toward more motivated and physically capable participants, which limits generalizability to more vulnerable cancer survivors (e.g., sarcopenic, frail, recently treated patients).
This limitation should be discussed more explicitly.**

We agree that the low recruitment rate and the relatively high baseline function of those enrolled indicate likely selection bias and limit generalizability to more vulnerable survivors. We have revised the limitations to address this explicitly, noting the potential for selection or volunteer bias and the reduced generalizability to sarcopenic, frail, or more recently treated patients. The revised text is below.

*“Our study has several limitations worthy of note. The small sample size and narrow sample characteristics limit the generalizability of our findings. The low recruitment rate (6.6%) and the relatively high baseline physical function of those enrolled (SPPB approximately 10-11 of 12) raise the possibility of selection or volunteer bias, whereby those who enrolled may have been more motivated and physically capable than the broader colorectal cancer population. Our findings may therefore not generalize to more vulnerable individuals, including those who are sarcopenic, frail, or more recently treated.”*

**Body composition measurement and creatine. Creatine supplementation may increase total body water, which can artificially increase lean body mass measured by DEXA. The authors mention this limitation briefly, but this is a critical methodological issue, particularly because lean mass is one of the main outcomes. The authors should: Expand this limitation. Clarify that changes in lean mass may reflect fluid shifts rather than true hypertrophy. Consider this when interpreting (discussion) body composition results**

We appreciate the reviewer emphasizing this point, and we agree it is an important methodological consideration given that lean mass is a key outcome. We would respectfully note that this limitation was explicitly stated in our original submission, where we acknowledged that CrM supplementation may increase total body water and that, without a measure of total body water, we could not be confident whether changes in DXA-derived lean mass reflected fluid shifts or true tissue change. Our interpretation of the body composition results was tempered accordingly. That said, we agree the point warrants additional emphasis, and we have expanded the limitation. We also now specify that future trials should incorporate a multi-compartment (four-compartment) body composition model with total body water measured by isotope dilution to distinguish creatine-associated fluid shifts from true tissue accretion, and we note that the 10-week duration may itself be insufficient to detect true hypertrophic adaptations, which emerge over longer training periods. The revised text now reads as below:

*“Increases in intracellular fluid would be indicative of creatine update into the muscle, which would be supportive of muscle tissue hypertrophy changes with exercise. However, without a measure of body fluid (via multifrequency bioelectrical impedance), we cannot be confident what changes in body composition are from changes in total body water, versus true tissue changes [34, 102]. In addition, the 10-week duration may be insufficient to detect hypertrophic adaptations, which typically emerge over longer training periods; future trials should be of longer duration, powered for between-group comparisons, and should incorporate a multi-compartment (four-compartment) body composition model that includes total body water measured by isotope dilution (e.g., deuterium dilution or doubly labeled water), to distinguish CrM-associated fluid shifts from true tissue accretion. Finally, although the hybrid delivery model was feasible and well adhered to, the home-based equipment used for remote sessions may constrain progressive loading relative to in-clinic machines, which warrants consideration when designing the training stimulus for a future efficacy trial.”*

**Minor Comments
Ensure consistent terminology (CrM vs creatine vs creatine monohydrate).**

This has been revised for consistency (CrM abbreviated initially and used throughout the remainder of the paper).

**Some variability values in Table 2 appear unusually large (e.g., ALM SD appears inconsistent and should be checked).**

We thank the reviewer for identifying this issue. We rechecked Table 2 against the analysis dataset and confirmed that two descriptive standard deviations were transcription/decimal-placement errors in the table. Specifically, the placebo post-intervention lean body mass value should be reported as 48.90 ± 4.47 kg, not 48.90 ± 44.75 kg. The placebo baseline appendicular lean mass value should be reported as 20.90 ± 3.19 kg, not 20.90 ± 31.88 kg. Table 2 has been revised accordingly, and all values have been rechecked against the original dataset and analysis outputs.

**Clarify whether outcome assessors were blinded.**

This is stated in the methods: *“Investigators, outcome assessors, and data analysts remained blinded to the allocation sequence and group assignment throughout the study.*

**Provide more detail on qualitative analysis (e.g., thematic analysis approach, number of coders, inter-rater reliability).**

We have expanded the qualitative methods, and now specify that focus group data were analyzed using reflexive thematic analysis, proceeding through familiarization, initial coding, theme construction, and review. We have clarified that coding was conducted by a single researcher and that, consistent with reflexive thematic analysis, inter-rater reliability was not calculated, as coding is treated as an interpretative rather than a consensus-based process; trustworthiness was instead supported by reflexive journaling, an audit trail, and review of candidate themes with the research team.

**Clarify whether the trial was registered before participant enrollment.**

This has been revised to say: “The trial was registered at ClinicalTrials.gov (NCT06420726) prior to participant enrollment.”

**The CONSORT diagram should clearly show reasons for exclusion and dropout.**

The CONSORT diagram has been included with these details

**Line 122 states, citing three references, that this has nothing to do with cancer and that no studies have been conducted combining creatine supplementation with resistance training, but that is not true. It is necessary to mention in the introduction the studies that have used creatine supplementation in cancer patients and to also mention the few existing studies on creatine supplementation and strength training in cancer patients.**

We have revised the introduction to acknowledge the existing, though limited, literature combining creatine supplementation with resistance training in cancer populations, noting that these have been early-phase trials with small samples and inconclusive findings, and we now specify that this combination has not, to our knowledge, been examined in individuals treated for colorectal cancer. The revised text is below:

*“In individuals with cancer, a small but growing number of studies have combined CrM with RET, with early work examining functional and body composition outcomes across a limited range of cancer populations[44, 45]. To date, however, this evidence base remains limited, and to our knowledge it has not extended to individuals treated for colorectal cancer, a population in which such losses are frequent and in whom the feasibility of a combined CrM and RET approach has yet to be established.”*

**Were there no restrictions regarding gender and age? Age can be a variable that significantly influences muscle mass. Hormonal differences between men and women, particularly among the age groups of the study participants, significantly influence the response to training.**

Eligibility was not restricted by sex, and the age criterion was adults over 18-years so both sexes and a broad age range were included. This reflects the trial's aim as a feasibility pilot, which was to assess recruitment, retention, adherence, and safety across the general population of colorectal cancer survivors rather than to estimate efficacy in a homogeneous subgroup. Given the recruitment challenges we encountered (27 enrolled against a target of 40), further restriction by sex or age would have compounded an already difficult accrual.

We agree that age and sex, including sex-related hormonal differences, influence muscle mass and the response to resistance training. The original protocol accounted for this by stratifying randomization on age and sex; as disclosed in our protocol-changes section, stratification was removed to facilitate accrual. Baseline differences were addressed using baseline-adjusted ANCOVA, though the small sample precluded adjustment for additional covariates such as age and sex. Consistent with the exploratory framing of the secondary outcomes, the independent influence of age and sex therefore cannot be disentangled in this trial, and a future adequately powered trial should stratify by or adjust for these variables.

**Don’t you think that to determine whether creatine can help enhance the structural adaptations of strength training (hypertrophy), a longer intervention would be necessary?**

**Especially considering that one of the training sessions was conducted online.**

We agree that detecting whether creatine augments hypertrophic adaptations may require a longer and larger trial. This reflects the trial's purpose. As an NIH R21 pilot, it was designed to establish feasibility and generate estimates to justify a larger, longer, adequately powered efficacy trial, not to test structural efficacy. We have stated in the Discussion that such a trial should be of longer duration and incorporate direct measures of muscle size alongside total body water. On the remote session, hybrid delivery was one of the feasibility questions being tested, and adherence to the virtual sessions exceeded that of in-person sessions. We acknowledge that home equipment may constrain progressive loading relative to in-clinic machines, and we note this in the Discussion as a consideration for the future trial. The revised discussion section now reads:

*“In addition, the 10-week duration may be insufficient to detect hypertrophic adaptations, which typically emerge over longer training periods; future trials should be of longer duration, powered for between-group comparisons, and should incorporate a multi-compartment (four-compartment) body composition model that includes total body water measured by isotope dilution (e.g., deuterium dilution or doubly labeled water), to distinguish creatine-associated fluid shifts from true tissue accretion. Finally, although the hybrid delivery model was feasible and well adhered to, the home-based equipment used for remote sessions may constrain progressive loading relative to in-clinic machines, which warrants consideration when designing the training stimulus for a future efficacy trial.”*

**Sometimes it's written as “g/day” and other times as “grams/day”... we need to review and standardize the way it's written.**

This has been standardized to grams/day

**Wouldn't it make more sense to evaluate adherence—in addition to attendance—by looking at the number of sessions that met the planned training intensity and volume? After all, what's the point of showing up if you don't actually do the planned workout?**

We agree that attendance alone is an insufficient measure of adherence, which is why we report adherence in two ways: session attendance and the exercise relative dose intensity (RDI). As described in the Methods, RDI is the ratio of completed to prescribed training volume (sets × repetitions × load) and accounts for within-session dose modifications, interruptions, and missed sessions. It therefore captures the issue the reviewer raises, whether the planned training intensity and volume were actually achieved, rather than only whether participants attended. We would note that RDI is arguably more informative than a count of sessions meeting the prescribed dose, because it quantifies the proportion of the prescribed dose actually delivered and credits partial completion proportionally rather than classifying each session as simply met or not met. The de-identified per-participant training logs underlying these calculations are provided in S4.

**Although we cannot make comparisons between groups, given the extensive literature on the potentiating effect of creatine, why do the authors believe that the group that took creatine did not show greater improvement?**

We agree creatine has a well-documented potentiating effect, and we offer the following interpretation, noting that the trial was not powered for between-group comparisons and that these outcomes are framed as exploratory. The point estimates for most outcomes were directionally favorable to the creatine group, consistent with the literature, but did not reach a meaningful magnitude at this sample size; as stated in the Discussion, these null between-group effects should be interpreted cautiously rather than as evidence of no benefit, and the absence of a clear additive effect is consistent with our prior work in prostate cancer.

Several factors already discussed in the manuscript may have limited the emergence of a clearer between-group difference: the small sample and wide participant variability; the relatively high baseline physical function of the enrolled sample, with scores near the ceiling ; the 10-week duration, which may be too short for hypertrophic adaptations to emerge; and the inability of DXA to distinguish creatine-associated total body water from true tissue change. In addition, the fixed 5 g/day maintenance dose, adopted for real-world relevance in place of the original allometric dose, may have been sub-saturating for heavier participants. Consistent with the request to avoid causal language, we interpret these as plausible contributors rather than confirmed explanations.

**Reviewer #2: This two-arm randomized controlled pilot trial evaluated the feasibility, safety, and acceptability of a 10‑week hybrid resistance exercise training program with creatine supplementation versus placebo in colorectal cancer survivors. Twenty-seven participants were randomized, with high retention, adherence, and satisfaction, and no serious intervention-related adverse events. While no significant between-group differences were observed for secondary outcomes, both groups showed modest improvements in strength and physical function, supporting the feasibility of the intervention and the need for larger, adequately powered trials.

Minor suggested revisions:
1- Multiplicity not addressed: Multiple secondary outcomes were analyzed without adjustment for multiple comparisons. Although acceptable in a pilot study, this should be explicitly acknowledged as increasing type I error risk.**

As this was a pilot feasibility trial designed to estimate feasibility, safety, acceptability, and preliminary outcome variability rather than to test definitive efficacy hypotheses, we did not apply multiplicity adjustment to the secondary outcomes. We have now made this explicit and state that all secondary-outcome p values should be interpreted as nominal and exploratory. We also acknowledge that the absence of multiplicity correction increases the risk of type I error.

**2- Model assumptions not discussed: The ANCOVA analyses do not report checks or justification for assumptions (e.g., normality of residuals, homogeneity of regression slopes), which is especially relevant given the small sample size.**

We have revised the Statistical Analysis section to clarify that, for each ANCOVA model, we examined residual diagnostics and assessed homogeneity of regression slopes using the baseline-by-group interaction. These checks did not identify assumption concerns that changed the interpretation of the baseline-adjusted ANCOVA estimates. As an additional small-sample sensitivity check, we also computed robust standard errors and robust 95% confidence intervals for the group effect; the conclusions were unchanged.

**3- Missing data handling unclear: The approach to missing outcome data (e.g., complete-case analysis vs. imputation) is not explicitly described and should be clarified.**

We clarified that the analyses used outcome-specific complete cases, requiring available baseline and post-intervention values for the outcome being analyzed. No imputation was performed because this was a small pilot feasibility trial, and imputation would introduce additional modeling assumptions that would not be reliable at this sample size.

**4- Interpretation of p-values in an underpowered study: While generally cautious, some statistically significant findings (e.g., HRQOL financial difficulty subscale) should be more clearly contextualized as exploratory to avoid overinterpretation.**

We agree with the reviewer that statistically significant or nominally significant findings in this underpowered pilot trial should not be overinterpreted. We revised the language around the HRQOL financial difficulty subscale to identify it as a nominal, exploratory finding. We also revised the broader Results and Discussion language to avoid confirmatory efficacy claims and to emphasize that the secondary outcomes are hypothesis-generating.

**5- Baseline imbalance consideration: With small group sizes, some baseline differences may exist; a brief statement on how randomization balance was evaluated (or the rationale for relying solely on ANCOVA adjustment) would be useful.**

Baseline characteristics are summarized by randomized group in Table 1, and secondary-outcome analyses were adjusted for each outcome's corresponding baseline value using ANCOVA. We relied on baseline-adjusted ANCOVA because this approach is recommended for randomized trials with baseline and follow-up measurements and is more appropriate than unadjusted change-score comparisons for estimating between-group differences. Given the small sample, we did not add multiple additional covariates such as age and sex, because doing so would overfit the models and reduce precision. We now state this rationale more clearly.

**References**

1.	Moore SR, Baker PA, Smith-Ryan AE. Utility of fat-free adipose tissue correction formula for tracking body composition changes with dual-energy X-ray absorptiometry. Clin Physiol Funct Imaging. 2025;45(1):e12915. Epub 20241114. doi: 10.1111/cpf.12915. PubMed PMID: 39543795.

2.	Abe T, Loenneke JP, Thiebaud RS, Fujita E, Akamine T. The impact of DXA-derived fat-free adipose tissue on the prevalence of low muscle mass in older adults. European Journal of Clinical Nutrition. 2019;73(5):757–62. doi: 10.1038/s41430-018-0213-z.
