# Supplementary material for: Examining the feasibility and preliminary effects of resistance exercise training and creatine supplementation in individuals treated for colorectal cancer
Source: PLoS One. 2026 Jul 15;21(7):e0353630. doi: 10.1371/journal.pone.0353630 (PMC13372120; doi:10.1371/journal.pone.0353630)
Supplement: Peer review history

# Peer review: Decision Letter 0

**DOI:** 10.1371/journal.pone.0353630.r001

**Authors:** Diego A. Bonilla

15 Apr 2026

PONE-D-26-13357

Examining the Feasibility and Preliminary Effects of Resistance Exercise Training and Creatine Supplementation in Individuals Treated for Colorectal Cancer

PLOS One

Dear Dr. Fairman,

Thank you for submitting your manuscript to PLOS ONE. After careful consideration, we feel that it has merit but does not fully meet PLOS ONE’s publication criteria as it currently stands. Therefore, we invite you to submit a revised version of the manuscript that addresses the points raised during the review process.

Please submit your revised manuscript by May 30 2026 11:59PM. If you will need more time than this to complete your revisions, please reply to this message or contact the journal office at plosone@plos.org. When you’re ready to submit your revision, log on to https://www.editorialmanager.com/pone/ and select the 'Submissions Needing Revision' folder to locate your manuscript file.

Please include the following items when submitting your revised manuscript:

- A letter that responds to each point raised by the academic editor and reviewer(s). You should upload this letter as a separate file labeled 'Response to Reviewers'.
- A marked-up copy of your manuscript that highlights changes made to the original version. You should upload this as a separate file labeled 'Revised Manuscript with Track Changes'.
- An unmarked version of your revised paper without tracked changes. You should upload this as a separate file labeled 'Manuscript'.

If you would like to make changes to your financial disclosure, please include your updated statement in your cover letter. Guidelines for resubmitting your figure files are available below the reviewer comments at the end of this letter.

If applicable, we recommend that you deposit your laboratory protocols in protocols.io to enhance the reproducibility of your results. Protocols.io assigns your protocol its own identifier (DOI) so that it can be cited independently in the future. For instructions see: https://journals.plos.org/plosone/s/submission-guidelines#loc-laboratory-protocols. Additionally, PLOS ONE offers an option for publishing peer-reviewed Lab Protocol articles, which describe protocols hosted on protocols.io. Read more information on sharing protocols at https://plos.org/protocols?utm_medium=editorial-email&utm_source=authorletters&utm_campaign=protocols.

As the corresponding author, your ORCID iD is verified in the submission system and will appear in the published article. PLOS supports the use of ORCID, and we encourage all coauthors to register for an ORCID iD and use it as well. Please encourage your coauthors to verify their ORCID iD within the submission system before final acceptance, as unverified ORCID iDs will not appear in the published article. *Only*  the individual author can complete the verification step; PLOS staff *cannot*  verify ORCID iDs on behalf of authors.

We look forward to receiving your revised manuscript.

Kind regards,

**Prof. Diego A. Bonilla**

Academic Editor

PLOS One

Journal Requirements:

When submitting your revision, we need you to address these additional requirements.

1.Please ensure that your manuscript meets PLOS ONE's style requirements, including those for file naming. The PLOS ONE style templates can be found at

https://journals.plos.org/plosone/s/file?id=wjVg/PLOSOne_formatting_sample_main_body.pdf and

https://journals.plos.org/plosone/s/file?id=ba62/PLOSOne_formatting_sample_title_authors_affiliations.pdf

2. Thank you for stating the following in the Competing Interests section:

“DGC has conducted industry-sponsored research involving creatine supplementation and has received creatine donations for scientific studies and travel support for presentations related to creatine supplementation at scientific conferences. He serves on the Scientific Advisory Board for AlzChem (a manufacturer of creatine) and has acted as an expert witness and consultant in legal cases involving creatine supplementation. CMF reports consulting activities related to exercise oncology and resistance training for Inspire Exercise Medicine and has received speaker honoraria for AstraZeneca. The remaining authors declare that they have no relevant financial or non-financial interests to disclose. The results of this study are presented clearly, honestly, and without fabrication, falsification, or inappropriate data manipulation.”

We note that one or more of the authors are employed by a commercial company: AlzChem

1. Please provide an amended Funding Statement declaring this commercial affiliation, as well as a statement regarding the Role of Funders in your study. If the funding organization did not play a role in the study design, data collection and analysis, decision to publish, or preparation of the manuscript and only provided financial support in the form of authors' salaries and/or research materials, please review your statements relating to the author contributions, and ensure you have specifically and accurately indicated the role(s) that these authors had in your study. You can update author roles in the Author Contributions section of the online submission form.

Please also include the following statement within your amended Funding Statement.

“The funder provided support in the form of salaries for authors [insert relevant initials], but did not have any additional role in the study design, data collection and analysis, decision to publish, or preparation of the manuscript. The specific roles of these authors are articulated in the ‘author contributions’ section.”

If your commercial affiliation did play a role in your study, please state and explain this role within your updated Funding Statement.

2. Please also provide an updated Competing Interests Statement declaring this commercial affiliation along with any other relevant declarations relating to employment, consultancy, patents, products in development, or marketed products, etc.

Within your Competing Interests Statement, please confirm that this commercial affiliation does not alter your adherence to all PLOS ONE policies on sharing data and materials by including the following statement: "This does not alter our adherence to  PLOS ONE policies on sharing data and materials.” (as detailed online in our guide for authors http://journals.plos.org/plosone/s/competing-interests) . If this adherence statement is not accurate and  there are restrictions on sharing of data and/or materials, please state these. Please note that we cannot proceed with consideration of your article until this information has been declared.

Please include both an updated Funding Statement and Competing Interests Statement in your cover letter. We will change the online submission form on your behalf.

3. When completing the data availability statement of the submission form, you indicated that you will make your data available on acceptance. We strongly recommend all authors decide on a data sharing plan before acceptance, as the process can be lengthy and hold up publication timelines. Please note that, though access restrictions are acceptable now, your entire data will need to be made freely accessible if your manuscript is accepted for publication. This policy applies to all data except where public deposition would breach compliance with the protocol approved by your research ethics board. If you are unable to adhere to our open data policy, please kindly revise your statement to explain your reasoning and we will seek the editor's input on an exemption. Please be assured that, once you have provided your new statement, the assessment of your exemption will not hold up the peer review process.

4. Please upload a copy of Figure 1 and 2, to which you refer in your text on page 7 and 8. If the figure is no longer to be included as part of the submission please remove all reference to it within the text.

5. Please include a copy of Table 3-5 which you refer to in your text on page 16 and 18.

6. Please upload a copy of your study protocol that was approved by your ethics committee/IRB as a Supporting Information file. By the study protocol, we mean the complete and detailed plan for the conduct and analysis of the trial approved by the ethics committee/IRB. Please send this in the original language. If this is in a language other than English, please also provide a translation. [https://journals.plos.org/plosone/s/submission-guidelines#loc-guidelines-for-specific-study-types

7.If the reviewer comments include a recommendation to cite specific previously published works, please review and evaluate these publications to determine whether they are relevant and should be cited. There is no requirement to cite these works unless the editor has indicated otherwise.

Additional Editor Comments:

Please address all reviewers' comments and provide point-by-point responses.

This study is a valuable contribution to the literature; however, the manuscript requires improvements in structure and analysis to meet quality and transparency standards before publication can be considered.

MAJOR COMMENTS:

1. The authors describe several important changes to the study protocol (eligibility criteria, outcome assessments, intervention delivery, and supplementation dose) that deviate from the original ClinicalTrials.gov registration (NCT06420726). Could you please clarify why the trial record was not updated to reflect these changes? Timely updating of the registry entry is essential for transparency and adherence to best practices in clinical trial reporting.

Also, why is the entry registration in "Recruiting" state?

2. The structure of the manuscript, especially the METHODS section, needs to be revised. All subsections should be organized in strict order as recommended by CONSORT guidelines for pilot and feasibility studies (Trial design, Participants, Intervention Procedures, Outcomes, Sample Size, Statistical Analysis) as informed in the submission guidelines of PLoS One. Cf, https://pubmed.ncbi.nlm.nih.gov/31608150/ or https://www.equator-network.org/wp-content/uploads/2016/10/CONSORT-extension-Pilot-and-Feasibility-Trials-Checklist.doc

Replace the checklist with the correct CONSORT extension for pilot and feasibility studies (supplementary file 1).

3. Could the authors confirm whether the estimated variable of body composition reported in the manuscript is fat-free mass (lean body mass) or lean soft tissue?

As the authors are aware, there are relevant differences between the terms fat-free mass (FFM), lean soft tissue (LST) and skeletal muscle mass (SMM). As recommended recently by Heymsfield et al. (2024), 'to avoid confusion and improve scientific rigor, FFM is the preferred term for this body component (lean body mass), which is often measured with techniques using 2-compartment models such as TBW, body density, BIA, DXA, and 3D optical imaging.' Cf, https://pubmed.ncbi.nlm.nih.gov/39510253/

These terms might be mistakenly interchanged considering the DXA principle and measurements. Please refer to https://pubmed.ncbi.nlm.nih.gov/29786955/ and https://dexalytics.com/news/lean-soft-tissue-or-fat-free-mass/

4. In addition, did the authors correct data for fat-free adipose tissue (FFAT) before the analysis? If not, the analyses must be performed again. When using DXA, this is very important considering that the prevalence of low muscle mass and functionality in the US population is strongly affected by FFAT. Cf, https://pubmed.ncbi.nlm.nih.gov/27507068/, https://pubmed.ncbi.nlm.nih.gov/29915252/

5. Add the CV% of the instruments/technician to know the variability of all measures.

6. I agree with the reviewers regarding the statistical analysis. Please run a robust ANCOVA. Cf, https://pubmed.ncbi.nlm.nih.gov/18652737/

7. Generate figures with estimation plots to display the repeated measures data across time points (show individual data/responses at baseline and after creatine supplementation). Cumming or Gardner-Altman estimation plots are recommended.

Minor details:

1. RE, "DEXA" with "DXA". As recommended by the International Society for Clinical Densitometry (ISCD), DXA is the preferred abbreviation. Revise through the manuscript.

Cf, https://iscd.org/official-positions-2023/ (nomenclature) and https://pubmed.ncbi.nlm.nih.gov/27020004/

2. Revise the manuscript for typos. For example, all references are incorrectly placed after periods (e.g., .(1-3)).

[Note: HTML markup is below. Please do not edit.]

Reviewers' comments:

Reviewer's Responses to Questions

**Comments to the Author**

1. Is the manuscript technically sound, and do the data support the conclusions?

The manuscript must describe a technically sound piece of scientific research with data that supports the conclusions. Experiments must have been conducted rigorously, with appropriate controls, replication, and sample sizes. The conclusions must be drawn appropriately based on the data presented.

Reviewer #1: Yes

Reviewer #2: Yes

**********

2. Has the statistical analysis been performed appropriately and rigorously?

Reviewer #1: Yes

Reviewer #2: Yes

**********

3. Have the authors made all data underlying the findings in their manuscript fully available?

The PLOS Data policy requires authors to make all data underlying the findings described in their manuscript fully available without restriction, with rare exception (please refer to the Data Availability Statement in the manuscript PDF file). The data should be provided as part of the manuscript or its supporting information, or deposited to a public repository. For example, in addition to summary statistics, the data points behind means, medians and variance measures should be available. If there are restrictions on publicly sharing data—e.g. participant privacy or use of data from a third party—those must be specified.

Reviewer #1: Yes

Reviewer #2: Yes

**********

4. Is the manuscript presented in an intelligible fashion and written in standard English?

PLOS ONE does not copyedit accepted manuscripts, so the language in submitted articles must be clear, correct, and unambiguous. Any typographical or grammatical errors should be corrected at revision, so please note any specific errors here.

Reviewer #1: Yes

Reviewer #2: Yes

**********

5. Review Comments to the Author

Please use the space provided to explain your answers to the questions above. You may also include additional comments for the author, including concerns about dual publication, research ethics, or publication ethics. (Please upload your review as an attachment if it exceeds 20,000 characters)

Reviewer #1: General assessment

This manuscript presents a pilot randomized controlled trial evaluating the feasibility, safety, and acceptability of a 10-week hybrid resistance exercise training (RET) program with or without creatine monohydrate supplementation in individuals previously treated for colorectal cancer. The topic is relevant and important in the context of exercise oncology and cancer survivorship, particularly given the prevalence of sarcopenia, functional decline, and reduced quality of life following chemotherapy.

The manuscript is generally well written, the intervention is clearly described, and the feasibility outcomes are appropriate for a pilot trial. The inclusion of adherence, retention, acceptability, and adverse events is a strength, as is the hybrid delivery model, which has important clinical applicability.

However, there are several methodological and statistical issues that need clarification, and the interpretation of secondary outcomes should be more cautious given that the study is not powered for efficacy outcomes.

Overall, I recommend Major Revision.

Pilot study vs efficacy interpretation

The stated primary aim is feasibility, acceptability, and safety, which is appropriate for a pilot trial. However, in several sections (Results and Discussion), the manuscript interprets changes in strength, body composition, and physical function as meaningful intervention effects. Since both groups received RET and there was no non-exercise control group, the study cannot determine the efficacy of RET itself, nor definitively evaluate the additive effect of creatine given the small sample size.

The authors should emphasize that secondary outcomes are exploratory and avoid causal language suggesting effectiveness.

Protocol modifications

The manuscript reports several modifications to the original protocol. These are substantial changes and may affect internal validity and comparability with the original protocol.

The authors should clarify:

When these changes were made (before vs after recruitment began)

Whether these changes were updated in the trial registration

Whether these changes were approved by the IRB

How these changes may affect interpretation of feasibility and secondary outcomes

Recruitment and selection bias

The recruitment rate was very low (6.6%), and participants who enrolled appear relatively high functioning at baseline (SPPB ~10–11 out of 12). This suggests potential selection bias toward more motivated and physically capable participants, which limits generalizability to more vulnerable cancer survivors (e.g., sarcopenic, frail, recently treated patients).

This limitation should be discussed more explicitly.

Body composition measurement and creatine

Creatine supplementation may increase total body water, which can artificially increase lean body mass measured by DEXA. The authors mention this limitation briefly, but this is a critical methodological issue, particularly because lean mass is one of the main outcomes.

The authors should:

Expand this limitation

Clarify that changes in lean mass may reflect fluid shifts rather than true hypertrophy

Consider this when interpreting (discussion) body composition results

Minor Comments

Ensure consistent terminology (CrM vs creatine vs creatine monohydrate).

Some variability values in Table 2 appear unusually large (e.g., ALM SD appears inconsistent and should be checked).

Clarify whether outcome assessors were blinded.

Provide more detail on qualitative analysis (e.g., thematic analysis approach, number of coders, inter-rater reliability).

Clarify whether the trial was registered before participant enrollment.

The CONSORT diagram should clearly show reasons for exclusion and dropout.

Line 122 states, citing three references, that this has nothing to do with cancer and that no studies have been conducted combining creatine supplementation with resistance training, but that is not true. It is necessary to mention in the introduction the studies that have used creatine supplementation in cancer patients and to also mention the few existing studies on creatine supplementation and strength training in cancer patients.

Were there no restrictions regarding gender and age? Age can be a variable that significantly influences muscle mass. Hormonal differences between men and women, particularly among the age groups of the study participants, significantly influence the response to training.

Don’t you think that to determine whether creatine can help enhance the structural adaptations of strength training (hypertrophy), a longer intervention would be necessary? Especially considering that one of the training sessions was conducted online.

Sometimes it's written as “g/day” and other times as “grams/day”... we need to review and standardize the way it's written.

Wouldn't it make more sense to evaluate adherence—in addition to attendance—by looking at the number of sessions that met the planned training intensity and volume? After all, what's the point of showing up if you don't actually do the planned workout?

The references appear in a different format

Although we cannot make comparisons between groups, given the extensive literature on the potentiating effect of creatine, why do the authors believe that the group that took creatine did not show greater improvement?

Reviewer #2: This two-arm randomized controlled pilot trial evaluated the feasibility, safety, and acceptability of a 10‑week hybrid resistance exercise training program with creatine supplementation versus placebo in colorectal cancer survivors. Twenty-seven participants were randomized, with high retention, adherence, and satisfaction, and no serious intervention-related adverse events. While no significant between-group differences were observed for secondary outcomes, both groups showed modest improvements in strength and physical function, supporting the feasibility of the intervention and the need for larger, adequately powered trials.

Minor suggested revisions:

1- Multiplicity not addressed: Multiple secondary outcomes were analyzed without adjustment for multiple comparisons. Although acceptable in a pilot study, this should be explicitly acknowledged as increasing type I error risk.

2- Model assumptions not discussed: The ANCOVA analyses do not report checks or justification for assumptions (e.g., normality of residuals, homogeneity of regression slopes), which is especially relevant given the small sample size.

3- Missing data handling unclear: The approach to missing outcome data (e.g., complete-case analysis vs. imputation) is not explicitly described and should be clarified.

4- Interpretation of p-values in an underpowered study: While generally cautious, some statistically significant findings (e.g., HRQOL financial difficulty subscale) should be more clearly contextualized as exploratory to avoid overinterpretation.

5- Baseline imbalance consideration: With small group sizes, some baseline differences may exist; a brief statement on how randomization balance was evaluated (or the rationale for relying solely on ANCOVA adjustment) would be useful.

**********

6. PLOS authors have the option to publish the peer review history of their article (what does this mean?). If published, this will include your full peer review and any attached files.

If you choose “no”, your identity will remain anonymous but your review may still be made public.

**Do you want your identity to be public for this peer review?**  For information about this choice, including consent withdrawal, please see our Privacy Policy.

Reviewer #1: No

Reviewer #2: No

**********

[NOTE: If reviewer comments were submitted as an attachment file, they will be attached to this email and accessible via the submission site. Please log into your account, locate the manuscript record, and check for the action link "View Attachments". If this link does not appear, there are no attachment files.]

To ensure your figures meet our technical requirements, please review our figure guidelines: https://journals.plos.org/plosone/s/figures

You may also use PLOS’s free figure tool, NAAS, to help you prepare publication quality figures: https://journals.plos.org/plosone/s/figures#loc-tools-for-figure-preparation.

NAAS will assess whether your figures meet our technical requirements by comparing each figure against our figure specifications.

# Author comment: Author response to Decision Letter 1

**DOI:** 10.1371/journal.pone.0353630.r002

16 Jun 2026

When submitting your revision, we need you to address these additional requirements.

1.Please ensure that your manuscript meets PLOS ONE's style requirements, including those for file naming. The PLOS ONE style templates can be found at

https://journals.plos.org/plosone/s/file?id=wjVg/PLOSOne_formatting_sample_main_body.pdf and

https://journals.plos.org/plosone/s/file?id=ba62/PLOSOne_formatting_sample_title_authors_affiliations.pdf

We have verified that the revised manuscript adheres to the Style Templates.

2. Thank you for stating the following in the Competing Interests section:

“DGC has conducted industry-sponsored research involving creatine supplementation and has received creatine donations for scientific studies and travel support for presentations related to creatine supplementation at scientific conferences. He serves on the Scientific Advisory Board for AlzChem (a manufacturer of creatine) and has acted as an expert witness and consultant in legal cases involving creatine supplementation. CMF reports consulting activities related to exercise oncology and resistance training for Inspire Exercise Medicine and has received speaker honoraria for AstraZeneca. The remaining authors declare that they have no relevant financial or non-financial interests to disclose. The results of this study are presented clearly, honestly, and without fabrication, falsification, or inappropriate data manipulation.” We note that one or more of the authors are employed by a commercial company: AlzChem

1. Please provide an amended Funding Statement declaring this commercial affiliation, as well as a statement regarding the Role of Funders in your study. If the funding organization did not play a role in the study design, data collection and analysis, decision to publish, or preparation of the manuscript and only provided financial support in the form of authors' salaries and/or research materials, please review your statements relating to the author contributions, and ensure you have specifically and accurately indicated the role(s) that these authors had in your study. You can update author roles in the Author Contributions section of the online submission form.

Please also include the following statement within your amended Funding Statement.

“The funder provided support in the form of salaries for authors [insert relevant initials], but did not have any additional role in the study design, data collection and analysis, decision to publish, or preparation of the manuscript. The specific roles of these authors are articulated in the ‘author contributions’ section.” If your commercial affiliation did play a role in your study, please state and explain this role within your updated Funding Statement.

DGC is not employed by AlzChem. He serves on AlzChem's Scientific Advisory Board and is a paid consultant, both of which are disclosed in the Competing Interests statement. AlzChem's only contribution to this study was the in-kind provision of the creatine and matched placebo; it had no role in the study design, data collection and analysis, decision to publish, or preparation of the manuscript. We have amended the Funding Statement and Competing Interests accordingly.

Revised Funding Statement

This work was supported by the National Cancer Institute of the National Institutes of Health under Award Number R21CA281951-01. The content is solely the responsibility of the authors and does not necessarily represent the official views of the National Institutes of Health. Creatine monohydrate (Creapure®) and the matched placebo were provided by AlzChem Trostberg GmbH (Trostberg, Germany). This publication was also supported by the National Institute Of General Medical Sciences of the National Institutes of Health under Award Number P20GM155896 through the Center for Clinical & Translational Research at University of South Carolina (ClinTrUSC). The content is solely the responsibility of the authors and does not necessarily represent the official views of the National Institutes of Health. The funders had no role in the study design, data collection and analysis, decision to publish, or preparation of the manuscript.

2. Please also provide an updated Competing Interests Statement declaring this commercial affiliation along with any other relevant declarations relating to employment, consultancy, patents, products in development, or marketed products, etc. Within your Competing Interests Statement, please confirm that this commercial affiliation does not alter your adherence to all PLOS ONE policies on sharing data and materials by including the following statement: "This does not alter our adherence to PLOS ONE policies on sharing data and materials.” (as detailed online in our guide for authors http://journals.plos.org/plosone/s/competing-interests) . If this adherence statement is not accurate and there are restrictions on sharing of data and/or materials, please state these. Please note that we cannot proceed with consideration of your article until this information has been declared.

We confirm that there are no restrictions on the sharing of data or materials associated with this study. We have added the requested statement verbatim to the Competing Interests section

Revised Competing Interests Statement

DGC has conducted industry-sponsored research involving creatine supplementation and has received creatine donations for scientific studies and travel support for presentations related to creatine supplementation at scientific conferences. He serves on the Scientific Advisory Boards of AlzChem, Create, and Lovate, and is a paid consultant to AlzChem (companies that manufacture creatine). AlzChem provided the creatine monohydrate and matched placebo used in this study but had no role in the study design, data collection and analysis, decision to publish, or preparation of the manuscript. CMF reports consulting activities related to exercise oncology and resistance training for Inspire Exercise Medicine and has received speaker honoraria from AstraZeneca. The remaining authors declare that they have no relevant financial or non-financial interests to disclose. This does not alter our adherence to PLOS ONE policies on sharing data and materials. The results of this study are presented clearly, honestly, and without fabrication, falsification, or inappropriate data manipulation.

Please include both an updated Funding Statement and Competing Interests Statement in your cover letter. We will change the online submission form on your behalf.

As outlined above, we have amended both the Funding Statement and Competing Interests Statement and have included them in the cover letter with the resubmission.

3. When completing the data availability statement of the submission form, you indicated that you will make your data available on acceptance. We strongly recommend all authors decide on a data sharing plan before acceptance, as the process can be lengthy and hold up publication timelines. Please note that, though access restrictions are acceptable now, your entire data will need to be made freely accessible if your manuscript is accepted for publication. This policy applies to all data except where public deposition would breach compliance with the protocol approved by your research ethics board. If you are unable to adhere to our open data policy, please kindly revise your statement to explain your reasoning and we will seek the editor's input on an exemption. Please be assured that, once you have provided your new statement, the assessment of your exemption will not hold up the peer review process.

Our Data availability statement does not say the data will be available upon acceptance. The data is publicly available, and the data availability statement reflects this:

“All data reported in this manuscript are publicly available. Deidentified participant-level data, metadata (data dictionary), and analysis code underlying the study findings are available in a public repository hosted on GitHub: https://github.com/zhangtiejun310/R21_Resistance-Exercise-Training-and-Creatine-Supplementation. The repository contains the dataset used for analysis, accompanying documentation, and scripts necessary to reproduce the results.”

4. Please upload a copy of Figure 1 and 2, to which you refer in your text on page 7 and 8. If the figure is no longer to be included as part of the submission please remove all reference to it within the text.

We have included these figures in the submission

5. Please include a copy of Table 3-5 which you refer to in your text on page 16 and 18.

These 3 Tables are tables in Supplementary File S2. For example “S2, Table 3” refers to the third table in Supplementary File S2.

6. Please upload a copy of your study protocol that was approved by your ethics committee/IRB as a Supporting Information file. By the study protocol, we mean the complete and detailed plan for the conduct and analysis of the trial approved by the ethics committee/IRB. Please send this in the original language. If this is in a language other than English, please also provide a translation. [https://journals.plos.org/plosone/s/submission-guidelines#loc-guidelines-for-specific-study-types

We have updated file S3 for the resubmission to be the protocol rather than the IRB approval letter.

MAJOR COMMENTS:

1. The authors describe several important changes to the study protocol (eligibility criteria, outcome assessments, intervention delivery, and supplementation dose) that deviate from the original ClinicalTrials.gov registration (NCT06420726). Could you please clarify why the trial record was not updated to reflect these changes? Timely updating of the registry entry is essential for transparency and adherence to best practices in clinical trial reporting. Also, why is the entry registration in "Recruiting" state?

We thank the reviewer for raising this point. We agree that timely updating of the trial registry is essential for transparency and adherence to best practice in clinical trial reporting, and we apologize that the ClinicalTrials.gov record (NCT06420726) was not kept current with the protocol amendments at the time of submission. As amendments were made over the course of the trial, the corresponding updates were not entered into the ClinicalTrials.gov record, and this lapse was not caught by the study team. As a result, the public record continued to display the original protocol and an active recruitment status. This was an administrative oversight on our part rather than an intentional omission. We have now updated the record in full. The registry has been revised to reflect each of the amendments described in the manuscript.

Regarding the reviewer's second question, the "Recruiting" status was a direct consequence of the record not having been updated; it did not reflect the true state of the trial, which completed data collection in June 2025. The overall status has now been changed to "Completed," and the actual enrollment (n = 27) and study completion date have been entered.

Finally, we note that, independent of the registry, every protocol amendment and its rationale were disclosed in the submitted manuscript. These are described in the "Changes to Study Protocol" section and itemized against the original protocol language in S2 (Table 1). We hope that the corrected registry entry, together with this in-manuscript documentation, provides complete transparency regarding the evolution of the protocol.

2. The structure of the manuscript, especially the METHODS section, needs to be revised. All subsections should be organized in strict order as recommended by CONSORT guidelines for pilot and feasibility studies (Trial design, Participants, Intervention Procedures, Outcomes, Sample Size, Statistical Analysis) as informed in the submission guidelines of PLoS One. Cf, https://pubmed.ncbi.nlm.nih.gov/31608150/ or https://www.equator-network.org/wp-content/uploads/2016/10/CONSORT-extension-Pilot-and-Feasibility-Trials-Checklist.doc Replace the checklist with the correct CONSORT extension for pilot and feasibility studies (supplementary file 1).

We thank the reviewer. We have restructured the Methods section to follow the order recommended by the CONSORT extension for randomised pilot and feasibility trials, with the subsections now presented as Trial design, Participants, Intervention Procedures, Outcomes, Sample Size, and Statistical Analysis. We have also replaced the checklist in Supplementary File 1 with the correct CONSORT 2010 extension to randomised pilot and feasibility trials checklist, completed for this manuscript and cross-referenced to the relevant sections.

3. Could the authors confirm whether the estimated variable of body composition reported in the manuscript is fat-free mass (lean body mass) or lean soft tissue? As the authors are aware, there are relevant differences between the terms fat-free mass (FFM), lean soft tissue (LST) and skeletal muscle mass (SMM). As recommended recently by Heymsfield et al. (2024), 'to avoid confusion and improve scientific rigor, FFM is the preferred term for this body component (lean body mass), which is often measured with techniques using 2-compartment models such as TBW, body density, BIA, DXA, and 3D optical imaging.' Cf, https://pubmed.ncbi.nlm.nih.gov/39510253/ These terms might be mistakenly interchanged considering the DXA principle and measurements. Please refer to https://pubmed.ncbi.nlm.nih.gov/29786955/ and https://dexalytics.com/news/lean-soft-tissue-or-fat-free-mass/

We confirm that the DXA-derived values reported are lean soft tissue (LST), measured with the Hologic Horizon A: whole-body lean soft tissue and appendicular lean soft tissue (ALM), both of which exclude bone mineral content.

4. In addition, did the authors correct data for fat-free adipose tissue (FFAT) before the analysis? If not, the analyses must be performed again. When using DXA, this is very important considering that the prevalence of low muscle mass and functionality in the US population is strongly affected by FFAT. Cf, https://pubmed.ncbi.nlm.nih.gov/27507068/, https://pubmed.ncbi.nlm.nih.gov/29915252/

We thank the reviewer for raising the influence of fat-free adipose tissue (FFAT) on DXA-derived lean mass. We would respectfully note that the references cited are cross-sectional studies concerned with the prevalence and classification of low muscle mass, in which uncorrected appendicular lean mass is inflated at higher adiposity and can shift individuals across diagnostic cut-points. The present study does not estimate prevalence or classify participants against muscle-mass thresholds; it reports baseline-adjusted between-group differences in continuous lean mass within a randomized trial.

The relevance of the FFAT correction to this longitudinal, between-group context was examined directly by Moore et al. (2024)[1], who applied the same correction formula[2] across 226 participants in five exercise and nutrition trials. They reported that corrected and uncorrected lean-mass change scores diverged substantially only in cases of large body-mass loss (>10%, e.g., following bariatric surgery); the only other condition reaching significance was modest recomposition, where the difference between methods was nonetheless trivial in magnitude (on the order of 0.1 kg) and did not alter study conclusions. The authors concluded that for most populations and intervention types DXA-derived values are accurate and the correction may not be necessary. Our trial delivered resistance training with creatine, and no participant approached the degree of body-mass change at which the correction becomes consequential (the largest body-mass change observed was approximately 5%). On this basis, the FFAT correction would not be expected to meaningfully affect the between-group estimates reported here, and we do not believe re-analysis is warranted for the present design. We appreciate the reviewer's attention to this issue, and we hope the reviewer agrees that re-analysis is not warranted given the design and population of this trial.

5. Add the CV% of the instruments/technician to know the variability of all measures.

We have added precision and reliability information

Attachment Submitted filename: Response to Reviewers.docx

# Peer review: Decision Letter 1

**DOI:** 10.1371/journal.pone.0353630.r003

**Authors:** Diego A. Bonilla, Diego A. Bonilla

25 Jun 2026

Examining the feasibility and preliminary effects of resistance exercise training and creatine supplementation in individuals treated for colorectal cancer

PONE-D-26-13357R1

Dear Dr. Fairman,

We’re pleased to inform you that your manuscript has been judged scientifically suitable for publication and will be formally accepted for publication once it meets all outstanding technical requirements.

Within one week, you’ll receive an e-mail detailing the required amendments. When these have been addressed, you’ll receive a formal acceptance letter and your manuscript will be scheduled for publication.

An invoice will be generated when your article is formally accepted. Please note, if your institution has a publishing partnership with PLOS and your article meets the relevant criteria, all or part of your publication costs will be covered. Please make sure your user information is up-to-date by logging into Editorial Manager at Editorial Manager® and clicking the ‘Update My Information' link at the top of the page. For questions related to billing, please contact billing support.

If your institution or institutions have a press office, please notify them about your upcoming paper to help maximize its impact. If they’ll be preparing press materials, please inform our press team as soon as possible -- no later than 48 hours after receiving the formal acceptance. Your manuscript will remain under strict press embargo until 2 pm Eastern Time on the date of publication. For more information, please contact onepress@plos.org.

Kind regards,

**Prof. Diego A. Bonilla**

Academic Editor

PLOS One

Additional Editor Comments (optional):

Reviewers' comments:

Reviewer's Responses to Questions

**Comments to the Author**

1. If the authors have adequately addressed your comments raised in a previous round of review and you feel that this manuscript is now acceptable for publication, you may indicate that here to bypass the “Comments to the Author” section, enter your conflict of interest statement in the “Confidential to Editor” section, and submit your "Accept" recommendation.

Reviewer #1: All comments have been addressed

Reviewer #2: All comments have been addressed

**********

2. Is the manuscript technically sound, and do the data support the conclusions?

The manuscript must describe a technically sound piece of scientific research with data that supports the conclusions. Experiments must have been conducted rigorously, with appropriate controls, replication, and sample sizes. The conclusions must be drawn appropriately based on the data presented.

Reviewer #1: Yes

Reviewer #2: (No Response)

**********

3. Has the statistical analysis been performed appropriately and rigorously?

Reviewer #1: Yes

Reviewer #2: (No Response)

**********

4. Have the authors made all data underlying the findings in their manuscript fully available?

The PLOS Data policy requires authors to make all data underlying the findings described in their manuscript fully available without restriction, with rare exception (please refer to the Data Availability Statement in the manuscript PDF file). The data should be provided as part of the manuscript or its supporting information, or deposited to a public repository. For example, in addition to summary statistics, the data points behind means, medians and variance measures should be available. If there are restrictions on publicly sharing data—e.g. participant privacy or use of data from a third party—those must be specified.

Reviewer #1: Yes

Reviewer #2: (No Response)

**********

5. Is the manuscript presented in an intelligible fashion and written in standard English?

PLOS ONE does not copyedit accepted manuscripts, so the language in submitted articles must be clear, correct, and unambiguous. Any typographical or grammatical errors should be corrected at revision, so please note any specific errors here.

Reviewer #1: Yes

Reviewer #2: (No Response)

**********

6. Review Comments to the Author

Please use the space provided to explain your answers to the questions above. You may also include additional comments for the author, including concerns about dual publication, research ethics, or publication ethics. (Please upload your review as an attachment if it exceeds 20,000 characters)

Reviewer #1: The authors have satisfactorily addressed all comments and suggestions raised during the review process. The revisions made have improved the clarity, structure, and overall quality of the manuscript, adequately addressing the reviewers’ observations. I consider the responses provided to be appropriate and sufficiently detailed, and the changes made to strengthen the scientific rigor of the work. In its current version, the manuscript demonstrates an adequate level of methodological rigor and presentation of results, and therefore meets the necessary criteria for publication. I would like to congratulate the authors on their efforts during the review process and on their willingness to incorporate the recommendations received, which has contributed to a significant improvement in the final quality of the article.

Reviewer #2: (No Response)

**********

7. PLOS authors have the option to publish the peer review history of their article (what does this mean?). If published, this will include your full peer review and any attached files.

If you choose “no”, your identity will remain anonymous but your review may still be made public.

**Do you want your identity to be public for this peer review?**  For information about this choice, including consent withdrawal, please see our Privacy Policy.

Reviewer #1: No

Reviewer #2: No

**********

# Editor report: Acceptance letter

**DOI:** 10.1371/journal.pone.0353630.r004

**Authors:** Diego A. Bonilla, Diego A. Bonilla

PONE-D-26-13357R1

PLOS One

Dear Dr. Fairman,

I'm pleased to inform you that your manuscript has been deemed suitable for publication in PLOS One. Congratulations! Your manuscript is now being handed over to our production team.

At this stage, our production department will prepare your paper for publication. This includes ensuring the following:

* All references, tables, and figures are properly cited

* All relevant supporting information is included in the manuscript submission,

* There are no issues that prevent the paper from being properly typeset

You will receive further instructions from the production team, including instructions on how to review your proof when it is ready. Please keep in mind that we are working through a large volume of accepted articles, so please give us a few days to review your paper and let you know the next and final steps.

Lastly, if your institution or institutions have a press office, please let them know about your upcoming paper now to help maximize its impact. If they'll be preparing press materials, please inform our press team within the next 48 hours. Your manuscript will remain under strict press embargo until 2 pm Eastern Time on the date of publication. For more information, please contact onepress@plos.org.

You will receive an invoice from PLOS for your publication fee after your manuscript has reached the completed accept phase. If you receive an email requesting payment before acceptance or for any other service, this may be a phishing scheme. Learn how to identify phishing emails and protect your accounts at https://explore.plos.org/phishing.

If we can help with anything else, please email us at customercare@plos.org.

Thank you for submitting your work to PLOS ONE and supporting open access.

Kind regards,

PLOS ONE Editorial Office Staff

on behalf of

Prof. Diego A. Bonilla

Academic Editor

PLOS One
